# A Possible Mechanism behind Autoimmune Disorders Discovered By Genome-Wide Linkage and Association Analysis in Celiac Disease

**DOI:** 10.1371/journal.pone.0070174

**Published:** 2013-08-02

**Authors:** Malin Östensson, Caroline Montén, Jonas Bacelis, Audur H. Gudjonsdottir, Svetlana Adamovic, Johan Ek, Henry Ascher, Elisabet Pollak, Henrik Arnell, Lars Browaldh, Daniel Agardh, Jan Wahlström, Staffan Nilsson, Åsa Torinsson-Naluai

**Affiliations:** 1 Department of Mathematical Sciences, Chalmers University of Technology, Gothenburg, Sweden; 2 Diabetes and Celiac Disease Unit, Department of Clinical Sciences, Lund University, Malmö, Sweden; 3 Institute of Biomedicine, Department of Medical and Clinical Genetics, Sahlgrenska Academy at the University of Gothenburg, Gothenburg, Sweden; 4 Queen Silvia Children’s Hospital, Sahlgrenska Academy at the University of Gothenburg, Department of Pediatrics, Gothenburg, Sweden; 5 Buskerud Central Hospital, Department of Pediatrics, Drammen, Norway; 6 Sahlgrenska Academy at the University of Gothenburg, Department of Public Health and Community Medicine, Unit of Social Medicine, Gothenburg, Sweden; 7 Department of Pediatric Gastroenterology, Hepatology and Nutrition, Karolinska University Hospital and Division of Pediatrics, CLINTEC, Karolinska Institutet, Stockholm, Sweden; 8 Department of Clinical Science and Education, Karolinska Institutet Sodersjukhuset, Stockholm, Sweden; 9 Systems Biology Research Centre, Tumor Biology, School of Life Sciences University of Skövde, Skövde, Sweden; Albert Einstein Institute for Research and Education, Brazil

## Abstract

Celiac disease is a common autoimmune disorder characterized by an intestinal inflammation triggered by gluten, a storage protein found in wheat, rye and barley. Similar to other autoimmune diseases such as type 1 diabetes, psoriasis and rheumatoid arthritis, celiac disease is the result of an immune response to self-antigens leading to tissue destruction and production of autoantibodies. Common diseases like celiac disease have a complex pattern of inheritance with inputs from both environmental as well as additive and non-additive genetic factors. In the past few years, Genome Wide Association Studies (GWAS) have been successful in finding genetic risk variants behind many common diseases and traits. To complement and add to the previous findings, we performed a GWAS including 206 trios from 97 nuclear Swedish and Norwegian families affected with celiac disease. By stratifying for HLA-DQ, we identified a new genome-wide significant risk locus covering the *DUSP10* gene. To further investigate the associations from the GWAS we performed pathway analyses and two-locus interaction analyses. These analyses showed an over-representation of genes involved in type 2 diabetes and identified a set of candidate mechanisms and genes of which some were selected for mRNA expression analysis using small intestinal biopsies from 98 patients. Several genes were expressed differently in the small intestinal mucosa from patients with celiac autoimmunity compared to intestinal mucosa from control patients. From top-scoring regions we identified susceptibility genes in several categories: 1) polarity and epithelial cell functionality; 2) intestinal smooth muscle; 3) growth and energy homeostasis, including proline and glutamine metabolism; and finally 4) innate and adaptive immune system. These genes and pathways, including specific functions of *DUSP10*, together reveal a new potential biological mechanism that could influence the genesis of celiac disease, and possibly also other chronic disorders with an inflammatory component.

## Introduction

Celiac disease (CD) is a common chronic disease and even though most often diagnosed in early childhood, it can present itself at any age. Most of the individuals with CD remain undiagnosed and an estimated 2% of the Swedish population is affected without having been diagnosed [1]. Ongoing disease will increase the overall risk for developing other chronic inflammatory diseases, neurological manifestations and malnutrition disorders. CD is the only autoimmune disorder where the actual genes responsible for the association in HLA are known (*HLA-DQA1* and *HLA-DQB1*) [2]. In the past few years Genome Wide Association Studies (GWAS) have had tremendous success in identifying new genes, or gene regions, that influence common diseases. These studies use several hundreds of thousands of genetic markers (single nucleotide polymorphisms, SNPs) across all human chromosomes in order to pin down the chromosomal locations of genes, which could influence the disease.

A large joint effort has been done, not the least in CD, and 40 new CD-associated genetic regions marked by SNPs have been discovered [3]–[7]. However, these genes cannot account for all CD heritability, and part of the genetic variance that influences disease development is still unknown [8].

Most GWAS so far have been performed on case control samples. A case control study design has some advantages compared to using a family study design. For example, in a case control design it is possible to select a perfectly matched set of controls to increase the chance of discovering susceptibility genes, and furthermore, cases and controls are usually easier to collect than individuals from the same family. However, using a family material can be a very good complement to a case control design. First of all, families with several affected members are likely to have a stronger genetic component compared to sporadic cases. Familial cases tend to be enriched for disease-predisposing alleles and there is an increased power especially for detecting rare genetic variants [9]. Another important fact is that statistical analyses based on family data are robust against population stratification. Already in their paper from 1996, Risch and Merikangas suggested that all sib-pair families collected for non-parametric linkage analysis in complex diseases, should be re-run “Genome-Wide” using SNP markers and the potentially more powerful Transmission Disequilibrium Test (TDT) [10]. The TDT test in sib-pairs is a test of linkage in the presence of association. Hereafter we refer to whole genome sibling TDT as “Linkage GWAS”.

In this study, we aimed to uncover additional genetic factors in CD by performing a Linkage GWAS using 206 affected children (sib-pairs) within 97 nuclear families using the TDT test. In addition to the Linkage GWAS we explored gene-gene interactions and pathway analyses. We also performed a non-parametric linkage (NPL) analysis and compared the results with the published linkage analysis, with microsatellite markers, performed in the same set of families previously [11]. Furthermore, quantitative PCR was used to investigate levels of gene expression in small intestinal biopsies from additional patients with CD autoimmunity (CAI) and control patients. Finally, we stratified the TDT analysis on HLA genotype. It has been shown that carrying DQB1*02 on both chromosomes (i.e. being homozygous), confers higher risk of developing CD as compared to heterozygote individuals [12]. It is therefore conceivable that heterozygote individuals may require more additional risk factors outside HLA, in order to accumulate sufficient risk to develop CD, compared with homozygous individuals. Based on this assumption we stratified the patient in an HLA low-risk group and an HLA high-risk group. By stratifying the Linkage GWAS, we expected to uncover even more of the so-called “missing heritability” in CD. This strategy could identify different risk factors all together or perhaps a more likely scenario is that the same risk factors outside HLA would just be more common in the HLA low-risk group.

## Results

### Genotyping and Imputation

We included single nucleotide polymorphism (SNP) markers that had a call rate above 97%, which led to the exclusion of 1.3% of the Omni Express and 0.6% of the 660W-Quad SNP markers. Out of the 127,535,126 imputed genotypes, 88.3% had a posterior probability of over 0.95. Approximately 90% of the 944,512 SNP markers had a minor allele frequency of at least 0.01 after imputation.

### Transmission Disequilibrium Test (TDT)

**Figure 1:**
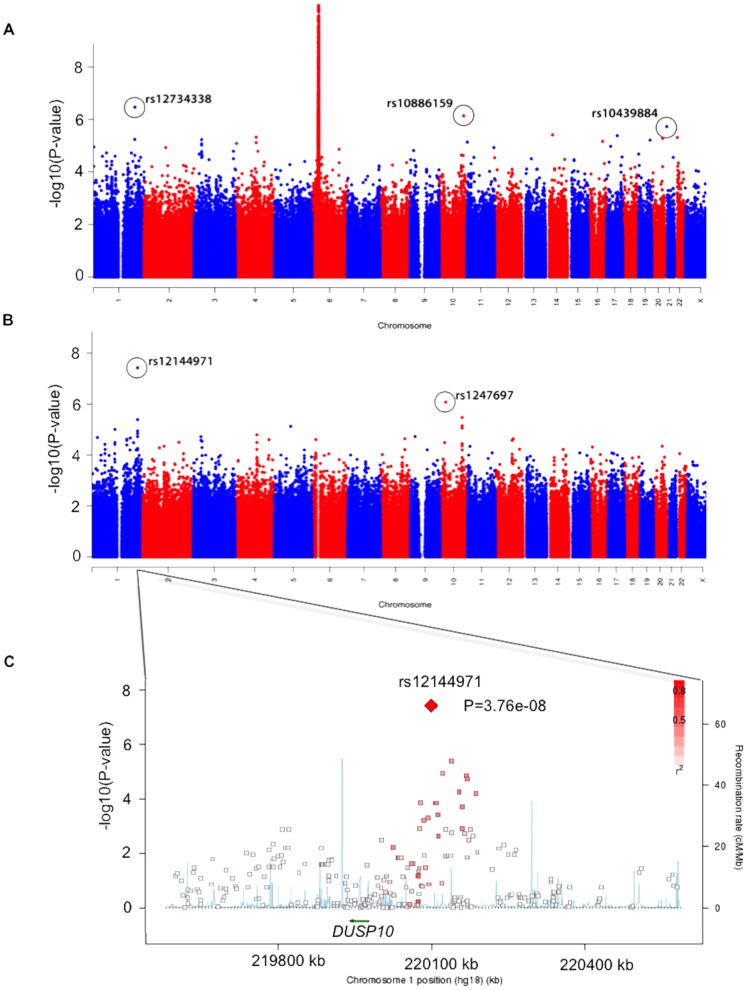
Manhattanplot of the TDT p-values. a) The location of all genotyped SNPs on chromosomes 1–22 and X plotted on the x-axis. –log10(p-value) result for each SNP and all transmissions on the y-axis. b) The location of all genotyped SNPs on chromosomes 1–22 and X plotted on the x-axis. –log10(p-value) result for each SNP and all transmissions, to children in the low risk group, on the y-axis. c) Regional plot of association results and recombination rates, within the region surrounding *DUSP10,* generated by SNAP (http://www.broadinstitute.org/mpg/snap/ldplot.php). The x-axis show 500 kb around the most associated SNP. Genomic locations of genes within the region of interest (NCBI Build 36 human assembly) were annotated from the UCSC Genome Browser (arrows). The left y-axis show –log10(p-value) and estimated recombination rates (cM/Mb) from HapMap Project (NCBI Build 36) are shown in light blue lines.

All markers from the TDT analysis are shown in Figure 1a. As expected, the region around the CD associated *HLA* genes on chromosome 6 showed the strongest association with the most significant p-value reaching 4.9×10^−21^ at marker rs424232. In Table 1, we present the 35 most significant associations found outside of HLA (HLA defined as SNP markers located within 27–34 Mb on chromosome 6). The most significant finding outside of the HLA region was the marker rs12734338 on chromosome 1, including the *PPP1R12B* gene.

**Table 1:** Transmission Disequilibrium Test (TDT).

						TDT (PLINK)			exp TDT					
Chr	SNP	Genes	BP	A1	A2	T	U	p-value	T	U	T/U	p-value	NPL	GWAS catalog
1	rs12734338	PPP1R12B SYT2 UBE2T	200736346	C	T	39	90	7.11E-06	61.44	132.45	0.46	3.41E-07	*0,0095*	
10	rs10886159	EMX2OS RAB11FIP2 EMX2	119603600	C	T	24	50	2.51E-03	40.16	98.48	0.41	7.30E-07	0,19*	
21	rs10439884	BAGE2 TPTE BAGE	9993822	A	G	10	23	2.36E-02	15.52	55.78	0.28	1.86E-06	0,22*	
14	rs1958589	EAPP SNX6 C14orf147	33914127	C	T	15	36	3.28E-03	27.25	73.64	0.37	3.87E-06	0,32*	
17	rs17760268	ANKFN1 NOG	51966290	C	T	27	11	9.44E-03	57.24	17.45	3.28	4.13E-06	0,33*	Cannabis depend./height
4	rs1032355	RG9MTD2 C4orf17 MTTP	100758919	C	T	33	81	6.94E-06	35.07	85.28	0.41	4.74E-06	*0,0069* *	
22	rs4911642	CCT8L2 psiTPTE22	14884399	C	T	20	38	1.81E-02	34.38	84.15	0.41	4.86E-06	0,21*	HIV-1 viral setpoint
20	rs157640	DOK5	52847946	G	T	71	135	8.23E-06	72	138	0.52	5.25E-06	0,38	Functional MRI
1	rs2068824	NAV1	199861288	C	T	2	16	9.67E-04	6.83	36.77	0.19	5.75E-06	0,014*	
3	rs2605393	STAC	36384605	G	T	40	83	1.06E-04	58.52	118.88	0.49	5.86E-06	0,13*	
19	rs2664156	KLK2 KLK3 KLKP1 KLK4 KLK15	56068975	C	T	48	16	6.33E-05	91.96	40.01	2.30	6.13E-06	0,34*	Prostate cancer
16	rs195656	HYDIN	69604985	A	G	18	34	2.65E-02	29.79	76.09	0.39	6.83E-06	0,52*	
11	rs4930144	IGF2AS TH MRPL23 TNNT3 SYT8 ASCL2 TNNI2LSP1 IGF2 INS-IGF2 INS H19	2005064	A	G	62	32	1.97E-03	116.06	57.04	2.03	7.28E-06	0,38*	Prostate cancer/Type 1 diabetes
4	rs17029173	RG9MTD2 C4orf17 MTTP	100728344	G	T	27	70	1.27E-05	27.06	71.49	0.38	7.65E-06	*0,0069* *	
4	rs13128441	STK32B	5213290	C	T	32	77	1.63E-05	32	79	0.41	8.16E-06	0,74*	Coronary heart disease
3	rs1871350	STAC	36348769	C	T	27	69	1.81E-05	27.06	71.19	0.38	8.49E-06	0,13*	
3	rs2046000	STAC	36327368	A	C	28	72	1.08E-05	28.05	72.46	0.39	9.42E-06	0,13*	
17	rs7209752	CCDC144C LOC284194 SPECC1 AKAP10	19909989	A	G	9	26	4.06E-03	12.61	46.46	0.27	1.06E-05	0,75*	
1	rs3795277	KIAA1751 PRKCZ GABRD	1970978	A	C	20	8	2.33E-02	46.62	12.76	3.65	1.11E-05	0,52*	Reasoning/height
2	rs10203748	TGFBRAP1 C2orf49 NCK2 FHL2 GPR45	105442542	C	T	20	43	3.76E-03	23.93	65.35	0.37	1.17E-05	0,013*	AIDS
11	rs318966	NTM	130871348	A	G	15	42	3.49E-04	33.86	80.79	0.42	1.17E-05	0,015*	Asperger disorder
6	rs9402234	TMEM200A SAMD3	130869175	C	T	17	7	4.12E-02	49.86	14.87	3.35	1.37E-05	0,38*	height
9	rs1536689	C9orf93 BCN2^a^	16119630	A	G	42	18	1.95E-03	97.95	46.04	2.13	1.52E-05	0,097*	HbA1c/glucose lecvels
4	rs6838036	DC2 AGXT2L1 RPL34	109630528	A	C	106	57	1.24E-04	127.72	67.47	1.89	1.62E-05	*0,0048* *	
3	rs17283813	LPP	190122389	A	G	11	26	1.37E-02	17.51	53.96	0.32	1.62E-05	0,082*	***IgE/vitiligo/Celiac***
3	rs1871352	STAC	36329541	A	C	27	69	1.81E-05	27.99	70.79	0.40	1.66E-05	0,13*	
1	rs12747934	FOXD3	63540185	A	G	20	57	2.48E-05	24.88	65.59	0.38	1.87E-05	0,68*	
1	rs4323662	LOC100288079 IVNS1ABP	183697117	G	T	41	22	1.67E-02	98.41	46.85	2.10	1.89E-05	0,032*	
3	rs1842149	STAC	36366714	G	T	51	17	3.74E-05	61.29	22.22	2.76	1.91E-05	0,13*	
19	rs3814892	PALM HCN2 C19orf21 POLRMT FSTL3 PRSSL1 RNF126 FGF22	589853	A	G	11	31	2.03E-03	23.45	63.03	0.37	2.08E-05	0,88*	
3	rs12631757	THRB	24618577	C	T	38	15	1.58E-03	80.09	34.53	2.32	2.09E-05	0,22*	Hematol. and biochem. traits
10	rs7097380	SORCS1	108671659	A	G	118	60	1.38E-05	119	62	1.92	2.27E-05	0,20*	HbA1c/glucose lecvels
1	rs12734001	PPP1R12B SYT2 UBE2T	200657537	C	T	75	40	1.10E-03	129.09	69.46	1.86	2.32E-05	0,012*	
10	rs17094083	GFRA1	117850841	C	T	27	68	2.59E-05	30.84	74.17	0.42	2.35E-05	0,27*	
3	rs12632771	CX3CR1	39223856	A	G	36	8	2.43E-05	36	8	4.50	2.43E-05	0,21*	

The top 35 associated SNPs are listed together with the surrounding genes defined by either Grail (www.broadinstitute.org/mpg/grail/) or the Genome Browser (http://genome.ucsc.edu/). The disease associations are acquired from the “Catalog of Published Genome-Wide Association Studies” (http://www.genome.gov/gwastudies/).

For PLINK: genotypes were imputed if any of the posterior probabilities were >0.95.

For expTDT: T and U are the expected transmission counts (based on all the posterior imputation probabilities).

NPL – the most significant Non Parametric Linkage (NPL) p-value for the same locus as the SNP. P-values below 0.05 are marked in italics.

T and U – the number of heterozygous parents who transmit the alleles A1 and A2, respectively. T/U – transmission odds based on the expected transmission counts.

* the marker in the set of SNPs from the linkage analysis closest to the marker in the SNP column (when this marker was not run in the linkage analysis).

a closest known gene. located >500 kb from associated SNP.

### HLA Stratified Transmission Disequilibrium Test (TDT)

In Figure 1b and Table 2, we present results from the TDT analysis stratified on the *HLA-DQ* risk factor. For this analysis 115 affected offspring trios were included in the “low-risk” group and 88 trios were put in the “high-risk” group. A region including the *DUSP10* gene (also known as *MKP5*) reached genome-wide significance (p-value = 3.8×10^−8^) in the low-risk group. Figure 1c presents this region including the most associated SNPs plotted on the x-axis using SNAP.

**Table 2:** HLA stratified Transmission Disequilibrium Test (TDT).

						High Risk					Low risk						All										
_Chr_	_SNP_	_gene(s)_	_BP_	_A1_	_A2_	_T_	_U_	_T/U_	_chisq_	_p-value_	_T_	_U_	_T/U_	_chisq_	_p-value_	_weighted chisq_	_T_		_U_		_T/U_		_chisq_		_P-value_		
*1*	*rs12144971*	*DUSP10*	*220099108*	*C*	*T*	*26*	*35*	*0.74*	*1.33*	*2.49E-01*	*87*	*28*	*3.11*	*30.27*	*3.76E-08*	*20.24*	*116*	*64*		*1.81*		*15.02*		*1.06E-04*			
*1*	*rs4240931*	*DUSP10*	*220105678*	*T*	*C*	*26*	*35*	*0.74*	*1.33*	*2.49E-01*	*87*	*28*	*3.11*	*30.27*	*3.76E-08*	*20.24*	*116*	*64*		*1.81*		*15.02*		*1.06E-04*			
10	rs1247697	SVIL	29901347	C	A	41	35	1.17	0.47	4.91E-01	22	69	0.32	24.27	8.35E-07	13.44	63	104		0.61		10.07		1.51E-03			
1	rs4846734	DUSP10	220139621	G	A	20	25	0.80	0.55	4.56E-01	53	15	3.53	21.24	4.06E-06	13.00	76	41		1.85		10.47		1.21E-03			
10	rs7097380	SORCS1	108671659	A	G	46	34	1.35	1.8	1.80E-01	72	26	2.77	21.59	3.37E-06	12.70	118	60		1.97		18.90		1.38E-05			
2	rs6755308	PRKCE	46083771	A	G	11	33	0.33	11	9.11E-04	32	8	4.00	14.40	1.48E-04	12.62	43	41		1.05		0.05		8.27E-01			
1	rs11811613	DUSP10	220122026	G	A	19	25	0.76	0.82	3.66E-01	54	17	3.18	19.28	1.13E-05	12.22	76	43		1.77		9.15		2.49E-03			
2	rs13017044	PRKCE	46086853	A	G	12	39	0.31	14.29	1.56E-04	43	18	2.39	10.25	1.37E-03	12.09	55	57		0.96		0.04		8.50E-01			
10	rs11193120	SORCS1	108678768	G	A	50	35	1.43	2.64	1.04E-01	70	26	2.69	20.17	7.10E-06	11.94	120	61		1.97		19.23		1.16E-05			
1	rs11102146	KCNA3	111007559	C	T	13	17	0.76	0.53	4.65E-01	48	15	3.20	17.29	3.22E-05	11.88	61	34		1.79		7.67		5.60E-03			
10	rs4748417	STAM TMEM236	17819812	T	C	2	0	NA	2	1.57E-01	18	2	9.00	12.80	3.47E-04	11.82	20	2		10.0		14.73		1.24E-04			
2	rs4972810	DLX1 DLX2 PDK1 MAP1D ITGA6	172926135	A	G	15	11	1.36	0.61	4.33E-01	41	11	3.73	17.31	3.18E-05	11.74	56	22		2.55		14.82		1.18E-04			
3	rs1871352	STAC	36329541	A	C	15	25	0.60	2.5	1.14E-01	12	44	0.27	18.29	1.90E-05	11.71	27	69		0.39		18.38		1.81E-05			
3	rs1871350	STAC	36348769	C	T	15	25	0.60	2.5	1.14E-01	12	44	0.27	18.29	1.90E-05	11.71	27	69		0.39		18.38		1.81E-05			
10	rs10884387	SORCS1	108682142	T	C	43	33	1.30	1.32	2.51E-01	71	27	2.63	19.76	8.80E-06	11.70	114	60		1.90		16.76		4.25E-05			
3	rs2046000	STAC	36327368	A	C	15	27	0.56	3.43	6.41E-02	13	45	0.29	17.66	2.65E-05	11.68	28	72		0.39		19.36		1.08E-05			
1	rs12734338	PPP1R12B SYT2 UBE2T	200736346	C	T	20	36	0.56	4.57	3.25E-02	18	53	0.34	17.25	3.27E-05	11.66	39	90		0.43		20.16		7.11E-06			
22	rs1296826	BID BCL2L13 SLC25A18 ATP6V1E1	16459518	C	T	3	9	0.33	3	8.33E-02	3	23	0.13	15.38	8.77E-05	11.47	6	32		0.19		17.79		2.47E-05			
4	rs7687176	INTS12 GSTCD	106746555	T	C	10	7	1.43	0.53	4.67E-01	2	24	0.08	18.62	1.60E-05	11.47	12	31		0.39		8.40		3.76E-03			
2	rs4972809	DLX1 DLX2 PDK1 MAP1D ITGA6	172925337	A	G	16	12	1.33	0.57	4.50E-01	41	11	3.73	17.31	3.18E-05	11.45	57	23		2.48		14.45		1.44E-04			
6	rs13207543	ELOVL4 SH3BGRL2 TTK	80592601	A	C	13	26	0.50	4.33	3.74E-02	52	19	2.74	15.34	8.99E-05	11.44	65	45		1.44		3.64		5.65E-02			
4	rs1032355	RG9MTD2 C4orf17 MTTP	100758919	C	T	9	42	0.21	21.35	3.82E-06	24	37	0.65	2.77	9.60E-02	11.23	33	81		0.41		20.21		6.94E-06			
5	rs11952677	SCAMP1 LHFPL2	77823690	G	A	27	34	0.79	0.80	3.70E-01	55	17	3.24	20.06	7.52E-06	11.23	84	51		1.65		8.07		4.51E-03			
1	rs12743521	DUSP10	220167571	A	G	21	24	0.88	0.2	6.55E-01	50	15	3.33	18.85	1.42E-05	11.22	74	40		1.85		10.14		1.45E-03			
12	rs11068315	FBXW8 HRK TESC RNFT2	115994935	T	C	8	6	1.33	0.28	5.93E-01	4	26	0.15	16.13	5.90E-05	11.09	12	32		0.38		9.09		2.57E-03			
6	rs7745052	FBXL4 C6orf168 USP45 COQ3 POU3F2 SFRS18	99747331	A	G	54	17	3.18	19.28	1.13E-05	32	25	1.28	0.86	3.54E-01	11.08	86	42		2.05		15.12		1.01E-04			
12	rs17245501	PPFIA2	80260470	C	T	17	18	0.94	0.028	8.66E-01	45	13	3.46	17.66	2.65E-05	11.02	62	31		2.00		10.33		1.31E-03			
1	rs7544501	DUSP10	220168985	T	C	21	24	0.88	0.2	6.55E-01	51	16	3.19	18.28	1.90E-05	11.02	75	41		1.83		9.97		1.60E-03			
1	rs785627	LPHN2	82521165	T	C	30	17	1.77	3.60	5.79E-02	25	61	0.41	15.07	1.04E-04	11.02	56	79		0.71		3.92		4.78E-02			
8	rs720131	RAD21	117985196	A	G	35	40	0.88	0.33	5.64E-01	79	34	2.32	17.92	2.30E-05	10.90	115	75		1.53		8.42		3.71E-03			
12	rs11104365	MGAT4C	86164650	C	T	41	57	0.72	2.61	1.06E-01	34	79	0.43	17.92	2.30E-05	10.81	77	136		0.57		16.34		5.29E-05			
14	rs7144018	NAT12 EXOC5 C14orf108	56676820	C	T	29	3	9.67	21.12	4.30E-06	14	20	0.70	1.06	3.04E-01	10.79	45	23		1.96		7.12		7.63E-03			

The top 32 associated SNPs from the results of the HLA stratified analysis. Surrounding genes are defined by either Grail (www.broadinstitute.org/mpg/grail/) or the Genome Browser (http://genome.ucsc.edu/). The low risk group consists of 115 trios and the high risk group of 88 trios. Genotypes were imputed if any of the posterior probabilities were >0.95.

Chisq – the value of the TDT test statistic for the transmission counts.

Weighted Chisq – based on the transmission counts of the low and high-risk groups. This value is used for ranking the results.

T and U – the number of heterozygous parents who transmit the alleles A1 and A2, respectively. T/U – transmission odds based on the expected transmission counts.

### Interaction Analyses

**Figure 2:**
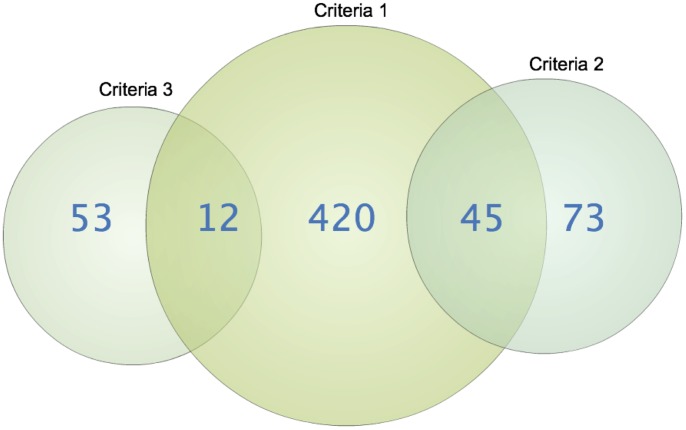
Illustration of the three inclusion criteria used for pathway and interaction analyses. *The first criteria* of p-values less than 3.0×10^−4^ in the linkage TDT analysis resulted in a total of 477 markers. *The second criteria* included a comparison of the results from this study with the results from the study by Dubois et al. [3]. We included 118 SNPs that had a simple score based on a combined p-value less than 5.0×10^−5^ and in the same allelic direction in both datasets. *The third criteria* involved selecting markers with a large effect size. We included 65 markers which had a ratio of transmitted versus not transmitted (T/NT) alleles of over 5 or below 0.2, combined with a p-value of less than 2.0×10^−3^.

Since some markers just below genome-wide significance are still expected to be true findings, we wanted to try and separate these from the, in fact, true negative findings (those that show linkage and association close to genome-wide significance just by chance). In total, 603 SNP markers from 383 independent regions and their surrounding genes were identified by three inclusion criteria (Fig. 2 and Table S1). These genes were subsequently used for pathway and two-locus interaction analyses.

#### Two-locus interaction analysis

Two-locus interaction analysis, identified 582 SNP pairs with a p-value of less than 1.0×10^−4^ for the test comparing the model M_0_ of no association and the general two-locus model M_G_. Out of these, 101 pairs from 87 regions deviated significantly (p<0.05) from a purely multiplicative model (M_M_), which is the best fitting model when at least one of the SNP markers is false. Under the null hypothesis we expect to find 29 such pairs. The 101 pairs showed either epistasis (individuals carry both risk alleles) or evidence of heterogeneity (individuals carry either the one or the other risk allele from the two loci).

The results with a p-value <1.0×10^−4^ for epistasis and those with high p-value (>0.05), which represent pairs that did not show convincing deviation from the heterogeneity model are listed in Table 3 and 4. Several loci were in an epistatic relationship with HLA; rs4899272 (*ACTN1*), rs1073933 (*COX7C*), rs10482751 (*TGFB2*), rs571879 (*APPL1*) and rs7590305 (*FABP1*). Also, previously identified susceptibility loci for CD were involved in several interactions: rs4899272 (*ACTN1*), rs6741418 (*STAT1, GLS*), rs13096142 (*CCR1,2,3,5*), rs10197319 (*ICOS, CTLA4*) and rs870875 (*CD247*).

**Table 3:** The top epistasis interaction results from the 101 two-locus interaction analysis.

*Snp 1*	*Genes*	chr	*Snp 2*	*Genes*	chr	*N*	*P_02_*	*P_12_*	*P_M2_*
rs2187668	HLADQ	6	*rs4899272*	*ACTN1*	14	*95*	*4.0E-17*	*1.42E-13*	*4.E-02*
			*rs204034*	*SHISA9*	16	*94*	*1.3E-14*	*1.09E-12*	*5.E-02*
			rs571879	APPL1 HESX1 IL17RD. DNHD2. ASB14	3	94	2.3E-15	5.21E-11	3.E-02
rs204999	HLA	6	rs1073933	COX7C	5	94	9.9E-14	9.27E-12	3.E-02
			rs11836636	ATXN7L3B KCNC2	12	*91*	*1.1E-12*	*8.15E-11*	*4.E-02*
rs7745052	FBXL4. C6orf168. USP45. COQ3. POU3F2. SFRS18	6				92	2.3E-05	1.79E-05	4.E-02
rs10749738	FOXD3	1	rs1373649	BMPR1B	4	93	2.7E-05	1.78E-05	4.E-02
rs3860295	RASSF5 IKBKE	1	rs13096142	CCR5 CCR3 LTF CCR2 CCR1	3	95	1.1E-05	6.48E-06	1.E-02
rs9396802	KIF13A NUP153 FAM8A1	6	rs2194633	NETO1	18	95	3.8E-06	6.82E-06	2.E-02
rs9296204	MTCH1 PI16	6	rs4385459	LY96 JPH1 GDAP1 TMEM70 TCEB1	8	95	2.8E-05	9.91E-06	3.E-02
rs9397928	ARID1B*	6	rs2415836	FSCB*	14	93	2.8E-05	1.75E-05	3.E-03
rs1145212	APOA5 ZNF259 BUD13	11	rs10083673	MYO5A	15	95	6.6E-05	1.77E-05	2.E-03
rs7756191	DNAH8	6	rs1108001	NAV2 HTATIP2 DBX1 PRMT3	11	95	3.5E-05	2.60E-05	3.E-03
rs10197319	ICOS CTLA4	2	rs882820	SRL TFAP4	16	94	1.4E-05	3.03E-05	3.E-05
rs4899272	ACTN1	14	rs17703807	C15orf41	15	83	2.9E-05	8.68E-05	1.E-02

All SNP pairs which reached an interaction p-value of P_12_<1.0×10^−4^, in addition to P_M2_<0.05.

* closest known gene. located >500 kb from associated SNP.

P_02_– p-value for the test statistic comparing the models M_0_ (no association) and the general model M_G_.

P_12_– p-value for the test test comparing the models M_R_ (heterogeneity) and the general model M_G_.

P_M2_– p-value for the test comparing the models M_M_ (multiplicative) and the general model M_G_.

**Table 4:** The top heterogeneity results from the 101 two-locus interaction analysis.

*SNP1*	*Genes*	*chr*	*SNP2*	*Genes*	*chr*	*N*	*P_02_*	*P_12_*	*PM2*
rs4899272	ACTN1	14	rs4820682	SRRD HPS4 TFIP11 ASPHD2 MIR548JTPST2 CRYBB1 CRYBA4	22	95	7.1E-06	6.97E-02	2.E-02
			rs4426448	DOK6	18	94	9.5E-06	6.80E-01	1.E-02
			rs870875	CD247	1	94	9.4E-05	7.19E-02	3.E-02
			rs4842007	PAEP	9	95	8.6E-06	5.66E-01	4.E-02
rs571879	APPL1 HESX1 IL17RDDNHD2 ASB14	3	rs4385459	LY96 JPH1 GDAP1 TMEM70 TCEB1	8	94	4.1E-05	5.81E-01	5.E-02
rs7590305	FABP1 THNSL2	2	rs390495	MICAL3	22	93	7.0E-05	9.09E-01	3.E-03
rs7745052	FBXL4 C6orf168 USP45COQ3 POU3F2 SFRS18	6	rs4930144	IGF2AS TH MRPL23 TNNT3 SYT8 ASCL2TNNI2 LSP1 IGF2 INS-IGF2 INS H19	11	50	1.9E-05	5.30E-01	3.E-02
rs10749738	FOXD3	1	rs10498982	EPHA7*	6	93	2.0E-05	1.95E-01	4.E-02
			rs2605393	STAC	3	63	7.3E-05	4.37E-01	4.E-02
rs2187668	HLADQ	6	rs11013804	KIAA1217	10	94	3.5E-14	8.40E-02	2.E-02
			rs1676235	ESRRB ANGEL1 VASH1	14	43	2.0E-07	8.55E-02	3.E-02
rs958802	KANK4 L1TD1 INADL	1	rs2194633	NETO1	18	95	1.9E-05	5.55E-01	3.E-02
rs2345981	KHDRBS2	6	rs6495130	RYR3	15	94	6.1E-05	1.58E-01	3.E-02
rs11940562	PCDH7*	4	rs4905043	ITPK1 CHGA	14	44	4.6E-05	2.77E-01	2.E-02
rs4656538	POU2F1	1	rs2187668	HLADQ	6	94	3.0E-13	1.19E-01	5.E-02
rs3860295	RASSF5 IKBKE	1	rs7046385	SMC2	9	94	5.3E-05	1.07E-01	2.E-02
rs6741418	STAT1 GLS STAT4	2	rs10798004	C1orf25 C1orf26 IVNS1ABP RNF2	1	87	7.2E-05	7.68E-02	4.E-02
			rs1571812	VLDLR	9	86	3.0E-05	9.19E-02	4.E-02
			rs882820	SRL TFAP4	16	87	4.2E-05	3.52E-01	6.E-03
			rs1470379	VIM	10	82	1.0E-05	3.70E-01	8.E-03
			rs10946659	DCDC2 NRSN1	6	87	1.9E-06	6.64E-01	9.E-03
rs10482751	TGFB2	1	rs1571812	VLDLR	9	92	5.2E-05	1.86E-01	1.E-02

All SNP pairs which reached an interaction p-value of P_12_>0.05, in addition to P_M2_<0.05.

* closest known gene located >500 kb from associated SNP.

P_02_– p-value for the test statistic comparing the models M_0_ (no association) and the general model M_G_.

P_12_– p-value for the test test comparing the models M_R_ (heterogeneity) and the general model M_G_.

P_M2_– p-value for the test comparing the models M_M_ (multiplicative) and the general model M_G_.

#### Pathway analysis

**Figure 3:**
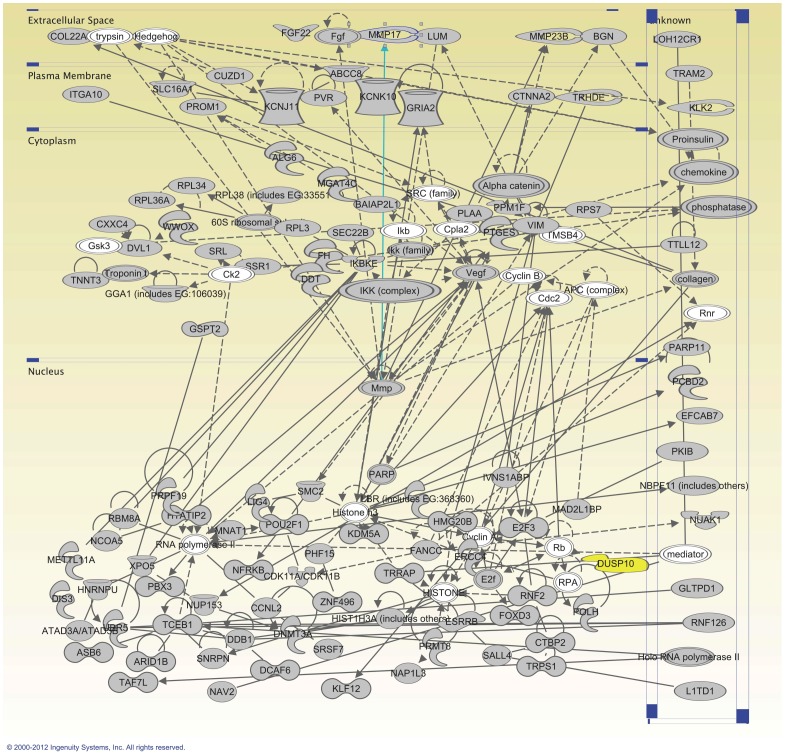
Ingenuity network 1. The top network identified by the Ingenuity IPA software using genes surrounding all 603 most associated SNPs from the TDT analysis. Molecules in gray were present among the genes from our TDT analysis and molecules in white were added by the IPA software. The *DUSP10* gene is marked in yellow.

Biological functions clustered by Ingenuity Pathway Analysis (IPA) and Genetrail [13] are shown in Table 5, 6 and 7. Several clusters were significant after correction for multiple comparisons. The most significant network implicated by IPA included *DUSP10* (Fig. 3 and Table 8). The second top network included the MHC complex (HLA) and the third top network included *LPP,* which is located within the most significantly non-HLA associated region identified in CD so far [3].

**Table 5:** Biological functions of genes surrounding the 603 top associated SNPs. Results from IPA.

FunctionAnnotation	p-value(Raw)	B-H p-value*	Molecules	Molecules
non-insulin-dependent diabetes mellitus	0.0000057	0.025	ABCC8. ADRA1B. ADRA1D. AGT. APOA5. ATP10A. BCL2L11. CCR5. CD38. CNTNAP2. FOXP1. FTO. HFE. HFE2. INS. INSR. KCNJ11. KIRREL3. KLF10. mir-154. mir-448. MTTP. PBX3. PIEZO2. PPARA. PPP3CA. PRDM10. RGS5. VEGFA. ZMYM2	30
quantity of metal	0.0000082	0.025	ABCC8. ADRA1B. AGT. APLP2. ATP2B3. BCL2. BMP2. BTK. CAMLG. CCR5. CD247. CD38. CHGA. CX3CR1. CXCL13. DARC. DCN. DVL1. EGF (includes EG:13645). FBXL5. FCER1A. GNA14. GNB1. HFE. HFE2. IGF2. INS. INSR. KCNJ11. LTF. NTS. NUCB2. POMC. PRL. PRNP. PTGDR2. RGS1. RYR3. SELL. SOD1. TRPM8. TXNIP. VAV3. VEGFA	44
incorporation of thymidine	0.000010	0.025	AGT. AKAP13. BMP2. CD40. EGF (includes EG:13645). IGF2. INS. INSR. PRL. THBS2. TNFSF13B. VEGFA. WT1	13
quantity of Ca2+	0.000018	0.033	ABCC8. ADRA1B. AGT. ATP2B3. BCL2. BTK. CAMLG. CCR5. CD247. CD38. CHGA. CX3CR1. CXCL13. DARC. DCN. DVL1. EGF (includes EG:13645). FCER1A. GNA14. GNB1. IGF2. INS. INSR. KCNJ11. NTS. NUCB2. POMC. PRL. PRNP. PTGDR2. RGS1. RYR3. SELL. SOD1. TRPM8. VAV3. VEGFA	37
eye development	0.000022	0.033	BID. BMPR1B. CD247. CHD7. CRYBB2. CX3CR1. DLX1. DNMT3A. EBF3. EGF (includes EG:13645). FJX1. FTO. GJA3. H19. HESX1. IFT88. IGF2. IRX3. ITGA6. LUM. MITF. OGN. PAX5. PROM1. PRRX2. PYGO1. SEMA5A. SOD1. STAT1. TGFB2. TH. THBS2. THRB. TUB. USH2A. VEGFA. WT1	37
diabetes mellitus	0.000027	0.034	ABCC8. ABCG1. ABT1. ADRA1B. ADRA1D. AGT. APOA5. ATP10A. BCL2. BCL2L11. BTC. BTN2A1. BTN3A2. CBLB. CCR5. CD200. CD38. CD40. CNTNAP2. CYBA. E2F3. ENAH. FOXP1. FTO. GABRD. HFE. HFE2. HIST1H3A (includes others). HTR2C. ICOS. IGF2-AS1. INS. INSR. KCNJ11. KIRREL3. KLF10. mir-154. mir-448. MTTP. PBX3. PDE8A. PGM1. PIEZO2. PPARA. PPP3CA. PRDM10. PRSS16. PRUNE2. PXDNL. RGS1. RGS5. SELL. SOD1. TH. THRB. TSPO. VEGFA. ZMYM2	58
angiogenesis of bone	0.000032	0.034	BMP2. NOG. TGFB2. VEGFA	4
quantity of metal ion	0.000071	0.043	ABCC8. ADRA1B. AGT. ATP2B3. BCL2. BTK. CAMLG. CCR5. CD247. CD38. CHGA. CX3CR1. CXCL13. DARC. DCN. DVL1. EGF (includes EG:13645). FCER1A. GNA14. GNB1. IGF2. INS. INSR. KCNJ11. NTS. NUCB2. POMC. PRL. PRNP. PTGDR2. RGS1. RYR3. SELL. SOD1. TRPM8. TXNIP. VAV3. VEGFA	38
development of head	0.000069	0.043	BCL2. BCL2L11. BID. BMP2. BMPR1B. CD247. CHD7. CRYBB2. CX3CR1. DLX1. DNMT3A. EBF3. EGF (includes EG:13645). FJX1. FTO. GJA3. H19. HESX1. IFT88. IGF2. IRX3. ITGA6. LUM. MITF. MYO5A. NOG. OGN. PAX5. PROM1. PRRX2. PYGO1. SEMA5A. SOD1. STAT1. TGFB2. TH. THBS2. THRB. TUB. USH2A. VEGFA. WT1	42
migration of cells	0.000057	0.043	ADI1 (includes EG:104923). AGT. APLP2. APPL1. ARHGAP5. B3GAT1. BCL2. BGN. BID. BMP2. BTC. BTK. CBLB. CCR5. CD200. CD247. CD36. CD38. CD40. CD99. CHGA. CMA1. CNTNAP2. CSF2RA. CTBP2. CTNNA2. CTSG. CX3CR1. CXCL13. DARC. DCDC2. DCN. DISC1. DLX1. DYX1C1. E2F3. EBF3. EGF (includes EG:13645). ELMO2. FCER1A. FH. FHL2. GFRA1. GNA12. GRIA2. GZMB. HTATIP2. ICOS. IGF2. INS. INSR. ITGA6. KIAA0319. LAMA2. LPP. LSP1 (includes EG:16985). LTF. LUM. LY96 (includes EG:17087). MAPK1. MNX1. MTAP. MYO10. MYO1F. NAV1. NOG. NPTX2. NTS. NUCB2. PAEP. PDPN (includes EG:10630). PEX11B. PEX13. POMC. POU3F2. PPARA. PPM1F. PRKCQ. PRKCZ. PRL. PRNP. PROK2. PTGDR2. PTGES. PTK2 (includes EG:14083). PVR. RASSF5. RGS1. SATB2. SELL. SEMA5A. SOD1. STAT1. TDP2. TGFB2. THBS2. TIAM1. TNFAIP8. TNFRSF18. TNFRSF4. TNFSF13B. TSPO. UNC5C. VASH1. VAV3. VEGFA. VIM. WWOX	108
cell movement	0.000073	0.043	ADCY10. ADI1 (includes EG:104923). AGT. APLP2. APPL1. ARHGAP5. B3GAT1. BCL2. BGN. BID. BMP2. BTC. BTK. CATSPER3. CBLB. CCR5. CD200. CD247. CD36. CD38. CD40. CD99. CHGA. CMA1. CNTNAP2. CSF2RA. CTBP2. CTNNA2. CTSG. CX3CR1. CXCL13. DARC. DCDC2. DCN. DISC1. DLX1. DYX1C1. E2F3. EBF3. EGF (includes EG:13645). ELMO2. ENAH. FCER1A. FH. FHL2. GFRA1. GNA12. GNB1. GRIA2. GZMB. HTATIP2. ICOS. IFT88. IGF2. INS. INSR. ITGA6. KIAA0319. LAMA2. LPP. LSP1 (includes EG:16985). LTF. LUM. LY96 (includes EG:17087). MAPK1. MNX1. MTAP. MYO10. MYO1F. NAV1. NCK2. NOG. NPTX2. NTS. NUCB2. PAEP. PDPN (includes EG:10630). PEX11B. PEX13. POMC. POU3F2. PPARA. PPM1F. PRKCQ. PRKCZ. PRL. PRNP. PROK2. PTGDR2. PTGES. PTK2 (includes EG:14083). PVR. RASSF5. RGS1. RGS10. SATB2. SELL. SEMA5A. SOD1. SPAG16. STAT1. TAS1R3. TDP2. TGFB2. THBS2. THRB. TIAM1. TNFAIP8. TNFRSF18. TNFRSF4. TNFSF13B. TSPO. UNC5C. VASH1. VAV3. VEGFA. VIM. WWOX	118
apoptosis	0.000069	0.043	ABCG1. ADCY10. ADI1 (includes EG:104923). ADRA1B. ADRA1D. AGPAT2. AGT. APPL1. ATXN1. BCL2. BCL2L11. BCL2L13. BGN. BID. BIK. BMP2. BMPR1B. BTC. BTK. CACNA1A. CBLB. CCDC86. CCNI. CCNL2. CCR5. CD200. CD247. CD36. CD38. CD40. CD5L. CD99. CDK11A/CDK11B. CSF2RA. CTBP2. CTSG. CX3CR1. CYBA. DACH1. DCN. DLX1. DNMT3A. DUSP10. DVL1. E2F3. EGF (includes EG:13645). EPHA7. EPHX1. EPM2A. FABP1. FANCC. FBXL5. FCER1A. FHL2. FOXP1. FSTL3. GFRA1. GNA12. GRIA2. GZMB. HFE. HSF2. HTATIP2. ICOS. IFNE. IGF2. IKBKE. IL17RD. INS. INSR. IPPK. ITGA6. ITGB3BP. ITPK1. IVNS1ABP. KIFAP3. KLF10. LAMA2. LIG4. LSP1 (includes EG:16985). LTF. LUM. MAGED1. MAGEH1. MAPK1. mir-154. mir-506. MITF. MLLT3. MNAT1. MTCH1. NELL1. NOG. NPTX2. NTS. PAEP. PAWR. PAX5. PDCD6IP. PEX11B. PKN2. POLH. POMC. PPARA. PPM1F. PPP2R4. PPP3CA. PRAME. PRKCH. PRKCQ. PRKCZ. PRL. PRNP. PRPF19. PRUNE2. PTGES. PTK2 (includes EG:14083). PUS10. RASSF5. RGS5. RNASEH1. SELL. SGCG. SLC25A6. SMARCA2. SMOX. SOD1. SPAG16. ST14. STAT1. TFAP4. TGFB2. THBS2. TIAM1. TMEM109. TMEM132A. TNFAIP8. TNFRSF18. TNFRSF4. TNFSF13B. TREX2. TRPS1. TSPO. TUB. TXNIP. UNC5C. VEGFA. VIM. VPS13A. WT1. WWOX. XPO1. ZMYM2	153
quantity of leukocytes	0.000076	0.043	AGT. BCL2. BCL2L11. BID. BIK. BST1 (includes EG:12182). BTK. CARD11. CBLB. CCR5. CD200. CD247. CD36. CD38. CD40. CD5L. CRLF2. CX3CR1. CXCL13. DCN. DMD. DUSP10. FABP1. FANCC. FCER1A. FOXP1. GNA12. HESX1. ICOS. IGF2. INS. ITGA6. KDM5A. KIFAP3. KLF10. LAMA2. LIG4. LSP1 (includes EG:16985). LUM. NOG. PAWR. PAX5. PRKCQ. PRL. PRNP. PROK2. PTGDR2. PTGES. PTK2 (includes EG:14083). RASSF5. RGS1. RGS10. SELL. SOD1. ST14. STAM. STAT1. TNFRSF4. TNFSF13B. TOX. TXNIP. VAV3. VEGFA. VPREB1. WWOX	65

A total of 823 genes surrounding the 603 top associated SNPs were put into the IPA software.

Surrounding genes were defined by either Grail (www.broadinstitute.org/mpg/grail/) or the Genome Browser (http://genome.ucsc.edu/). Gene families located in the same region were manually curated so that only one gene in each family remained in each region, based on a similar official gene symbol.

* Hochberg Y, Benjamini Y. Statistics in medicine 1990; 9∶811–8.

**Table 6:** Biological functions of genes surrounding the 603 top associated SNPs. Results from GeneTrail.

Category	rank	Subcategory	expected	observed	p-value (raw)	Genes
						
KEGG	1	Type II diabetes mellitus	1.91	7	0.0026	ABCC8 CACNA1A INS INSR KCNJ11 MAPK1 PRKCZ
KEGG	2	Salivary secretion	3.62	9	0.003	ADRA1B ADRA1D AMY1B ATP2B3 BST1 CALML6 CD38 CST2 RYR3
KEGG	3	Pathways in cancer	13.35	23	0.007	APPL1 BCL2 BID BMP2 CBLB CSF2RA CTBP2 CTNNA2 DVL1 E2F3 EGF FGF22 FH ITGA6 LAMA2 MAPK1 MITF PTK2 RASSF5 STAT1 TCEB1 TGFB2 VEGFA
KEGG	4	T cell receptor signaling pathway	4.40	10	0.012	CARD11 CBLB CD247 ICOS MAPK1 NCK2 PDK1 PPP3CA PRKCQ VAV3
KEGG	5	TGF-beta signaling pathway	3.46	8	0.022	BMP2 BMPR1B DCN ID4 MAPK1 NOG TGFB2 THBS2
KEGG	6	Cytokine-cytokine receptor interaction	10.79	18	0.022	BMP2 BMPR1B CCR5 CD40 CRLF2 CSF2RA CX3CR1 CXCL13 EGF IFNA6 IFNE IL3RA PRL TGFB2 TNFRSF18 TNFRSF4 TNFSF13B VEGFA
KEGG	7	Arrhythmogenic right ventricular cardiomyopathy	3.09	7	0.034	ACTN1 CTNNA2 DMD ITGA10 ITGA6 LAMA2 SGCG
						
Gene Ontology	1	negative regulation of phosphatase activity	0.21	3	0.0006	PPP2R4 TGFB2 TIPRL
Gene Ontology	2	positive regulation of apoptosis	13.93	27	0.0008	AGT AKAP13 ARHGEF18 BCL2 BCL2L11 BCL2L13 BID BIK BMP2 BTK CD38 HTATIP2 IKBKE ITGB3BP MAGED1 MAPK1 MTCH1 PAWR PPP2R4 PRUNE2 PVR SOD1 TFAP4 TGFB2 TIAM1 VAV3 WT1
Gene Ontology	3	regulation of phosphatase activity	0.53	4	0.0015	BMP2 PPP2R4 TGFB2 TIPRL
Gene Ontology	5	glomerular epithelium development	0.08	2	0.0017	BASP1 WT1
Gene Ontology	6	vesicle	12.50	24	0.0017	APPL1 BGN CD36 CTSG CUZD1 CXXC4 CYBA DVL1 EGF GRIA2 HFE HPS4 LTF NRSN1 PALM RASSF9 SEC24A SOD1 SYT1 SYT2 TGFB2 TH THBS2 VEGFA
Gene Ontology	7	cellular defense response	2.38	8	0.0024	CCR5 CD300C CD5L CX3CR1 DCDC2 LSP1 LY96 NCR2
Gene Ontology	8	cytoplasmic vesicle	12.17	23	0.0026	BGN CD36 CTSG CUZD1 CXXC4 CYBA DVL1 EGF GRIA2 HFE HPS4 LTF NRSN1 PALM RASSF9 SEC24A SOD1 SYT1 SYT2 TGFB2 TH THBS2 VEGFA
Gene Ontology	9	phosphoinositide 3-kinase cascade	0.33	3	0.0033	AGT INS TGFB2
Gene Ontology	10	hindbrain development	0.37	3	0.0048	CTNNA2 MYO16 SDF4
Gene Ontology	11	regulation of neuronal synaptic plasticity	0.37	3	0.0048	NETO1 SHISA9 SYNGR1
Gene Ontology	12	neuron projection membrane	0.12	2	0.0049	CNTNAP2 SHISA9
Gene Ontology	13	dopamine biosynthetic process	0.12	2	0.0049	TGFB2 TH
Gene Ontology	14	hydrogen peroxide biosynthetic process	0.12	2	0.0049	CYBA SOD1
Gene Ontology	15	positive regulation of respiratory burst	0.12	2	0.0049	INS INSR
Gene Ontology	16	cardiac epithelial to mesenchymal transition	0.12	2	0.0049	BMP2 TGFB2
Gene Ontology	17	enzyme activator activity	7.11	15	0.0051	AGT APOA5 ARHGAP5 BCL2L13 BMP2 EGF MMP17 OPHN1 PITRM1 PPP1R12B PPP2R4 RGS1 RGS5 TBC1D15 VAV3
Gene Ontology	18	epidermal growth factor receptor signaling	0.74	4	0.0054	AGT EGF NCK2 SNX6
Gene Ontology	19	extracellular matrix	7.23	15	0.0060	ASPN BGN CMA1 CPXM2 CTSG DCN ECM2 LAMA2 LUM MMP23B OGN SOD1 TGFB2 USH2A VEGFA
						
NIA human disease	1	Diabetes Mellitus. Type 2	10.49	22	0.0003*	ABCC8 AGT AKAP10 APOA5 BTC CCR5 CD36 CMA1 CYBA FABP1 FTO INS INSR KCNJ11 MTTP PPARA PRKCZ SELL TH THBS2 TXNIP VEGFA
NIA human disease	2	Hyperlipoproteinemias	0.26	3	0.0012	APOA5 FABP1 PPARA
NIA human disease	3	Diabetic Angiopathies	1.94	7	0.0024	CD40 CYBA INS KCNJ11 PPARA TXNIP VEGFA
NIA human disease	4	Postmortem Changes	0.10	2	0.0026	DAOA TPH2
NIA human disease	5	Disease Progression	7.77	16	0.0030	AGT BCL2 CCR5 CD40 CMA1 CX3CR1 DCN EGF HFE KCNJ11 PPARA PRNP SELL SOD1 VEGFA WT1
NIA human disease	6	Birth Weight	1.69	6	0.0054	EGF EPHX1 FTO H19 INS TH
NIA human disease	7	Pathological Conditions. Signs and Symptoms	23.66	34	0.0073	AGT APOA5 BCL2 CCR5 CD40 CMA1 CX3CR1 CYBA DAOA DCN DISC1 DMD EGF EPHX1 FCER1A FTO H19 HFE HTR2C INS INSR KCNJ11 LTF MTTP PLXNA2 POMC PPARA PRNP SELL SOD1 TH TPH2 VEGFA WT1
NIA human disease	8	Bronchiolitis. Viral	0.15	2	0.0075	CCR5 CX3CR1
NIA human disease	9	Kidney Failure. Acute	0.15	2	0.0075	CYBA WT1
NIA human disease	10	Diseases in Twins	0.46	3	0.0086	DISC1 HFE PLXNA2
NIA human disease	11	Coronary Artery Disease	4.55	10	0.0127	AGT APOA5 CD36 CD40 CMA1 CX3CR1 CYBA PPARA THBS2 VEGFA
NIA human disease	12	Dyslexia	0.26	2	0.0233	DYX1C1 KIAA0319
NIA human disease	13	Myocardial Infarction	6.64	12	0.0282	AGT AKAP10 APOA5 CCR5 CTSG CX3CR1 HFE INSR MTTP THBS2 TNFRSF4 VEGFA
NIA human disease	14	Nutritional and Metabolic Diseases	18.45	26	0.0295	ABCC8 AGT AKAP10 APOA5 BTC CBLB CCR5 CD36 CMA1 CYBA DCN FABP1 FTO HTR2C INS INSR KCNJ11 MTTP POMC PPARA PRKCZ SELL TH THBS2 TXNIP VEGFA
NIA human disease	15	Overweight	0.31	2	0.0338	APOA5 FTO

A total of 823 genes surrounding the 603 top associated SNPs were put into the GeneTrail software. Surrounding genes were defined by either Grail (http://www.broadinstitute.org/mpg/grail/) or the Genome Browser (http://genome.ucsc.edu/). Gene families located in the same region were manually curated so that only one gene in each family remained in each region, based on a similar official gene symbol.

* Significant after multiple testing correction using FDR adjustment. (p_corr_-value=0.032) Size of test set: 823 (768 known). Number of known ref. IDs: 44829 Kegg: Number of annotated genes in test set was 220. Number of annotated genes in ref set was 5405. Gene Ontology: Number of annotated genes in test set was 476. Number of annotated genes in ref set was 11580.

NIA human genes sets: Number of annotated genes in test set was 76. Number of annotated genes in ref set was 1487

**Table 7:** Biological functions of genes surrounding SNPs from the two-locus interaction. Results from GeneTrail.

Category	rank	Subcategory	expected	observed	p-value(raw)	enrichment	Genes
KEGG	1	Amoebiasis	1.01	4	0.0178	up	ACTN1 CTSG GNA14 TGFB2
KEGG	2	T cell receptor signalling	1.04	4	0.0196	up	CBLB CD247 ICOS VAV3
KEGG	3	PPAR signaling pathway	0.66	3	0.0282	up	APOA5 CD36 FABP1
KEGG	5	Ubiquitin mediated proteolysis	1.34	4	0.0438	up	CBLB KLHL9 TCEB1 UBR5
KEGG	6	Primary immunodefciency	0.34	2	0.0441	up	BTK ICOS
KEGG	7	Basal transcription factors	0.35	2	0.0465	up	GTF2B TAF7L
							
NIA humandiseasegene sets	1	Hyperlipoproteinemias	0.07	2	0.0017	up	APOA5 FABP1
NIA humandiseasegene sets	2	Diabetes Mellitus Type 2	2.80	6	0.0493	up	APOA5 CCR5 CD36 CMA1 FABP1 TH

A total of 187 genes from the interaction analysis were put into the GeneTrail software.

Surrounding genes were defined by either Grail (www.broadinstitute.org/mpg/grail/) or the Genome Browser (http://genome.ucsc.edu/). Gene families located in the same region were manually curated so that only one gene in each family remained in each region, based on a similar official gene symbol.

Size of test set: 186 (173 known). Number of known ref. IDs: 44829.

KEGG: number of annotated genes in test set: 52. Genes in reference set: 5405.

NIA human disease gene sets: number of annotated genes in test set: 20. Genes in reference set: 1487.

**Table 8:** The top four networks generated by the Ingenuity IPA software (allowing only direct connections between proteins/genes).

1: Cell Morphology, Cellular Assembly and Organization, Hair and Skin Development and Function. Ingenuity Score: 155, 109 focus molecules.	ABT1,ACAP3,Ant,APLP2,APOA5,ARID1B,ASB6,ASPN,ATXN1,BASP1,BGN,BICD2,BIK,C1q,Cbp/p300,CCDC50,CCNL2,CD200,CD5L,CDK11A/CDK11B,CEP55,CHGA,COL22A1, collagen, **Collagen type I,Collagen type,IV**,Creb,CSF2RA,CXCL13,CYBA,CyclinA,CyclinE,DACH1,DCAF6,DCN,DDB1,DPP6,***DUSP10***,E2F3,E2f,EAPP,EFCAB7,ELOVL6, EPB41L2, FABP1,FCER1A,Fibrinogen,FOXD3,GGA1 (includesEG:106039),***GLS***,GLTPD1,GRIA2,GSPT2,GSTT2/GSTT2B,GTF2B,HESX1,HIST1H3A (includes others),Histone h3,Histone h4,HMG20B,HoloRNApolymeraseII,HTATIP2,IGF2,Immunoglobulin,INS,Insulin,ITGA6,ITGA10,ITGB3BP,IVNS1ABP,KDM5A,KLF12,**LAMA2,Lamin b, Laminin1,Laminin**,LBR (includes EG:368360),LIG4,LSAMP,LUM,**MMP17,Mmp,MMP23B**,MNAT1,MTTP,MVD,N-cor,NAP1L3,NBPF11, (includes others),NCOA5,**NFkB(complex),**NFRKB,NTS,PAX5,PBX3,PCBD2, PKIB,POU2F1, POU3F2,PPARA,PPM1F,PRL,PRMT3,PRMT8,PRNP,PTGES,PVR,pyruvate kinase, Rb,RBM8A,***RGS1***,RNA polymeraseII,RNF126,SLC25A6,SMC2,SRSF7,SYT8,TAF7L,TCEB1,TEAD4, **Tgf beta,TGFB2**,TGFBRAP1,THBS2,thymidine, kinase,thyroid hormonereceptor,TNFRSF4,TRHDE,TSPO,TXNIP,**Ubiquitin,**UBR5,Vegf,VEGFA,VIM,VPREB1,VPS37C,WT1,ZRANB1
2: Cell Signaling, Molecular Transport, Vitamin and Mineral Metabolism. Ingenuity score: 97, 86 focus molecules.	14-3-3,Actin,ADRA1B,ADRA1D,ADRBK2,AGT,AKAP13,Akt,alcohol group acceptor phosphotransferase, ***ACTN1***, Alpha tubulin,Angiotensin IIreceptor type 1,Ap1, ARHGAP5, ARHGEF18,BCL2,BCL2L11,BTK,Calmodulin,CaMKII,CARD11,caspase,CBLB,***CCR5*** **,**CD3,CD6,CD36,CD38,CD40,***CD247*** **,**chemokine,**CTSG**,CX3CR1,DARC, DISC1,DMD,DVL1,Dynamin, Dynein,dystroglycan,EGF (includesEG:13645),EPB41L3,EPHA7,ERK,ERK1/2,estrogen receptor,F Actin,FCRL4,FHL2,**Focal adhesion kinase**,Gprotein,Gap,GLRX3,**GNA12**,GNB1,Gpcr, GPR20,GPR27,GPR33,GPR45,GPR111,GPR115,GPR149,GPR161,growthfactor receptor,**GZMB**,Hsp70,Hsp90,HTR2C, ***ICOS***,Iga,INADL,INSR,JAK,Jnk,KLF10,LIN7A,LPHN2,LTF,MAPK1,Mapk,**MHC Class I (complex),** ***MHC Class II (complex***),MITF,Mlc,MRC1 (includes EG:100286774), MYT1L,NADK,NCK2,Nfat(family),NMDAReceptor,O3FAR1,OR51E2, ***P38 MAPK***, p85 (pik3r), Pak,PAWR,PDCD6IP,PDK1,PDPN (includes EG:10630),PI3K(complex),PI3K p85,Pkc(s),PKN2,PLC gamma,POMC,PP2A,**PPP3CA,PRKCH,PRKCQ,PRKCZ**,PTGDR2,**PTK2**(includes EG:14083),Rac,Ras,RASSF5,Rxr,SGCG, SMARCA2,SOD1,Spectrin,STAM,***STAT1*** *,*STAT,STAT5a/b,TAB1,TAS1R3,TCR,TH,THRB,TIAM1,TIMM8A,TNNT3,Troponin t, TUB,Tubulin, UTRN,VAV3,VAV,VN1R5
3: Cellular Assembly and Organization, Cellular Function and Maintenance, Developmental Disorder. Ingenuity score: 77, 69 focus molecules.	ABCG1,ABTB2,ACAD9,ACAD10,ACADSB,ADI1 (includesEG:104923),AGPAT2,AGPAT3,AGPAT4,AGPAT5,AGPAT6,ALDH18A1,ARL6,ATL2,B9D1,BAIAP2L1,BCL2L13, BRP44,C10orf35,C10orf88,CAMLG,CC2D2A,CCBL2,CCNI,CDH18,CDK5RAP2,CHIC2,CNTNAP2,CPSF3L,CRYL1,CTNNA2,DDX46,DMXL1,DOCK4,DSE,EBNA1BP2,ELMO2,ELMO3,ERGIC1,ERGIC2,ERGIC3,FAM125A,FAM125B,FAM175B,FBXO8,GEMIN6,GEMIN8,GLA,GNL1,HAUS2,HAUS3,HAUS5,HAUS7,HAUS8,HCN1,HCN2,HERC3,HERC4,HERC6,HFE,HNRNPH2,HNRPLL,IFT20,IFT52,IFT57,IFT88,IL13RA1,IL13RA2,INTS2,INTS9,***LPP*** *,*LSG1,MAD2L1BP,MAGEH1,MAOA,MCAT,MICAL3,MKS1 (includes EG:287612),MOAP1,MRPL23,MRPL20 (includes EG:39477),MRPL3 (includes EG:11222),MRPL40(includesEG:18100),MRPS21,MTAP,MYLIP,MYO1F,NDUFAB1, OSTF1,OXA1L, PALM,PCMTD1,PHKA1,PHRF1, PIGQ,PIGY,PPAP2B,PRAME,PTPN14,PYGB,REEP5,RIC3,RNF122,RNF166,RTN3,SARS2,SCYL3,SEC24A,SEC24B, SEMA5A, SERINC1, SGSH,SHPRH,SIL1(includesEG:100334837),SIPA1L2,SIPA1L3,SLC17A2, SLC23A2,SMEK2,SMOX,SSU72,STK19,TBC1D15,TCTN2,TFAP4,TFR2,TGM3,THAP5, TIPRL, TOP3B,TRIM17,TRIM44,TUBG2,*UBC*,USP44,VEZT,VPS37A,ZDHHC8,ZMIZ1,ZNF259
4: Post-Translational Modification, Carbohydrate Metabolism, Lipid Metabolism. Ingenuity score: 74, 67 focus molecules.	AGXT2L1,AHCYL2,ANGEL1,ANKRD17,ANKRD34A,ARMC9,ASXL1,ASXL2,AURKAIP1,B3GALT6,BAP1,BTN3A2,C15orf41,C1orf112,C1orf198,C2CD2,C9orf106,CCDC86,CCNDBP1,CCPG1,CENPP,COX20 (includesEG:116228),CTU2,CXXC4,DAK,DENND3,DHRS3,DLST,EFR3B,ELF2,ENDOV,FBXL4,FIS1 (includes EG:288584), FTO,GDAP1,HAT1,HSPA2,IER3IP1,IFIH1,IMP3,INPP5A,INPP5B,INPP5E,INPP5K,IPO4,IPPK,KANK4,KCNK10,KDM1B,KHNYN,LMBR1,MB21D2,MCF2L,MED20,METTL10,MRPL15 (includes EG:27395),MRPS18A,MRPS9 (includesEG:301371),MTCH1,MTMR8,MTMR9,PABPC5,PAN2,PITRM1,PMPCA,POLR3GL,PPM1G,PRDM10,PRPSAP2,PRUNE2, PSMB10, PSTK,PUSL1,PXDNL,RCN3,RPL28,SAR1B,SCAF4,SCNN1D,SDR39U1,SEC13,SEC16A,SEC16B,SEC23A,SIRT6,SLC35C2,SLC39A10,SLC45A4,SLC6A3,SYF2 (includes EG:170933),SYNGR1,SYNJ2,TAPT1,TMEM70, TMEM132A,TPTE/TPTE2,TRMT5,TSPAN9,TTYH2,TTYH3,TXNDC15,*UBC*,UBE2O,UCHL1,UCHL3,USP2,USP3,USP5,USP6,USP10,USP13,USP16,USP18,USP21,USP24,USP25,USP28,USP30,USP32,USP33,USP34,USP35,USP36,USP37,USP38,USP40,USP42,USP44,USP45,USP47,USP48,USP53,USP54,USP27X,USP9Y,XXYLT1,ZC3H13,ZNF131,ZNF334,ZNF608

The results of the network analysis included our genome-wide significant finding (*DUSP10*) within the top scoring network. P38 MAPK which interacts with DUSP10 is included in the second top network. Also the MHC class II complex is part of the second network. Genes within ours (P38 MAPK and DUSP10) and previously identified genome-wide significant regions are marked in italic, bold text. Only bold text show genes involved in amoebiasis. Underlined genes showed differences in our gene expression analysis (Table 9).

Rank; Top functions; Ingenuity score; Number of focus molecules; Molecules in Network.

### Gene Expression

**Figure 4:**
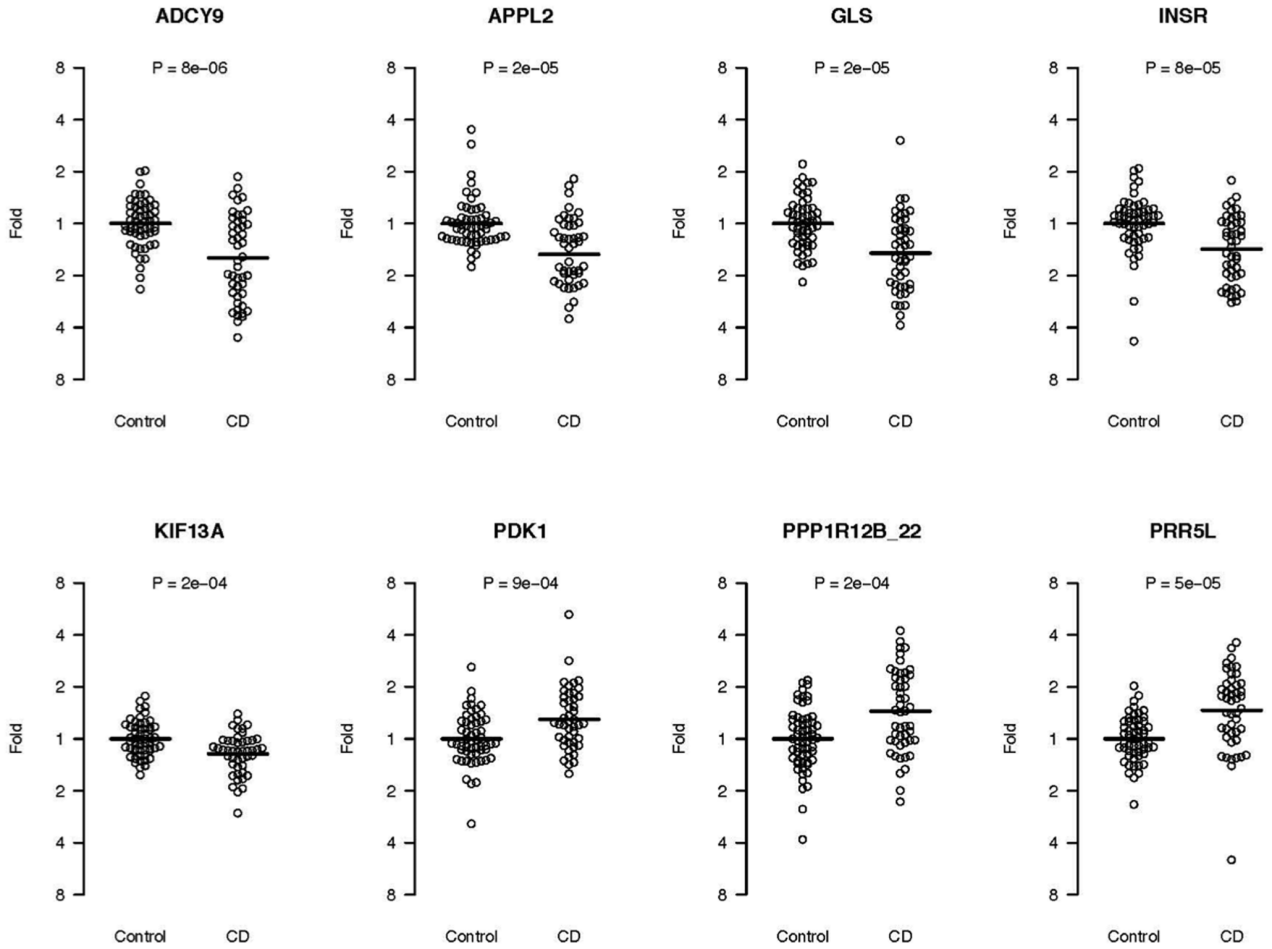
Gene expression results. Fold change on the y-axis is plotted for each individual in the two groups, 46 CD cases and 52 control patients. Each circle in the graph represents an individual. The mean expression value of the control group is set to 1.

Out of the 34 selected target genes, three were from the top associated SNPs (*DUSP10, SVIL* and *PPP1R12B*) and the remaining were genes identified from the two-locus and pathway analysis. Eight genes showed significant up- or down-regulation after correction for multiple testing using Bonferroni correction (Fig. 4). For the top associated genes, several transcript variants were tested (Table 9). For the *PPP1R12B* gene, Isoform c and d (transcript variants NM032103.2 and NM032104.2) also known as the small subunit (sm-M20) of myosin light chain phosphatase, show significant up-regulation in patients with CD autoimmunity compared to control patients. An additional ten genes showed nominally significant differences in expression (Table 9).

**Table 9:** Results from gene expression analysis of 34 candidate genes.

Gene symbol	Assay id	Gene	Fold Change		p-value	p-value corr.^b^	Selection criteria
ADCY9^a^	Hs00181599_m1	adenylate cyclase 9	1.58	DOWN	7.55E-06	2.87E-04	two-locus
APPL2^a^	Hs00216855_m1	adaptor protein, phosphotyrosine interaction, PH domain and leucine zipper cont. 2	1.51	DOWN	2.15E-05	8.19E-04	two-locus
GLS	Hs00248163_m1	glutaminase	1.48	DOWN	2.20E-05	8.38E-04	two-locus/IPA/top previous
PRR5L	Hs01029928_m1	proline rich 5 like	1.46	UP	4.99E-05	1.90E-03	upreg TNFa
INSR	Hs00961554_m1	insulin receptor	1.41	DOWN	8.39E-05	3.19E-03	two-locus
KIF13A	Hs00223154_m1	kinesin family member 13A	1.22	DOWN	1.76E-04	6.68E-03	two-locus
PPP1R12B	Hs00364073_m1	protein phosphatase 1, regulatory (inhibitor) subunit 12B	1.44	UP	2.05E-04	7.78E-03	top
PPP1R12B	Hs00364078_m1	protein phosphatase 1, regulatory (inhibitor) subunit 12B	1.15	UP	7.75E-04	0.029	top
PDK1	Hs01561850_m1	pyruvate dehydrogenase kinase, isozyme 1	1.30	UP	9.42E-04	0.036	IPA
FABP1	Hs00155026_m1	fatty acid binding protein 1, liver	1.38	DOWN	1.63E-03	0.06	two-locus
RGS2^a^	Hs01009070_g1	regulator of G-protein signaling 2	1.26	DOWN	2.32E-03	0.09	IPA/top previous
DPP10	Hs00397766_m1	dipeptidyl-peptidase 10	1.75	DOWN	4.22E-03	0.16	two-locus
IKBKE	Hs01063858_m1	inhibitor of kappa light polypeptide gene enhancer in B-cells, kinase epsilon	1.18	UP	0.014	0.52	two-locus/IPA/genetrail
UNC5C	Hs00186620_m1	unc-5 homolog C (C. elegans)	1.17	DOWN	0.015	0.59	IPA
ARID1B	Hs00368175_m1	AT rich interactive domain 1B	1.13	DOWN	0.017	0.63	two-locus
PKN2	Hs00178944_m1	protein kinase N2	1.14	DOWN	0.024	0.91	two-locus
ITPK1	Hs00356546_m1	inositol 1,3,4-triphosphate 5/6 kinase	1.13	DOWN	0.026	0.99	two-locus
RGS1	Hs00175260_m1	regulator of G-protein signaling 1	1.41	UP	0.026	1.00	IPA/top previous
SVIL	Hs00931734_m1	supervillin	1.34	DOWN	0.035	1.00	top
PPP1R12B	Hs00981888_m1	protein phosphatase 1, regulatory (inhibitor) subunit 12B	1.08	DOWN	0.053	1.00	top
APPL1	Hs00989616_m1	adaptor protein, phosphotyrosine interaction, PH domain and leucine zipper cont. 1	1.10	DOWN	0.055	1.00	two-locus/IPA/genetrail
MTTP	Hs00165177_m1	microsomal triglyceride transfer protein	1.21	DOWN	0.100	1.00	IPA
COQ3	Hs00213616_m1	coenzyme Q3 homolog, methyltransferase (S. cerevisiae)	1.23	DOWN	0.196	1.00	two-locus
SVIL	Hs00931014_m1	supervillin	1.18	DOWN	0.221	1.00	top
CD200	Hs01033303_m1	CD200 molecule	1.09	UP	0.368	1.00	IPA
MAGED1	Hs00986269_m1	melanoma antigen family D, 1	1.04	DOWN	0.512	1.00	IPA/genetrail
FOXD3	Hs00255287_s1	forkhead box D3	1.13	UP	0.547	1.00	two-locus
ITPK1-AS1	Hs01053867_s1	inositol 1,3,4-triphosphate 5/6 kinase Associated	1.10	DOWN	0.548	1.00	two-locus
LPP	Hs00194400_m1	Lipoma-preferred partner	1.07	DOWN	0.634	1.00	two-locus/top previous
RGS5	Hs00186212_m1	regulator of G-protein signaling 5	1.04	DOWN	0.699	1.00	IPA
DUSP10	Hs00200527_m1	dual specificity phosphatase 10	1.12	UP	0.704	1.00	top
FSCB	Hs03044256_s1	fibrous sheath CABYR binding protein	1.12	DOWN	0.735	1.00	two-locus
CCR3	Hs00266213_s1	chemokine (C-C motif) receptor 3	1.05	UP	0.737	1.00	two-locus
TIPRL	Hs00295580_m1	TIP41, TOR signaling pathway regulator-like (S. cerevisiae)	1.01	DOWN	0.752	1.00	genetrail
KHDRBS2	Hs01061150_m1	KH domain containing, RNA binding, signal transduction associated 2	1.06	UP	0.840	1.00	two-locus
GTF2B	Hs00976258_m1	general transcription factor IIB	1.03	UP	0.888	1.00	IPA/genetrail
ACTN1	Hs00998100_m1	actinin, alpha 1	1.01	DOWN	0.914	1.00	two-locus
DUSP10	Hs04189838_m1	dual specificity phosphatase 10	No expression detected				top

Expression (e.g. mRNA levels) of these genes was either up- or down-regulated in small intestinal biopsies from CD cases compared with control patients. Effect direction is presented for cases with control group as a reference. The selection column indicates if the gene was selected due to its presence in two-locus or pathway analyses. “Top” indicates top SNP in the present GWAS and “top previous” indicates that it was present in the GWAS by Dubois et al.

All the gene assays (primers and probes) were predesigned and ordered from Life technologies (CA, USA).

Reference genes tested were: ACTB (Hs00357333_g1), B2M (Hs99999907_m1), EPCAM (Hs00158980_m1), GUSB (Hs99999908_m1), HPRT1 (Hs99999909_m1), MUC1 (Hs00159357_m1), PGK1 (Hs99999906_m1). For the results a combined value of ACTB, EPCAM, and PGK1 showed to be optimal when analysed by GeNorm and were selected as reference.

a Gene not included in the gene list used for pathway analyses due to recombination between associated SNP and gene promotor: rs10861406.

(APPL2), rs882820 (ADCY9). However, possible regulatory site could be close to associated SNP and influence gene expression.

b P-values corrected using Bonferroni correction.

### Non-parametric Linkage (NPL)

**Figure 5:**
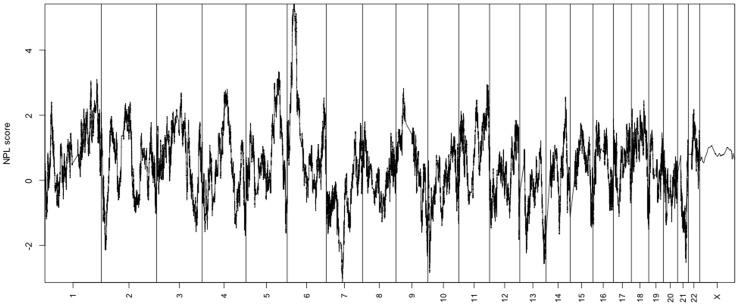
NPL results. Non-Parametric Linkage score displayed as –log10(p-value) on the y-axis and chromosome 1–22 and X on the x-axis.

The strongest linkage outside of HLA was detected in chromosome regions 5q23.2-q33.1, and 1q32.1. In total, thirteen regions with an NPL point wise p-value below 0.01 were detected (Fig. 5 and Table 10). In our previous linkage-scan, using almost the same set of families, we detected only one region (11q23-25) with a point wise p-value below 0.01 [14]. The reason for the improved results is mainly the almost perfect information content achieved by a dense set of highly successful SNP markers compared to a relatively sparse set of less successful microsatellite markers. Also in the NPL analysis, the *PPP1R12B* gene was located in one of the top regions (1q32.1).

**Table 10:** Non Parametric Linkage (NPL) results.

chr	from(Mb)	to(Mb)	max NPL	p-value
6^a^	12,5	52,6	5,42	3,03E-08
5	124,5	149,3	3,33	4,36E-04
1	200,2	231,8	3,12	9,20E-04
11	122,2	130,2	2,95	1,59E-03
9	30,3	34,7	2,82	2,40E-03
4	96,5	111,3	2,81	2,46E-03
3	104,7	108,6	2,70	3,49E-03
14	85,7	86,4	2,57	5,16E-03
6	160,4	161,0	2,54	5,50E-03
11	77,6	78,4	2,48	6,64E-03
18	55,0	55,1	2,45	7,20E-03
1	29,7	29,9	2,42	7,87E-03
2	127,0	127,1	2,41	8,00E-03
2	106,3	106,4	2,37	8,89E-03

Regions showing significant linkage (the HLA region only) and putative linkage (nominal p<0.01. Regions in the table are defined as the Megabase (Mb) interval showing a nominal p<0.01. Neighbouring regions were merged if <15 Mb pairwise distance.

Max NPL – the maximum Z score across the region between the positions ‘from’ and ‘to’.

p-value – the p-value for the max NPL score.

a = The HLA region.

## Discussion

This study confirmed some previous GWAS findings and in addition, it established a new genome-wide significant region containing the *DUSP10* gene. The top markers, rs12144971 and rs4240931 showed a substantial effect size in the HLA low-risk group with a transmitted versus non-transmitted allele ratio of 3.11 (Table 2).

### DUSP10, TNF-α and Tissue Transglutaminase (TGM2)

The protein product of *DUSP10* preferentially binds to the stress-activated p38 MAPK (mitogen-activated protein kinase) and plays an important role in regulating chemokine induction after infection by various pathogens [15], and in coordinating MAPK activity in response to oxidative stress [16]. In previous studies, both p38 MAPK and DUSP10 have been shown to activate TNF-α [17], [18], of which one also demonstrates that TNF-α up-regulates *TGM2* (the gene encoding the main autoantigen in CD [19]) in intestinal mucosa from untreated CD patients [17]. Whether this up-regulation of *TGM2* is of importance for the immune response leading to formation of IgA-tTG and IgG-tTG autoantibodies, the serological markers for CD is still unresolved.

### Pathway Analyses

In order to discover possible functional connections between *DUSP10* and other genes, we analyzed genes surrounding the top 603 markers. A total of 845 genes were used in the analysis. Ingenuity pathway analysis (IPA) included *DUSP10* within the most significant network. Also part of this network were *GLS* and *RGS1,* two genes previously identified within significant GWAS loci [3], as well as the *insulin (INS)* gene, and the immune regulatory nuclear factor kappa B (NF-Κb) complex (Fig. 3 and Table 8). The second top network included the MHC complex (HLA) and also several genes within already identified GWAS loci: *ACTN1, CD247, CCR5, ICOS* and *STAT1* [3]. In addition, both IPA and GeneTrail [13] identified T2D genes as the most significantly overrepresented gene cluster after correction for multiple testing (Table 5 and 6). Among this set of genes surrounding the 603 markers, many genes belonged to growth and nutrient signaling pathways, for example, *INS*, *INSR*, *EGF*, *POMC*, *TIPRL and PRR5L*. There were also related genes directly involved in energy metabolism; *PDK1, COX7C, COQ3* and *GLS*.

### Overlapping Results with Other GWAS Findings

Surprisingly, four out of six top loci identified by a GWAS for anorexia nervosa [20] and two out of three loci involved in plasma glucose levels in type 1 diabetic patients [21] were among our 603 and 35 best SNP markers respectively. One of the genes in anorexia, namely *AKAP6, is also associated to* fasting insulin-related traits as well as the autoimmune disease Ankylosing spondylitis [22]. Of the 40 identified regions in CD, seven regions overlap with our 603 SNP list (*LPP, STAT4/GLS, RGS1, CCR1/CCR3, PUS10, ICOS/CTLA4* and *CD247*). Out of the 69 regions reported in the GWAS catalog for type 1 diabetes, eight overlap with the regions reported in this study and out of those eight, *CTLA4/ICOS* also overlap with the previously reported CD associations.

We compared minor allele frequencies between the previous CD GWAS by Dubois et al. and our GWAS. In their top 42 associations, there was no SNP below a minor allele frequency of 0.08. In our top 42 associations, we identified five SNPs with a minor allele frequency below 0.06. This observation could just be a chance finding or perhaps an indication that rare variants are easier to discover using families. We also identified a relatively rare variant in the LPP gene region (rs17283813), with a minor allele frequency of 0.075. This SNP was not at all significant in the GWAS by Dubois et al. (Table S1).

Neither was there an association with the *DUSP10* region in the GWAS by Dubois and co-workers. The associated markers in the *DUSP10* region in our GWAS have a minor allele frequency around 0.5 and are hence very common in the population. It is difficult to say if this is a population specific effect or if *DUSP10* could be detected in an HLA stratified population from another ethnicity. Interestingly, the *DUSP10* region has also been identified as a risk factor for colon cancer by a meta-analysis of three GWAS from the UK. This is an indication that colon cancer and CD could share genetic risk factors.

### Key Metabolic Regulators as well as the Top Associated gene PPP1R12B were Differently Expressed in CD Cases Compared to Controls

Another important finding was the difference between cases and controls and their gene expression patterns in the small intestine. Eight of the 34 candidate genes selected for quantitative measurements of gene expression, including *PPP1R12B, PDK1*, *GLS*, *PRR5L* and the *INSR*, showed significant up or down regulation of mRNA levels in cases compared to controls (Fig. 4). This could very well be a consequence of an ongoing inflammation or possibly also indicate an underlying metabolic difference. Glutamine is converted to glutamate by the enzyme glutaminase (GLS). In turn, glutamate can be converted to proline and subsequently catabolized by the enzyme proline dehydrogenase (PRODH) resulting in the production of reactive oxygen species and apoptosis [23]. In the present study, we show that the expression of *GLS* is down-regulated and *PDK1* is up-regulated in cases. Interestingly, a previous study has shown that cell lines with a known familiar mutation for amyotrophic lateral sclerosis (ALS) have the same expression pattern, with up-regulated *PDK1* and down-regulated *GLS,* as compared to the wild-type cell line [24]. *PRR5L* (also called Protor-2) belong to the TOR signaling pathway. Our results show an up-regulated *PRR5L* expression in cases (Fig. 4). Like DUSP10, the protein product from *PRR5L* has been shown to stimulate an increased TNF-α expression [25].

Another gene, connected to the MAPK pathway and which was identified both by our two-locus interaction analysis and in significant biological functions implied by IPA, was the *APPL1* gene. *APPL1* is a binding partner of the protein kinase Akt2 and a key regulator of insulin signaling [26]. It takes part in adiponectin signaling to stimulate activity of p38 MAPK in muscle cells [27] and is a critical regulator of the crosstalk between adiponectin signaling and insulin signaling pathways [28]. We could detect expression of both *APPL1* and *APPL2* in small intestinal biopsies and a significantly lower expression of *APPL2* was detected in the CD autoimmunity cases as compared to controls (Fig. 4). Lower expression of *APPL2* levels lead to enhanced adiponectin stimulated glucose uptake and fatty acid oxidation [29]. A SNP (rs10861406) included in the top 603 list was located upstream of the *APPL2* gene, however the promotor of this gene was on the opposite side of a recombination hotspot and therefore not included in the gene list for pathway analyses.

The most significant finding from our non-stratified linkage GWAS analysis was the association with the *PPP1R12B* gene region. *PPP1R12B* is involved in smooth muscle contractibility and mediates binding to myosin [30]. Myosin light chain phosphatase from smooth muscle consists of a catalytic subunit (PP1c) and two non-catalytic subunits, M130 and M20. The two non-catalytic subunits are both encoded by the *PPP1R12B* gene. The M130 transcript was not differentially expressed between CD autoimmunity and control patients while the small subunit “M20” showed a significantly higher expression in patients with CD autoimmunity. (*PPP1R12B*_22 in Fig. 4) Several other genes located close to top markers such as the *PPP3CA, ACTN1, MYO1B, MYO5A, MAPK1, PRKCH, PRKCQ, PRKACB, PRR5L and NTS genes,* are connected to smooth muscle when examining their function by using *KEGG* [31] and Gene Ontology [32].

The second most significant region in the HLA-stratified analysis after *DUSP10* contains the *SVIL* gene. The product of this gene has been suggested to bind LPP [33]. In our two-locus interaction analysis, the *LPP* locus and a locus containing *KIF13A* was one of the 101 interaction pairs. *KIF13A* is a motor protein, which shuttles vesicles containing AP-1 and the mannnose-6-phosphate receptor [34]. *KIF13A* was significantly down-regulated in intestinal biopsies from CD patients in our gene expression analysis (Fig. 4). *SVIL* is associated with cell-focal adhesions (substrate contacts), which are important for rapidly moving cells such as for example immune cells but also for motility and polarity of intestinal epithelial cells. *SVIL* mRNA was down-regulated in our gene expression analysis, however, not significant after correction for multiple testing.

### Proline and Glutamine Metabolism - Part of a “Danger Signal”

Amoebiasis was one of the nominally significant pathways in the GeneTrail analysis of genes surrounding the two-locus interaction SNPs (Table 7). Several of these genes were also present together with *DUSP10* and the MHC class II genes in the two most significant IPA generated networks (marked in bold text in Table 8). Another gene present in these networks was the gene encoding for the immune molecule CD40 (associated SNP rs6065961, Table S1). CD40 has been shown to regulate immune responses to another parasite, Leishmania Major, by shared signaling through p38 MAPK and ERK1/2 [35]. CD40 also regulates DUSP expression and activity, which in turn contribute to anti-leishmanial functions [35]. It has been suggested that Leishmania Major inhibits CD40-triggered p38 MAPK signaling as part of an immune evasion strategy [36].

Another overrepresented category from GeneTrail was the extracellular matrix (ECM) (Table 6). Also, in the two most significant Ingenuity networks from the 603 marker analyses, ECM molecules and matrix metalloproteinases (MMPs) were included (Table 8). The ECM represents a major barrier to parasites like amoebas and leishmania. Parasites produce a wide variety of proteases to break down the ECM in order to access essential nutrients and invade host tissue [37]. A different situation when the ECM is degraded is during nutrient deprivation. In this way the ECM can provide energy for starving host cells. Just like gluten, the ECM has an unusually high proline content. MMPs are enzymes, which break down ECM making proline readily available as a nutritional source. Pandhare and co-workers have shown that energy or nutrient stress activates MMPs as well as the degradation of proline and furthermore demonstrated that, as the levels of glucose decreased to 1 mM and lower in the medium, intracellular proline increased almost 2-fold [38]. If gluten lingering in the intestine conveys a signal of ECM degradation (due to increased proline levels), several other mechanisms will most likely signal that there is food available at the same time (salivary secretion as one example is shown in Table 6). In this case, the immune system will rule out starvation as a possibility and the only other sensible option would be to search for an invasive intruder breaking down the ECM. The autoantigen in CD, TGM2, counteracts proteolysis and degradation of ECM by crosslinking ECM proteins [39]. If DUSP10 and PRRL5 up-regulate TNF-α and subsequently TGM2 [17], [18], [25], in CD, the purpose may very well be for TGM2 to help prevent an apparent or illusory pathogenic invasion. It has also been shown that down-regulation of SVIL protects against ECM invasion by pathogens [40]. In our gene expression analysis SVIL was nominally significantly down-regulated in cases (Table 9).

When the body “senses” a pathogen disturbing energy balance or breaking down ECM, but there are no pathogenic antigens present, maybe there could be a risk that “self” antigens become our immune systems futile attempt to rid the perceived pathogen. In HLA-DQA1*02/05 and HLA-DQB1*02 carriers, peptides derived from TGM2 could constitute such “self” antigens. It is possible that individuals carrying other HLA molecules still respond to this “phantom pathogen” and that under these circumstances, various other antigens present in the intestine at the time could become triggers of other autoimmune diseases. If the expression or presence of an autoantigen, like TGM2, was stimulated by the disturbed proline/glutamine homeostasis, it can explain why symptoms in CD also disappear by withdrawal of gluten.

### Conclusion

At least four major functional components together with gluten, all seem to play a role in forming an individual’s risk for CD:

1. polarity and epithelial cell functionality, e.g. nutrient/vesicle transport, proliferation and apoptosis, important for cell migration from the crypt to the shedding (apoptosis) at the apical villi.
2. intestinal smooth muscle, which is important for the movement of the bowel as well as the villi.
3. growth and energy homeostasis, which includes proline and glutamine metabolism, and finally
4. the innate and adaptive immune system.

**Figure 6:**
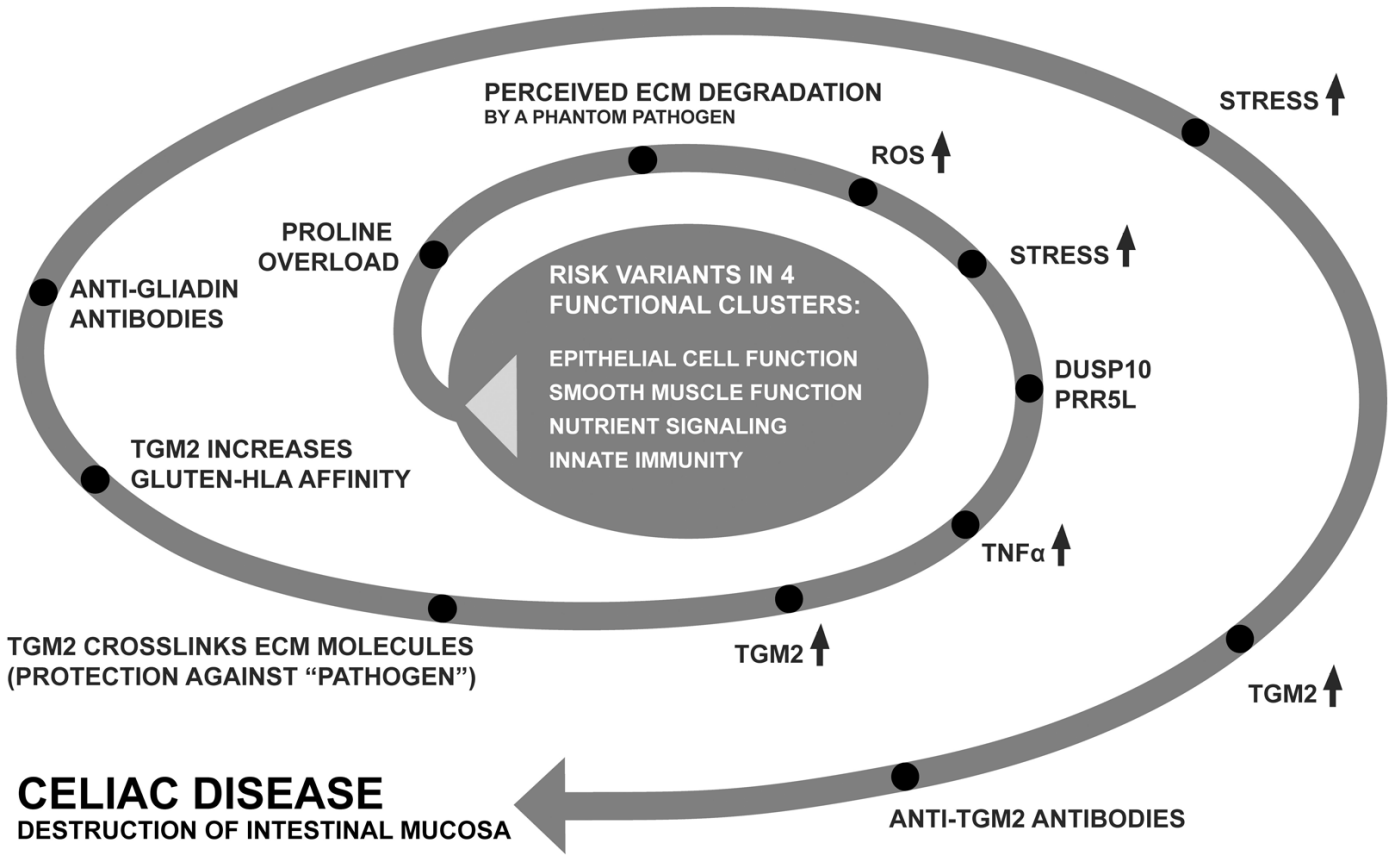
Proposed disease model. Illustrating a possible scenario for disease development. Genetic variation contributing susceptibility to disease can be found in at least four, somewhat overlapping, biological functions. The result is an “overload” or imbalance of proline vs glutamine. Due to the abundance of proline within the extracellular matrix (ECM) as well as in gluten, the proline from gluten is interpreted as degradation of ECM. When the body is not starving, the ECM is normally not degraded, unless there is a pathogen attempting to break through this barrier. The immune system mounts an attack against an invasive “phantom pathogen” which is believed to degrade the ECM. When proline is catabolized, reactive oxygen species (ROS) are released. In order to start re-building and crosslinking ECM molecules, Tissue transglutaminase (*TGM2*) expression is up-regulated by TNFα which in turn is stimulated by DUSP10 and Protor-2 (PRR5L). This rebuilding of the ECM counteracts the degradation by the imagined pathogen. However, the phantom pathogen remains and the adaptive immunity is brought in. Searching for antigens, it finds an abundance of TGM2 beside the ECM and forms antibodies against its own soldier. Some susceptibility genes can be found in the center of this model and some can be found within the spiral. Genes like *HLADQ* and other genes from the adaptive immunity are likely to be found in the spiral.

A slight dysfunction combining these categories together with gluten consumption would result in a metabolic imbalance which in turn could convey enough stress or “danger signal”, to trigger the immunological process and tissue destruction. A schematic illustration showing a rough outline of a possible disease model is presented in Figure 6.

In this study, we identified *DUSP10* to be significantly associated with celiac disease. We also identified mechanisms, which we believe influence the risk of developing disease. Our data points towards genes that are involved in cancer as well as metabolic and cardiovascular diseases. Besides understanding how they work in celiac disease, our findings could also have consequences for these other common diseases.

Whole genome analysis allows for discovering completely unknown mechanisms behind disease. Even if the discovered genes and gene variants won’t be able to predict who will develop disease in the future, they can be used to identify the underlying molecular pathways that influence disease. These molecular pathways would then be valuable targets for drug intervention. Our data provides new insights and hypotheses to the research field of CD and autoimmunity. However, the functional variants behind associations as well as mechanisms causing differences in gene expression and if and how these are relevant for disease, remains to be identified.

## Materials and Methods

### Ethics Statement

The regional ethics board in Gothenburg approved this study and participants in the study gave written informed consent after being fully informed about the aim of the study. For all children in the study, parental written consent was obtained.

### Study Population

A total of 106 families with multiple affected individuals, mostly nuclear families with an affected sib pair (ASP) were collected from Sweden and Norway. There were 403 subjects and 97 families with DNA to complete the analysis. A total of 226 of the family members had CD, including 20 parents. The makeup and selection process regarding the families has been described previously in detail [41].

Small-intestinal biopsies, for the gene expression analysis, were collected at four pediatric clinics in Sweden: Skåne University Hospital in Malmö, Sach’s Childrens’ Hospital and Karolinska University Hospital in Stockholm and Sahlgrenska University Hospital in Gothenburg. For gene expression calculations, we decided to use case-control status based on anti-tTG IgA and IgG antibody levels in plasma using the Radio-Binding Assay (RBA) [42], [43]. Patients being both tTG IgA and IgG positive (>4 U/ml) were included as cases (we use the term “CD autoimmunity” for these cases [44]) and all other individuals were included as disease controls. The LPP gene was run in a first set of 42 cases (30 females, 12 males) and 38 control patients (21 females, 17 males). Cases and controls had a mean age of 7.3 and 11.1 years respectively. The remaining genes were run in a second set of 46 cases (25 females, 21 males) and 52 control patients (27 females, 25 males) with a mean age of 7.4 years for cases and 11.8 years for control patients at the time the biopsy was taken.

### Gene Expression Analysis

We performed quantitative gene expression analysis using duodenal biopsies from CD autoimmunity patients and control patients. Biopsies were immediately put in RNAlater solution (Life Technologies, CA, USA). Total RNA was extracted using the miRNeasy Mini Kit (QIAGEN, Germany). RNA was converted to cDNA and quantitative PCR was run using TaqMan chemistry and the ABI7900 SDS instrument (Life Technologies, CA, USA). Seven control genes were evaluated using GeNorm [45] (ACTB (Hs00357333_g1), B2M (Hs99999907_m1), EPCAM (Hs00158980_m1), GUSB (Hs99999908_m1), HPRT1 (Hs99999909_m1), MUC1 (Hs00159357_m1), PGK1 (Hs99999906_m1)) and the geometrical mean of *ACTB, EPCAM*, and *PGK1* were selected as reference for the relative quantification analysis (Delta-Delta Ct method). A total of 34 expressed genes located close to some of the most significantly associated SNPs were evaluated (Table 9). The top associated genes from our Linkage GWAS (*DUSP10, SVIL, PPP1R12B*) were selected as well as several genes from the two-locus interaction analysis and pathway analyses including *LPP* which is the top associated from the GWAS by Dubois et al. Also the RGS genes (*RGS1, 2 and 5*) and *GLS* show genome-wide association in the study by Dubois et al and are also present in our two-locus and pathway analyses.

### Genotyping and Imputation

Samples were genotyped using two different SNP arrays, 211 samples with Human Omni Express and 192 samples with Human 660W-Quad (Illumina Inc, CA, USA). A total of 308,246 markers were available on both arrays and were therefore genotyped in the entire material. For the remaining 682,470 and for sporadic missing values we performed genotype imputation using the Impute 2 software [46], with the Hapmap 2 (rel. 24 Build 36) as a reference.

All individuals in the same family were located on the same plate. Quality control was first performed separately for the two arrays. SNP markers with less than 97% call rate in either of the two arrays were excluded.

Mendelian errors were detected by PLINK [47], 125,874 family-wise mendelian inconsistencies were set to missing (in each family the genotypes were set to missing for all subjects if there were any mendelian inconsistencies for a specific SNP).

### Statistical Analysis

#### Linkage

For the linkage analysis only markers from both platforms were considered. From this set of 271,078 common SNP markers a LD pruned set of 105 539 SNPs were selected using PLINK. Parameters were a window size of 50 and R^2^<0.5. The Decode genetic map as supplied by Illumina was used to run non-parametric linkage using Merlin version 1.1.2 [48] with the NPL all method [49]. Marker allele frequencies were estimated from the founders.

#### Transmission Disequlibrium Test (TDT)

Spielman et al introduced the Transmission Disequilibrium Test (TDT) in 1993 [50].

The imputation analysis provides us with (posterior) probabilities for each of the possible genotypes at each locus and to utilize all posterior probabilities, we performed an analysis where we use the expected values of the transmission counts. The test statistic will then have the following form,
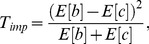
(1)



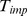
 has approximately the same distribution as the test statistic *T* in [50].

#### Stratified TDT

We implement a stratified TDT analysis where trios are split into a low-risk and a high-risk group based on the HLA genotype of the affected offspring. Children carrying the HLA-DQA1*02/05 risk allele and homozygous for the HLA-DQB1*02 risk allele (i.e. individuals carrying the DR3/DR3 or the DR3/DR7 haplotypes) were put in the “high-risk” group and the remaining children were put in the “low-risk” group. The rationale behind this is explained in the introduction and further information about this stratification can be found in our previous Linkage study [14]. A standard TDT analysis, with the 0.95 cut-off for imputation probabilities, was applied to each of these groups using PLINK [47].

#### Test for two-locus interaction

To examine possible interactions between marker variants, we used a pairwise test based on the one introduced by Kotti [51]. Consider two biallelic markers without linkage disequilibrium between their alleles. In the general model M_G_ the penetrance matrix has 9 parameters,
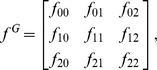



Let **n** be the 3×3 matrix of genotype counts among the cases for the two markers, and let **m** be the corresponding matrix for the non-transmitted allele combinations. The likelihood for the models is
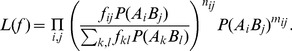
(2)


For this analysis we use one affected subject from each family and markers were chosen based on the expected counts TDT (equation 1) and three different inclusion criteria:

1. P-value less than 3.0×10^−4^.
2. P-value less than 0.01 in our analysis and with a p-value less than 0.05 in the GWAS by Dubois et al. [3] and if the product of these p-values were less than 5.0×10^−5^ and the association were in the same allelic direction.
3. An allele transmission ratio of <0.2 or >5 combined with a p-value less than 2×10^−3^.

We defined 383 regions using the inclusion criteria above (Fig. 2 and Table S1) a region consisted of a set of markers where the distance between adjacent markers was less than 100 kb. With these regions defined we analyzed all pairwise interactions using a *Likelihood Ratio* (LR) tests comparing the following four models:

- M_0_: None of the two loci is associated with CD,
- M_R_: Heterogeneity model [52], with penetrance

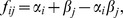
where α_i_ and β_j_ are the penetrance factors for the genotypes A_i_ and B_j_ [53] respectively.

- M_M_: Multiplicative model,
- M_G_: the general model.

The restricted model used in [51] is the multiplicative model. We use the M_G_-versus-M_M_ test to filter out false positives, based on that if one or both of the SNPs were marginally significant by chance, then the joint distribution (penetrance) of these markers should follow a multiplicative model.

We have the likelihood ratio statistic
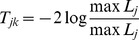


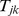
 will follow a *x* ^2^ distribution under the restricted model if M_j_ is nested in M_k_. The maximum likelihood estimates of the penetrance parameters and allele frequencies do not have a simple explicit expression, so to maximize the likelihoods we use the function *optim* in the statistical software R.

### Gene Selection

Out of the 603 SNPs selected from the three inclusion criteria (Fig. 2 and Table S1), we were able to identify genes surrounding 444 SNPs using GRAIL [54]. Grail uses known recombination hotspots in order to limit the region of interest surrounding each SNP marker. Genes around the remaining SNPs were identified with the Genome Browser (http://genome.ucsc.edu) and the 5 closest genes within 250 kb from the associated SNPs were included. In cases where there were no genes within this distance we included the closest gene.

### Pathway Analysis

We analyzed connections between genes in different regions, using GeneTrail [13] and the Ingenuity Pathway Analysis (IPA) software (Ingenuity Inc. CA, USA). Within each associated region, all but one gene from the same gene family were removed. This was done in order not to amplify the significance of homologous gene clusters, (i.e. chemokine receptor-, interferon- and histone-gene clusters).

### URLs

PLINK (http://pngu.mgh.harvard.edu/purcell/plink/)

KEGG (www.genome.jp/kegg/)

Gene Ontology (www.geneontology.org/).

GWAS catalog, http://www.genome.gov/gwastudies/

GRAIL (http://www.broadinstitute.org/mpg/grail/)

SNAP (http://www.broadinstitute.org/mpg/snap/ldplot.php/)

GeneTrail (http://genetrail.bioinf.uni-sb.de/)

## Supporting Information

Table S1 **A selection of 603 top associated SNPs and the two top HLA SNPs.** Based on three inclusion criteria, 603 SNP markers and 383 regions were identified. Our TDT results, where T and U are the expected transmission counts (based on all the posterior imputation probabilities) and corresponding results from Dubois et al. [3].
(DOCX)
