# Supplementary material for: A Possible Mechanism behind Autoimmune Disorders Discovered By Genome-Wide Linkage and Association Analysis in Celiac Disease
Source: PLoS One. 2013 Aug 2;8(8):e70174. doi: 10.1371/journal.pone.0070174 (PMC3732286; doi:10.1371/journal.pone.0070174)
Supplement: Table S1 — A selection of 603 top associated SNPs and the two top HLA SNPs. Based on three inclusion criteria, 603 SNP markers and 383 regions were identified. Our TDT results, where T and U are the expected transmission counts (based on all the posterior imputation probabilities) and corresponding results from Dubois et al. [3]. (DOCX) [file pone.0070174.s001.docx]

**Table S1** A selection of 603 top associated SNPs and the two top HLA SNPs

|  |  |  |  |  |  |  |  |  |  |  | **Dubois** | **et al.** | **2010** |  |  |
| --- | --- | --- | --- | --- | --- | --- | --- | --- | --- | --- | --- | --- | --- | --- | --- |
| **CHR** | **SNP** | **GENES** | **BP** | **A1** | **A2** | **T** | **U** | **T/U** | **chisq** | **p-value** | **A1** | **A2** | **p-value** | **OR** | **Incl. Criteria** |
| 6 | rs424232 | HLA | 32316302 | C | T | 47.7 | 194.03 | 0.246 | 88.59 | 4.87E-21 | NA | NA | NA | NA | 1 |
| 6 | rs204999 | HLA | 32217957 | A | G | 51 | 199 | 0.256 | 87.62 | 7.95E-21 | NA | NA | NA | NA | 1 |
| 1 | rs12734338 | PPP1R12B SYT2 UBE2T | 200736346 | C | T | 61.4 | 132.5 | 0.46 | 26.00 | 3.41E-07 | NA | NA | NA | NA | 1 |
| 10 | rs10886159 | EMX2OS RAB11FIP2 EMX2 | 119603600 | C | T | 40.2 | 98.5 | 0.41 | 24.54 | 7.30E-07 | NA | NA | NA | NA | 1 |
| 21 | rs10439884 | BAGE2 TPTE BAGE | 9993822 | A | G | 15.5 | 55.8 | 0.28 | 22.73 | 1.86E-06 | T | C | 0.212 | 1.06 | 1 |
| 14 | rs1958589 | EAPP SNX6 C14orf147 | 33914127 | C | T | 27.3 | 73.6 | 0.37 | 21.33 | 3.87E-06 | G | A | 0.672 | 1.02 | 1 |
| 17 | rs17760268 | ANKFN1 NOG | 51966290 | C | T | 57.2 | 17.5 | 3.28 | 21.20 | 4.13E-06 | T | C | 0.273 | 0.94 | 1 |
| 4 | rs1032355 | RG9MTD2 C4orf17 MTTP | 100758919 | C | T | 35.1 | 85.3 | 0.41 | 20.94 | 4.74E-06 | NA | NA | NA | NA | 1 |
| 22 | rs4911642 | CCT8L2 psiTPTE22 | 14884399 | C | T | 34.4 | 84.2 | 0.41 | 20.89 | 4.86E-06 | NA | NA | NA | NA | 1 |
| 20 | rs157640 | DOK5 | 52847946 | G | T | 72.0 | 138.0 | 0.52 | 20.74 | 5.25E-06 | T | G | 0.649 | 0.99 | 1 |
| 1 | rs2068824 | NAV1 | 199861288 | C | T | 6.8 | 36.8 | 0.19 | 20.57 | 5.75E-06 | NA | NA | NA | NA | 1 |
| 3 | rs2605393 | STAC | 36384605 | G | T | 58.5 | 118.9 | 0.49 | 20.53 | 5.86E-06 | NA | NA | NA | NA | 1 |
| 19 | rs2664156 | KLK2 KLK3 KLKP1 KLK4 KLK15 | 56068975 | C | T | 92.0 | 40.0 | 2.30 | 20.45 | 6.13E-06 | NA | NA | NA | NA | 1 |
| 16 | rs195656 | HYDIN | 69604985 | A | G | 29.8 | 76.1 | 0.39 | 20.24 | 6.83E-06 | NA | NA | NA | NA | 1 |
| 11 | rs4930144 | IGF2AS TH MRPL23 TNNT3 SYT8 ASCL2 TNNI2 LSP1 IGF2 INS H19 | 2005064 | A | G | 116.1 | 57.0 | 2.03 | 20.12 | 7.28E-06 | NA | NA | NA | NA | 1 |
| 4 | rs17029173 | RG9MTD2 C4orf17 MTTP | 100728344 | G | T | 27.1 | 71.5 | 0.38 | 20.02 | 7.65E-06 | NA | NA | NA | NA | 1 |
| 4 | rs13128441 | STK32B | 5213290 | C | T | 32.0 | 79.0 | 0.41 | 19.90 | 8.16E-06 | G | A | 0.020 | 1.08 | 1 |
| 3 | rs1871350 | STAC | 36348769 | C | T | 27.1 | 71.2 | 0.38 | 19.82 | 8.49E-06 | C | T | 0.937 | 1.00 | 1 |
| 3 | rs2046000 | STAC | 36327368 | A | C | 28.1 | 72.5 | 0.39 | 19.63 | 9.42E-06 | NA | NA | NA | NA | 1 |
| 17 | rs7209752 | CCDC144C LOC284194 SPECC1 AKAP10 | 19909989 | A | G | 12.6 | 46.5 | 0.27 | 19.41 | 1.06E-05 | T | C | 0.511 | 0.96 | 1 |
| 1 | rs3795277 | KIAA1751 PRKCZ GABRD | 1970978 | A | C | 46.6 | 12.8 | 3.65 | 19.31 | 1.11E-05 | NA | NA | NA | NA | 1 |
| 2 | rs10203748 | TGFBRAP1 C2orf49 NCK2 FHL2 GPR45 | 105442542 | C | T | 23.9 | 65.4 | 0.37 | 19.21 | 1.17E-05 | NA | NA | NA | NA | 1 |
| 11 | rs318966 | NTM | 130871348 | A | G | 33.9 | 80.8 | 0.42 | 19.21 | 1.17E-05 | NA | NA | NA | NA | 1 |
| 6 | rs9402234 | TMEM200A SAMD3 | 130869175 | C | T | 49.9 | 14.9 | 3.35 | 18.91 | 1.37E-05 | NA | NA | NA | NA | 1 |
| 9 | rs1536689 | C9orf93 BCN2* | 16119630 | A | G | 98.0 | 46.0 | 2.13 | 18.71 | 1.52E-05 | G | A | 0.644 | 1.01 | 1 |
| 4 | rs6838036 | DC2 AGXT2L1 RPL34 | 109630528 | A | C | 127.7 | 67.5 | 1.89 | 18.59 | 1.62E-05 | NA | NA | NA | NA | 1 |
| 3 | rs17283813 | LPP | 190122389 | A | G | 17.5 | 54.0 | 0.32 | 18.59 | 1.62E-05 | T | C | 0.635 | 0.98 | 1 |
| 3 | rs1871352 | STAC | 36329541 | A | C | 28.0 | 70.8 | 0.40 | 18.54 | 1.66E-05 | T | G | 0.907 | 1.00 | 1 |
| 1 | rs12747934 | FOXD3 | 63540185 | A | G | 24.9 | 65.6 | 0.38 | 18.32 | 1.87E-05 | *A* | *G* | *0.003* | *0.87* | 1 |
| 1 | rs4323662 | LOC100288079 IVNS1ABP | 183697117 | G | T | 98.4 | 46.9 | 2.10 | 18.30 | 1.89E-05 | NA | NA | NA | NA | 1 |
| 3 | rs1842149 | STAC | 36366714 | G | T | 61.3 | 22.2 | 2.76 | 18.28 | 1.91E-05 | NA | NA | NA | NA | 1 |
| 19 | rs3814892 | PALM HCN2 C19orf21 POLRMT FSTL3 PRSSL1 RNF126 FGF22 | 589853 | A | G | 23.5 | 63.0 | 0.37 | 18.12 | 2.08E-05 | NA | NA | NA | NA | 1 |
| 3 | rs12631757 | THRB | 24618577 | C | T | 80.1 | 34.5 | 2.32 | 18.11 | 2.09E-05 | T | C | 0.100 | 1.06 | 1 |
| 10 | rs7097380 | SORCS1 | 108671659 | A | G | 119.0 | 62.0 | 1.92 | 17.95 | 2.27E-05 | A | G | 0.887 | 1.00 | 1 |
| 1 | rs12734001 | PPP1R12B SYT2 UBE2T | 200657537 | C | T | 129.1 | 69.5 | 1.86 | 17.91 | 2.32E-05 | NA | NA | NA | NA | 1 |
| 10 | rs17094083 | GFRA1 | 117850841 | C | T | 30.8 | 74.2 | 0.42 | 17.89 | 2.35E-05 | C | T | 0.433 | 1.03 | 1 |
| 3 | rs12632771 | CX3CR1 | 39223856 | A | G | 36.0 | 8.0 | 4.50 | 17.82 | 2.43E-05 | G | A | 0.090 | 1.08 | 1 |
| 1 | rs316951 | LOC100129138 AMY1B | 104878333 | C | T | 25.0 | 65.0 | 0.38 | 17.78 | 2.48E-05 | A | G | 0.261 | 0.96 | 1 |
| 1 | rs34783763 | C1orf210 TIE1 | 43530349 | C | T | 17.7 | 53.2 | 0.33 | 17.75 | 2.52E-05 | NA | NA | NA | NA | 1 |
| 17 | rs11656673 | HRNBP3 | 74912218 | C | T | 75.3 | 31.8 | 2.37 | 17.63 | 2.68E-05 | T | C | 0.455 | 0.97 | 1 |
| 6 | rs10485047 | OPN5 GPR115 GPR111 | 48031204 | C | T | 5.0 | 29.7 | 0.17 | 17.60 | 2.73E-05 | NA | NA | NA | NA | 1 |
| 1 | rs11122534 | COG2 AGT CAPN9 C1orf198 PGBD5 | 228755089 | A | G | 66.0 | 25.8 | 2.56 | 17.57 | 2.77E-05 | NA | NA | NA | NA | 1 |
| 21 | rs4920059 | PRDM15 UMODL1 C2CD2 ABCG1 ZNF295 | 42331775 | C | T | 67.1 | 26.5 | 2.53 | 17.55 | 2.79E-05 | T | C | 0.867 | 0.99 | 1 |
| 11 | rs4755368 | ABTB2 | 34294918 | C | T | 19.6 | 56.1 | 0.35 | 17.55 | 2.80E-05 | C | T | 0.793 | 0.99 | 1 |
| 3 | rs1993923 | STAC | 36378955 | C | T | 89.0 | 41.2 | 2.16 | 17.53 | 2.83E-05 | NA | NA | NA | NA | 1 |
| 12 | rs2289954 | SLC6A13 JARID1A | 202623 | C | T | 93.1 | 44.1 | 2.11 | 17.51 | 2.85E-05 | NA | NA | NA | NA | 1 |
| 3 | rs1879903 | LPP | 190193761 | A | G | 18.7 | 54.2 | 0.34 | 17.35 | 3.11E-05 | A | G | 0.699 | 0.99 | 1 |
| 13 | rs3014904 | FLJ32682 COG3 | 45010493 | A | G | 38.0 | 84.0 | 0.45 | 17.34 | 3.12E-05 | G | A | 0.914 | 1.00 | 1 |
| 6 | rs9296204 | MTCH1 PI16 | 37052457 | C | T | 30.0 | 72.0 | 0.42 | 17.29 | 3.20E-05 | NA | NA | NA | NA | 1 |
| 25 | rs35271904 | PCDH11X PABPC5 | 91532610 | A | C | 30.0 | 72.0 | 0.42 | 17.29 | 3.20E-05 | NA | NA | NA | NA | 1 |
| 25 | rs2751017 | PCDH11X PABPC5 | 91692405 | A | G | 30.0 | 72.0 | 0.42 | 17.29 | 3.20E-05 | NA | NA | NA | NA | 1 |
| 14 | rs4905043 | ITPK1 CHGA | 92619762 | A | G | 119.0 | 63.0 | 1.89 | 17.23 | 3.31E-05 | A | G | 0.385 | 0.98 | 1 |
| 9 | rs1571812 | VLDLR | 2485870 | A | C | 48.6 | 98.9 | 0.49 | 17.21 | 3.35E-05 | NA | NA | NA | NA | 1 |
| 3 | rs7631897 | EIF4E3 GPR27 PROK2 FOXP1 | 72063333 | A | G | 58.0 | 112.1 | 0.52 | 17.19 | 3.39E-05 | NA | NA | NA | NA | 1 |
| 15 | rs12909478 | RYR3 | 31616542 | C | T | 98.2 | 48.1 | 2.04 | 17.18 | 3.40E-05 | A | G | 0.941 | 1.00 | 1 |
| 9 | rs13299141 | MLLT3 | 20451775 | C | T | 22.7 | 60.3 | 0.38 | 17.05 | 3.65E-05 | G | A | 0.881 | 0.99 | 1 |
| 6 | rs1205961 | PRL | 22393991 | A | G | 64.0 | 120.0 | 0.53 | 17.04 | 3.65E-05 | NA | NA | NA | NA | 1 |
| 15 | rs6495130 | RYR3 | 31597460 | A | G | 67.0 | 124.0 | 0.54 | 17.01 | 3.72E-05 | A | G | 0.469 | 0.98 | 1 |
| 1 | rs2790760 | DUSP10 | 220176141 | A | G | 71.1 | 129.5 | 0.55 | 16.98 | 3.77E-05 | A | G | 0.885 | 1.00 | 1 |
| 1 | rs12024611 | GTF2B PKN2 CCBL2 | 88845512 | A | G | 69.2 | 126.7 | 0.55 | 16.90 | 3.94E-05 | NA | NA | NA | NA | 1 |
| 1 | rs6588025 | FOXD3 | 63563091 | C | T | 50.6 | 101.2 | 0.50 | 16.89 | 3.96E-05 | NA | NA | NA | NA | 1 |
| 1 | rs10921202 | RGS1 | 190813675 | G | T | 31.0 | 6.0 | 5.17 | 16.89 | 3.96E-05 | A | C | 0.944 | 1.00 | 1 |
| 5 | rs11740064 | PRELID2 | 145112165 | C | T | 10.5 | 39.6 | 0.27 | 16.88 | 3.98E-05 | G | A | 0.242 | 0.92 | 1 |
| 4 | rs1402673 | MAML3 | 141242392 | A | G | 97.1 | 47.8 | 2.03 | 16.78 | 4.20E-05 | C | T | 0.226 | 0.96 | 1 |
| 17 | rs12600697 | RICH2 | 12677491 | C | T | 73.6 | 31.6 | 2.33 | 16.76 | 4.24E-05 | A | G | 0.214 | 0.96 | 1 |
| 8 | rs17832285 | POU5F1P1 | 128178175 | A | G | 76.9 | 33.9 | 2.27 | 16.69 | 4.39E-05 | NA | NA | NA | NA | 1 |
| 6 | rs13208443 | EPB41L2 | 131444507 | G | T | 74.4 | 32.3 | 2.31 | 16.67 | 4.44E-05 | NA | NA | NA | NA | 1 |
| 25 | rs2750171 | PABPC5 | 90378882 | G | A | 30.0 | 71.0 | 0.42 | 16.64 | 4.51E-05 | NA | NA | NA | NA | 1 |
| 25 | rs35990180 | PCDH11X PABPC5 | 90980737 | G | T | 30.0 | 71.0 | 0.42 | 16.64 | 4.51E-05 | NA | NA | NA | NA | 1 |
| 25 | rs2759867 | PCDH11X | 91199296 | G | A | 30.0 | 71.0 | 0.42 | 16.64 | 4.51E-05 | NA | NA | NA | NA | 1 |
| 25 | rs35247814 | PCDH11X PABPC5 | 91141496 | C | T | 32.0 | 74.0 | 0.43 | 16.64 | 4.52E-05 | NA | NA | NA | NA | 1 |
| 22 | rs140392 | CCDC116 UBE2L3 PI4KAP2 HIC2 LOC150223 | 19792353 | A | G | 11.7 | 41.5 | 0.28 | 16.63 | 4.53E-05 | NA | NA | NA | NA | 1 |
| 16 | rs7194361 | WWOX | 77665855 | C | T | 93.9 | 45.8 | 2.05 | 16.52 | 4.81E-05 | NA | NA | NA | NA | 1 |
| 1 | rs2294253 | TIPRL IQWD1 BRP44 GPR161 ADCY10 | 166423251 | C | T | 65.6 | 121.1 | 0.54 | 16.50 | 4.87E-05 | A | G | 0.179 | 1.04 | 1 |
| 11 | rs1108001 | NAV2 HTATIP2 DBX1 PRMT3 | 20255374 | A | G | 127.0 | 70.0 | 1.81 | 16.49 | 4.88E-05 | A | G | 0.404 | 0.98 | 1 |
| 6 | rs9358918 | HIST1H4H HIST1H2BI | 26394723 | C | T | 63.0 | 25.0 | 2.52 | 16.41 | 5.10E-05 | NA | NA | NA | NA | 1 |
| 6 | rs1974051 | OPN5 GPR115 GPR111 | 48024184 | A | G | 44.4 | 13.5 | 3.28 | 16.40 | 5.12E-05 | NA | NA | NA | NA | 1 |
| 5 | rs11738478 | ENC1 HEXB | 73595338 | C | T | 96.3 | 47.8 | 2.02 | 16.35 | 5.28E-05 | NA | NA | NA | NA | 1 |
| 25 | rs35782110 | TGIF2LX CPXCR1 | 88814628 | G | A | 31.0 | 72.0 | 0.43 | 16.32 | 5.35E-05 | NA | NA | NA | NA | 1 |
| 25 | rs35143113 | PCDH11X PABPC5 | 91686539 | T | C | 31.0 | 72.0 | 0.43 | 16.32 | 5.35E-05 | NA | NA | NA | NA | 1 |
| 6 | rs13218591 | BTN3A2 | 26484811 | C | T | 119.8 | 65.0 | 1.85 | 16.31 | 5.39E-05 | NA | NA | NA | NA | 1 |
| 1 | rs7534720 | GTF2B PKN2 CCBL2 | 88888888 | C | T | 61.9 | 115.7 | 0.54 | 16.29 | 5.44E-05 | NA | NA | NA | NA | 1 |
| 8 | rs7004358 | TOX | 60453370 | A | G | 27.4 | 66.4 | 0.41 | 16.28 | 5.47E-05 | T | C | 0.379 | 0.97 | 1 |
| 17 | rs9906003 | KIF19 RPL38 DNAI2 TTYH2 | 69576859 | A | G | 84.3 | 39.4 | 2.14 | 15.80 | 5.53E-05 | *C* | *T* | *0.024* | 1.08 | 1 |
| 2 | rs6741418 | STAT1 GLS STAT4 | 191431964 | C | T | 68.0 | 28.4 | 2.39 | 15.43 | 5.62E-05 | NA | NA | NA | NA | 1 |
| 9 | rs406058 | LOC100506422 C9orf82 | 26361680 | A | G | 52.2 | 18.4 | 2.84 | 16.22 | 5.65E-05 | NA | NA | NA | NA | 1 |
| 19 | rs6510761 | HMG20B C19orf28 | 3516909 | C | T | 22.2 | 58.3 | 0.38 | 16.22 | 5.65E-05 | C | T | 0.886 | 0.99 | 1 |
| 17 | rs12942418 | KIF19 RPL38 DNAI2 TTYH2 | 69586313 | A | G | 31.4 | 72.4 | 0.43 | 16.20 | 5.71E-05 | A | G | 0.610 | 0.98 | 1 |
| 10 | rs11193120 | SORCS1 | 108678768 | A | G | 68.9 | 125.0 | 0.55 | 16.19 | 5.72E-05 | NA | NA | NA | NA | 1 |
| 2 | rs13007523 | DPP10 | 115338669 | A | G | 75.0 | 133.0 | 0.56 | 16.17 | 5.78E-05 | T | C | 0.418 | 0.98 | 1 |
| 6 | rs7756191 | DNAH8 | 38865356 | C | T | 129.0 | 72.0 | 1.79 | 16.16 | 5.81E-05 | NA | NA | NA | NA | 1 |
| 1 | rs944775 | RASSF5 IKBKE | 204735216 | C | T | 125.2 | 69.1 | 1.81 | 16.16 | 5.81E-05 | C | T | 0.250 | 0.97 | 1 |
| 7 | rs10259988 | DPP6 | 154139158 | A | G | 28.1 | 67.3 | 0.42 | 16.14 | 5.87E-05 | T | C | 0.931 | 1.00 | 1 |
| 4 | rs1460160 | PCDH18 LOC641364 | 137854324 | A | G | 52.2 | 102.1 | 0.51 | 16.13 | 5.92E-05 | NA | NA | NA | NA | 1 |
| 1 | rs855314 | ALG6 PGM1 EFCAB7 ITGB3BP DLEU2L | 63867699 | A | G | 75.8 | 33.7 | 2.25 | 16.12 | 5.94E-05 | G | A | 0.771 | 0.99 | 1 |
| 22 | rs1296826 | BID BCL2L13 SLC25A18 ATP6V1E1 | 16459518 | C | T | 7.4 | 32.9 | 0.23 | 16.07 | 6.11E-05 | C | T | 0.132 | 1.10 | 1 |
| 1 | rs12145826 | MRPL20 B3GALT6 UBE2J2 CALML6 CCNL2 SDF4 TNFRSF18 GLTPD1 SLC35E2 C1orf159 ATAD3B CENTB5 ATAD3C TAS1R3 ATAD3A MXRA8 SCNN1D SSU72 TMEM52 RP11-345P4.4 MIB2 PUSL1 CPSF3L TTLL10 NADK MMP23B MMP23A FAM132A DVL1 AURKAIP1 CDC2L1 VWA1 GNB1 TNFRSF4 | 1055892 | A | G | 20.8 | 55.9 | 0.37 | 16.06 | 6.14E-05 | A | G | 0.365 | 0.95 | 1 |
| 2 | rs10197319 | ICOS CTLA4 | 204471289 | A | G | 138.0 | 79.0 | 1.75 | 16.04 | 6.20E-05 | A | G | 0.666 | 1.01 | 1 |
| 1 | rs12144971 | DUSP10 | 220099108 | C | T | 118.0 | 64.0 | 1.84 | 16.02 | 6.26E-05 | C | T | 0.780 | 1.01 | 1 |
| 1 | rs4240931 | DUSP10 | 220105678 | C | T | 64.0 | 118.0 | 0.54 | 16.02 | 6.26E-05 | A | G | 0.941 | 1.00 | 1 |
| 25 | rs35901630 | PCDH11X PABPC5 | 90834115 | A | C | 32.0 | 73.0 | 0.44 | 16.01 | 6.30E-05 | NA | NA | NA | NA | 1 |
| 25 | rs34340287 | PCDH11X | 91456478 | C | T | 32.0 | 73.0 | 0.44 | 16.01 | 6.30E-05 | NA | NA | NA | NA | 1 |
| 25 | rs35128588 | PCDH11X PABPC5 | 91683943 | A | G | 32.0 | 73.0 | 0.44 | 16.01 | 6.30E-05 | NA | NA | NA | NA | 1 |
| 10 | rs3781514 | GFRA1 | 117851287 | A | G | 31.5 | 72.3 | 0.44 | 16.00 | 6.33E-05 | NA | NA | NA | NA | 1 |
| 25 | rs2578795 | PCDH11X PABPC5 | 91573045 | G | A | 30.0 | 70.0 | 0.43 | 16.00 | 6.33E-05 | NA | NA | NA | NA | 1 |
| 2 | rs13424546 | ARL6IP2 GEMIN6 GALM SFRS7 HNRPLL | 38576553 | A | G | 96.0 | 48.0 | 2.00 | 16.00 | 6.33E-05 | T | C | 0.613 | 1.02 | 1 |
| 20 | rs157649 | DOK5 | 52853993 | A | G | 70.0 | 126.0 | 0.56 | 16.00 | 6.33E-05 | G | A | 0.898 | 1.00 | 1 |
| 2 | rs7607760 | ICOS CTLA4 | 204473439 | G | T | 79.7 | 138.9 | 0.57 | 16.00 | 6.34E-05 | NA | NA | NA | NA | 1 |
| 7 | rs4960602 | DPP6 | 154139221 | A | G | 30.5 | 70.8 | 0.43 | 15.99 | 6.37E-05 | T | C | 0.992 | 1.00 | 1 |
| 9 | rs7041639 | MOBKL2B | 27331338 | A | G | 40.4 | 11.6 | 3.48 | 15.96 | 6.48E-05 | NA | NA | NA | NA | 1 |
| 18 | rs17679032 | BCL2 | 59008140 | C | T | 7.4 | 32.6 | 0.23 | 15.93 | 6.58E-05 | G | A | 0.562 | 0.97 | 1 |
| 10 | rs10884387 | SORCS1 | 108682142 | C | T | 62.0 | 115.0 | 0.54 | 15.87 | 6.78E-05 | T | C | 0.962 | 1.00 | 1 |
| 1 | rs15672 | RASSF5 IKBKE | 204736581 | A | G | 125.8 | 70.1 | 1.80 | 15.86 | 6.81E-05 | NA | NA | NA | NA | 1 |
| 14 | rs4902468 | PLEK2 C14orf83 | 66969020 | C | T | 38.3 | 10.5 | 3.63 | 15.79 | 7.06E-05 | NA | NA | NA | NA | 1 |
| 1 | rs10800495 | KIFAP3 SELL SELE C1orf156 C1orf112 SCYL3 | 168264824 | C | T | 39.1 | 83.0 | 0.47 | 15.76 | 7.18E-05 | NA | NA | NA | NA | 1 |
| 10 | rs10884381 | SORCS1 | 108676055 | C | T | 121.7 | 67.2 | 1.81 | 15.73 | 7.30E-05 | G | A | 0.944 | 1.00 | 1 |
| 10 | rs17121941 | SORCS1 | 108788082 | A | G | 61.5 | 24.7 | 2.49 | 15.72 | 7.34E-05 | NA | NA | NA | NA | 1 |
| 25 | rs2652930 | TGIF2LX | 88689517 | C | T | 33.0 | 74.0 | 0.45 | 15.71 | 7.38E-05 | NA | NA | NA | NA | 1 |
| 25 | rs3131236 | PABPC5 PCDH11X | 90676992 | T | C | 33.0 | 74.0 | 0.45 | 15.71 | 7.38E-05 | NA | NA | NA | NA | 1 |
| 25 | rs2628508 | PCDH11X PABPC5 | 90889044 | G | A | 33.0 | 74.0 | 0.45 | 15.71 | 7.38E-05 | NA | NA | NA | NA | 1 |
| 14 | rs724611 | KCNK10 | 87796780 | A | G | 35.7 | 77.8 | 0.46 | 15.69 | 7.47E-05 | NA | NA | NA | NA | 1 |
| 25 | rs35842692 | PCDH11X PABPC5 | 90987933 | C | A | 31.0 | 71.0 | 0.44 | 15.69 | 7.48E-05 | NA | NA | NA | NA | 1 |
| 25 | rs2522574 | PCDH11X | 91433446 | C | T | 31.0 | 71.0 | 0.44 | 15.69 | 7.48E-05 | NA | NA | NA | NA | 1 |
| 4 | rs17433092 | UNC5C | 96445632 | C | T | 68.0 | 29.0 | 2.34 | 15.68 | 7.50E-05 | A | G | 0.193 | 0.95 | 1 |
| 2 | rs4234102 | SNED1 | 241617652 | A | G | 128.0 | 72.0 | 1.78 | 15.68 | 7.50E-05 | T | C | 0.543 | 0.98 | 1 |
| 18 | rs2878722 | RIT2 | 38437616 | A | G | 90.4 | 44.5 | 2.03 | 15.68 | 7.51E-05 | G | A | 0.959 | 1.00 | 1 |
| 14 | rs2415836 | FSCB C14orf28 | 43610310 | A | C | 37.2 | 80.1 | 0.46 | 15.68 | 7.52E-05 | A | C | 0.194 | 0.95 | 1 |
| 1 | rs284227 | LPHN2 | 82152034 | C | T | 47.0 | 94.0 | 0.50 | 15.67 | 7.55E-05 | G | A | 0.577 | 1.02 | 1 |
| 1 | rs9438724 | LPHN2 | 82210736 | A | C | 95.5 | 48.1 | 1.99 | 15.66 | 7.56E-05 | NA | NA | NA | NA | 1 |
| 1 | rs385367 | LPHN2 | 82187787 | A | G | 96.2 | 48.6 | 1.98 | 15.64 | 7.65E-05 | C | T | 0.695 | 1.01 | 1 |
| 3 | rs1385331 | LRRC33 | 197835612 | C | T | 82.0 | 141.0 | 0.58 | 15.61 | 7.78E-05 | A | G | 0.233 | 1.03 | 1 |
| 13 | rs2311185 | SGCG | 22514490 | A | G | 61.4 | 113.6 | 0.54 | 15.60 | 7.81E-05 | NA | NA | NA | NA | 1 |
| 13 | rs7983584 | KLF12 | 73832798 | G | T | 13.3 | 42.8 | 0.31 | 15.59 | 7.85E-05 | NA | NA | NA | NA | 1 |
| 9 | rs12338283 | GNA14 VPS13A | 78992806 | A | G | 83.0 | 39.4 | 2.11 | 15.56 | 7.98E-05 | NA | NA | NA | NA | 1 |
| 13 | rs2810134 | DACH1 | 71395250 | A | G | 54.4 | 104.0 | 0.52 | 15.56 | 7.99E-05 | NA | NA | NA | NA | 1 |
| 12 | rs2121889 | RASSF9 MGAT4C NTS | 84805288 | C | T | 45.0 | 91.0 | 0.49 | 15.56 | 8.00E-05 | T | C | 0.069 | 0.93 | 1 |
| 5 | rs10512743 | LOC340094 ADAMTS16 | 4686984 | A | G | 10.0 | 37.0 | 0.27 | 15.51 | 8.20E-05 | A | G | 0.750 | 0.98 | 1 |
| 1 | rs1040404 | TIPRL IQWD1 BRP44 GPR161 ADCY10 | 166426514 | A | G | 120.2 | 66.4 | 1.81 | 15.50 | 8.24E-05 | A | G | 0.295 | 1.03 | 1 |
| 7 | rs7795581 | ACTR3B | 152226785 | C | T | 37.0 | 79.5 | 0.47 | 15.50 | 8.26E-05 | G | A | 0.348 | 0.96 | 1 |
| 9 | rs11145347 | GNA14 VPS13A | 79036336 | C | T | 80.0 | 37.4 | 2.14 | 15.48 | 8.32E-05 | NA | NA | NA | NA | 1 |
| 14 | rs6572235 | FSCB C14orf28 | 43588964 | A | G | 37.0 | 79.4 | 0.47 | 15.47 | 8.36E-05 | NA | NA | NA | NA | 1 |
| 9 | rs7025532 | GNA14 VPS13A | 79144365 | C | T | 80.0 | 37.4 | 2.14 | 15.47 | 8.36E-05 | NA | NA | NA | NA | 1 |
| 12 | rs11054854 | LOH12CR1 | 12412599 | G | T | 20.7 | 54.8 | 0.38 | 15.47 | 8.37E-05 | C | A | 0.215 | 0.94 | 1 |
| 9 | rs9411216 | AGPAT2 | 138695308 | A | G | 37.1 | 79.5 | 0.47 | 15.47 | 8.39E-05 | A | G | 0.235 | 0.95 | 1 |
| 6 | rs7745052 | FBXL4 C6orf168 USP45 COQ3 POU3F2 SFRS18 | 99747331 | A | G | 88.0 | 43.0 | 2.05 | 15.46 | 8.44E-05 | A | G | 0.176 | 1.05 | 1 |
| 4 | rs554399 | POLN NAT8L | 2108845 | A | G | 22.2 | 2.6 | 8.50 | 15.45 | 8.49E-05 | NA | NA | NA | NA | 1 |
| 9 | rs7030802 | GNA14 VPS13A | 79081057 | G | T | 84.0 | 40.3 | 2.09 | 15.41 | 8.65E-05 | G | T | 0.724 | 1.01 | 1 |
| 22 | rs1296820 | BID BCL2L13 SLC25A18 ATP6V1E1 | 16457720 | G | T | 29.4 | 6.0 | 4.88 | 15.40 | 8.68E-05 | NA | NA | NA | NA | 1 |
| 25 | rs2752258 | TGIF2LX | 89358109 | T | C | 32.0 | 72.0 | 0.44 | 15.38 | 8.77E-05 | NA | NA | NA | NA | 1 |
| 25 | rs2565974 | PABPC5 TGIF2LX | 89854007 | A | G | 32.0 | 72.0 | 0.44 | 15.38 | 8.77E-05 | NA | NA | NA | NA | 1 |
| 25 | rs2563370 | PCDH11X PABPC5 | 91014642 | T | C | 32.0 | 72.0 | 0.44 | 15.38 | 8.77E-05 | NA | NA | NA | NA | 1 |
| 25 | rs2750912 | PCDH11X PABPC5 | 91657372 | G | A | 32.0 | 72.0 | 0.44 | 15.38 | 8.77E-05 | NA | NA | NA | NA | 1 |
| 25 | rs34606691 | NAP1L3 FAM133A | 92084091 | G | A | 32.0 | 72.0 | 0.44 | 15.38 | 8.77E-05 | NA | NA | NA | NA | 1 |
| 9 | rs10965086 | SMARCA2 | 2162235 | A | G | 64.3 | 26.9 | 2.39 | 15.36 | 8.87E-05 | *G* | *A* | *0.005* | 0.88 | 1 |
| 5 | rs30810 | LOC643401 CDH9 | 27986240 | C | T | 12.8 | 41.8 | 0.31 | 15.35 | 8.95E-05 | C | T | 0.877 | 0.99 | 1 |
| 23 | rs11091412 | PAGE3 PAGE5 MIR4536-1 MAGEH1 MTRNR2L10 | 55378662 | T | C | 19.0 | 52.0 | 0.37 | 15.34 | 8.99E-05 | T | C | 0.698 | 0.99 | 1 |
| 15 | rs12900227 | RYR3 | 31610261 | C | T | 48.2 | 95.0 | 0.51 | 15.33 | 9.01E-05 | NA | NA | NA | NA | 1 |
| 15 | rs2292548 | FMN1 RYR3 | 31315370 | C | T | 10.1 | 36.9 | 0.27 | 15.32 | 9.05E-05 | G | A | 0.320 | 0.94 | 1 |
| 2 | rs12616245 | ICOS CTLA4 | 204479048 | A | G | 81.0 | 139.1 | 0.58 | 15.30 | 9.16E-05 | G | A | 0.567 | 1.02 | 1 |
| 4 | rs7682075 | CXCL13 CCNG2 CCNI CNOT6L 41163 | 78546357 | A | G | 20.2 | 1.8 | 10.98 | 15.30 | 9.17E-05 | NA | NA | NA | NA | 1 |
| 10 | rs11194147 | SORCS1 | 110276139 | C | T | 13.0 | 42.0 | 0.31 | 15.29 | 9.22E-05 | A | G | 0.665 | 0.98 | 1 |
| 6 | rs10946659 | DCDC2 NRSN1 | 24150372 | G | T | 82.0 | 39.0 | 2.10 | 15.28 | 9.26E-05 | NA | NA | NA | NA | 1 |
| 9 | rs10491843 | GNA14 VPS13A | 79170794 | A | C | 20.3 | 54.0 | 0.38 | 15.28 | 9.28E-05 | C | A | 0.624 | 0.98 | 1 |
| 9 | rs17423984 | GNA14 VPS13A | 79126235 | A | G | 20.3 | 54.0 | 0.38 | 15.25 | 9.42E-05 | NA | NA | NA | NA | 1 |
| 23 | rs5919529 | OPHN1 YIPF6 | 67274933 | A | G | 33.0 | 8.0 | 4.13 | 15.24 | 9.45E-05 | T | C | 0.750 | 1.01 | 1 |
| 10 | rs822095 | SORCS1 | 108699185 | A | G | 110.1 | 59.3 | 1.86 | 15.24 | 9.46E-05 | A | G | 0.900 | 1.00 | 1 |
| 1 | rs12123693 | KIFAP3 SELL SELE C1orf156 C1orf112 SCYL3 | 168251948 | C | T | 38.8 | 81.7 | 0.48 | 15.24 | 9.46E-05 | A | G | 0.603 | 1.02 | 1 |
| 11 | rs226129 | ARHGAP20 | 110208877 | C | T | 27.2 | 64.5 | 0.42 | 15.23 | 9.52E-05 | G | A | 0.139 | 1.05 | 1 |
| 15 | rs10083673 | MYO5A | 50497632 | A | G | 111.0 | 60.0 | 1.85 | 15.21 | 9.62E-05 | *G* | *A* | *0.006* | 1.09 | 1 |
| 1 | rs12146137 | KIFAP3 SELL SELE C1orf156 C1orf112 SCYL3 | 168304248 | C | T | 37.0 | 79.0 | 0.47 | 15.21 | 9.64E-05 | T | C | 0.609 | 1.02 | 1 |
| 1 | rs10494080 | VAV3 | 108203598 | A | C | 115.0 | 63.0 | 1.83 | 15.19 | 9.72E-05 | T | G | 0.666 | 0.99 | 1 |
| 1 | rs10924987 | VN1R5 ZNF496 LOC441931 | 245557766 | C | T | 94.5 | 48.0 | 1.97 | 15.15 | 9.91E-05 | NA | NA | NA | NA | 1 |
| 9 | rs451664 | LOC100506422 C9orf82 | 26360564 | A | G | 56.4 | 21.9 | 2.57 | 15.14 | 9.97E-05 | C | T | 0.959 | 1.00 | 1 |
| 1 | rs1885020 | FAM5B | 175717832 | C | T | 62.2 | 25.7 | 2.42 | 15.12 | 1.01E-04 | A | G | 0.695 | 1.01 | 1 |
| 25 | rs34640393 | PCDH11X PABPC5 | 91106535 | T | C | 33.0 | 73.0 | 0.45 | 15.09 | 1.02E-04 | NA | NA | NA | NA | 1 |
| 25 | rs2563232 | PCDH11X | 91152566 | A | G | 33.0 | 73.0 | 0.45 | 15.09 | 1.02E-04 | NA | NA | NA | NA | 1 |
| 25 | rs34265393 | PCDH11X PABPC5 | 91628316 | G | A | 33.0 | 73.0 | 0.45 | 15.09 | 1.02E-04 | NA | NA | NA | NA | 1 |
| 25 | rs34851597 | NAP1L3 FAM133A | 92178420 | T | C | 33.0 | 73.0 | 0.45 | 15.09 | 1.02E-04 | NA | NA | NA | NA | 1 |
| 9 | rs4012480 | GNA14 VPS13A | 78984573 | G | T | 39.3 | 82.1 | 0.48 | 15.09 | 1.02E-04 | T | G | 0.716 | 1.01 | 1 |
| 17 | rs1171208 | CD300C CD300LB | 70014950 | A | G | 30.5 | 69.4 | 0.44 | 15.09 | 1.03E-04 | NA | NA | NA | NA | 1 |
| 9 | rs10115162 | GNA14 VPS13A | 79052322 | C | T | 82.0 | 39.3 | 2.09 | 15.08 | 1.03E-04 | G | A | 0.727 | 1.01 | 1 |
| 4 | rs10025768 | PPP3CA | 102184318 | A | G | 12.0 | 40.0 | 0.30 | 15.08 | 1.03E-04 | T | C | 0.444 | 0.95 | 1 |
| 22 | rs5996879 | ADRBK2 CRYBB2 CRYBB3 | 23998730 | A | C | 27.7 | 65.1 | 0.43 | 15.07 | 1.03E-04 | NA | NA | NA | NA | 1 |
| 25 | rs34438890 | TGIF2LX PABPC5 | 89600891 | A | G | 31.0 | 70.0 | 0.44 | 15.06 | 1.04E-04 | NA | NA | NA | NA | 1 |
| 25 | rs34362036 | PCDH11X | 91107226 | A | C | 31.0 | 70.0 | 0.44 | 15.06 | 1.04E-04 | NA | NA | NA | NA | 1 |
| 15 | rs17786183 | SNRPN SNURF PAR-SN PAR1 PAR5 | 22792884 | C | T | 50.0 | 18.0 | 2.78 | 15.06 | 1.04E-04 | T | C | 0.319 | 1.05 | 1 |
| 6 | rs9396802 | NUP153 KIF13A | 17863863 | C | T | 128.0 | 73.0 | 1.75 | 15.05 | 1.05E-04 | T | C | 0.582 | 0.98 | 1 |
| 22 | rs7510924 | LL22NC03-75B3.6 LL22NC03-75B3.6 | 43039988 | C | T | 59.9 | 24.3 | 2.46 | 15.05 | 1.05E-04 | NA | NA | NA | NA | 1 |
| 12 | rs2029774 | CPM | 67587554 | C | T | 13.2 | 42.0 | 0.31 | 14.98 | 1.09E-04 | NA | NA | NA | NA | 1 |
| 20 | rs6038644 | BMP2 | 6841128 | A | G | 13.6 | 42.6 | 0.32 | 14.98 | 1.09E-04 | A | G | 0.338 | 0.95 | 1 |
| 12 | rs11104365 | MGAT4C MKRN9P | 86164650 | C | T | 80.0 | 137.0 | 0.58 | 14.97 | 1.09E-04 | C | T | 0.055 | 1.06 | 1 |
| 9 | rs7862396 | SMARCA2 | 1868648 | C | T | 21.2 | 55.0 | 0.39 | 14.96 | 1.10E-04 | NA | NA | NA | NA | 1 |
| 2 | rs11884879 | SPP2 TRPM8 | 234742933 | A | G | 32.6 | 72.2 | 0.45 | 14.95 | 1.11E-04 | NA | NA | NA | NA | 1 |
| 10 | rs12415204 | GPR120 CEP55 | 95320880 | A | C | 40.7 | 83.9 | 0.49 | 14.94 | 1.11E-04 | A | C | 0.295 | 0.97 | 1 |
| 6 | rs10947963 | NCR2 | 41413954 | A | G | 105.0 | 56.0 | 1.88 | 14.91 | 1.13E-04 | T | C | 0.547 | 0.98 | 1 |
| 11 | rs12293750 | WT1 | 32403561 | A | C | 14.8 | 44.5 | 0.33 | 14.91 | 1.13E-04 | A | C | 0.292 | 1.12 | 1 |
| 11 | rs10437584 | NELL1 | 21313973 | G | T | 101.0 | 53.1 | 1.90 | 14.89 | 1.14E-04 | T | G | 0.167 | 0.96 | 1 |
| 13 | rs17080439 | GJA3 GJB6 IFT88 GJB2 CRYL1 ZMYM2 | 19810067 | A | G | 11.0 | 38.0 | 0.29 | 14.88 | 1.15E-04 | G | A | 0.621 | 1.03 | 1 |
| 4 | rs11940562 | PCDH7 | 32236847 | C | T | 84.5 | 41.3 | 2.05 | 14.86 | 1.16E-04 | T | C | 0.071 | 0.94 | 1 |
| 3 | rs13433781 | THRB | 24757705 | C | T | 63.4 | 26.8 | 2.37 | 14.86 | 1.16E-04 | NA | NA | NA | NA | 1 |
| 1 | rs3860295 | RASSF5 IKBKE | 204742954 | A | G | 117.0 | 65.0 | 1.80 | 14.86 | 1.16E-04 | T | C | 0.251 | 0.97 | 1 |
| 15 | rs3743157 | PDE8A AKAP13 | 83481536 | A | C | 34.0 | 74.0 | 0.46 | 14.81 | 1.19E-04 | A | C | 0.402 | 1.03 | 1 |
| 25 | rs2915713 | TGIF2LX | 88800743 | C | G | 41.0 | 84.0 | 0.49 | 14.79 | 1.20E-04 | NA | NA | NA | NA | 1 |
| 25 | rs2984684 | TGIF2LX | 88826971 | A | C | 41.0 | 84.0 | 0.49 | 14.79 | 1.20E-04 | NA | NA | NA | NA | 1 |
| 16 | rs204034 | SHISA9 | 13230842 | A | G | 84.0 | 41.0 | 2.05 | 14.79 | 1.20E-04 | A | G | 0.312 | 1.04 | 1 |
| 11 | rs10840025 | LMO1 TUB STK33 RIC3 | 8305301 | C | T | 74.3 | 34.2 | 2.17 | 14.79 | 1.20E-04 | NA | NA | NA | NA | 1 |
| 5 | rs13155834 | ERGIC1 | 172216536 | A | G | 56.6 | 22.4 | 2.52 | 14.79 | 1.20E-04 | NA | NA | NA | NA | 1 |
| 1 | rs4612651 | VAV3 | 108157816 | C | T | 128.8 | 74.0 | 1.74 | 14.78 | 1.21E-04 | NA | NA | NA | NA | 1 |
| 25 | rs35193207 | XG XGPY2 CD99 GYG2 LINC00102 | 2675647 | G | A | 32.0 | 71.0 | 0.45 | 14.77 | 1.22E-04 | NA | NA | NA | NA | 1 |
| 25 | rs35415285 | TGIF2LX PABPC5 | 89460911 | C | T | 32.0 | 71.0 | 0.45 | 14.77 | 1.22E-04 | NA | NA | NA | NA | 1 |
| 25 | rs34291104 | TGIF2LX PABPC5 | 89600981 | C | T | 32.0 | 71.0 | 0.45 | 14.77 | 1.22E-04 | NA | NA | NA | NA | 1 |
| 25 | rs3126717 | PABPC5 PCDH11X | 90709670 | T | C | 32.0 | 71.0 | 0.45 | 14.77 | 1.22E-04 | NA | NA | NA | NA | 1 |
| 25 | rs3100325 | PCDH11X PABPC5 | 90801801 | T | C | 32.0 | 71.0 | 0.45 | 14.77 | 1.22E-04 | NA | NA | NA | NA | 1 |
| 25 | rs2556924 | PCDH11X NAP1L3 | 91764568 | C | T | 32.0 | 71.0 | 0.45 | 14.77 | 1.22E-04 | NA | NA | NA | NA | 1 |
| 25 | rs34353711 | NAP1L3 FAM133A | 91959375 | G | A | 32.0 | 71.0 | 0.45 | 14.77 | 1.22E-04 | NA | NA | NA | NA | 1 |
| 25 | rs34464858 | NAP1L3 FAM133A | 92129750 | C | A | 32.0 | 71.0 | 0.45 | 14.77 | 1.22E-04 | NA | NA | NA | NA | 1 |
| 12 | rs3990897 | MKRN9P MGAT4C | 86251725 | C | T | 130.0 | 75.0 | 1.73 | 14.76 | 1.22E-04 | C | T | 0.160 | 0.96 | 1 |
| 22 | rs3814997 | PRAME GGTL4 ZNF280A ZNF280B | 21365233 | C | T | 29.0 | 66.6 | 0.44 | 14.75 | 1.23E-04 | *G* | *A* | *0.010* | 0.91 | 1 |
| 25 | rs35520738 | PCDH11X PABPC5 | 90969699 | A | G | 30.0 | 68.0 | 0.44 | 14.73 | 1.24E-04 | NA | NA | NA | NA | 1 |
| 11 | rs4148621 | KCNJ11 ABCC8 B7H6 USH1C NUCB2 | 17408003 | A | C | 30.0 | 68.0 | 0.44 | 14.73 | 1.24E-04 | T | G | 0.666 | 1.02 | 1 |
| 10 | rs4748417 | TMEM236 MRC1 STAM MIR511-2 MIR511-1 | 17819812 | C | T | 2.0 | 20.0 | 0.10 | 14.73 | 1.24E-04 | NA | NA | NA | NA | 1 |
| 25 | rs28861531 | CSF2RA MIR3690 CRLF2 IL3RA SLC25A6 | 1334728 | G | C | 10.0 | 36.0 | 0.28 | 14.70 | 1.26E-04 | NA | NA | NA | NA | 1 |
| 1 | rs2235207 | TIPRL IQWD1 BRP44 GPR161 ADCY10 | 166425886 | C | T | 66.0 | 118.0 | 0.56 | 14.70 | 1.26E-04 | T | C | 0.321 | 1.03 | 1 |
| 1 | rs16827018 | POLR3GL RBM8A ITGA10 SEC22B GNRHR2 LIX1L NBPF20 PEX11B NOTCH2NL TXNIP ANKRD34A ANKRD35 HFE2 | 144256582 | G | T | 5.8 | 28.0 | 0.21 | 14.69 | 1.27E-04 | C | A | 0.184 | 0.92 | 1 |
| 22 | rs6003222 | PRAME GGTL4 ZNF280A ZNF280B | 21361579 | C | T | 27.8 | 64.6 | 0.43 | 14.68 | 1.27E-04 | C | T | 0.144 | 0.94 | 1 |
| 1 | rs11803212 | SIPA1L2 DISC1 | 230481716 | A | G | 12.2 | 39.9 | 0.31 | 14.66 | 1.29E-04 | A | G | 0.167 | 1.08 | 1 |
| 9 | rs13296370 | MLLT3 | 20336912 | C | T | 31.9 | 70.6 | 0.45 | 14.66 | 1.29E-04 | C | T | 0.578 | 1.03 | 1 |
| 10 | rs7921647 | C10orf88 FAM24B CUZD1 | 124661645 | C | T | 74.9 | 129.6 | 0.58 | 14.65 | 1.30E-04 | NA | NA | NA | NA | 1 |
| 18 | rs2194633 | NETO1 | 68895144 | A | G | 86.0 | 144.0 | 0.60 | 14.63 | 1.31E-04 | G | A | 0.439 | 0.98 | 1 |
| 4 | rs2555639 | HPGD | 175698105 | C | T | 120.2 | 67.8 | 1.77 | 14.61 | 1.32E-04 | NA | NA | NA | NA | 1 |
| 4 | rs6854845 | BTC | 75965689 | G | T | 62.6 | 26.6 | 2.36 | 14.58 | 1.34E-04 | NA | NA | NA | NA | 1 |
| 22 | rs390495 | MICAL3 | 16884801 | G | T | 73.0 | 127.0 | 0.57 | 14.58 | 1.34E-04 | T | G | 0.588 | 1.01 | 1 |
| 17 | rs4254372 | WSCD1 | 5964062 | A | G | 45.9 | 15.9 | 2.89 | 14.57 | 1.35E-04 | NA | NA | NA | NA | 1 |
| 9 | rs7033598 | IFNE1 IFNA6 IFNA5 IFNA1 MTAP IFNA13 IFNA8 KLHL9 IFNA2 | 21555674 | C | T | 140.0 | 83.0 | 1.69 | 14.57 | 1.35E-04 | G | A | 0.696 | 1.01 | 1 |
| 6 | rs12213754 | LAMA2 | 129481688 | A | G | 67.5 | 29.9 | 2.26 | 14.57 | 1.35E-04 | C | T | 0.560 | 0.96 | 1 |
| 11 | rs175131 | VPS37C ZP1 CD6 SLC15A3 CD5 TMEM132A PGA5 PGA4 VWCE GPR44 DDB1 PRPF19 CCDC86 DAK PGA3 TMEM109 | 60593095 | A | G | 16.0 | 46.0 | 0.35 | 14.55 | 1.37E-04 | NA | NA | NA | NA | 1 |
| 18 | rs1917917 | EPB41L3 TTMA L3MBTL4 | 5743004 | C | T | 67.0 | 119.0 | 0.56 | 14.54 | 1.37E-04 | C | T | 0.838 | 0.99 | 1 |
| 14 | rs17107326 | NRXN3 | 77851748 | C | T | 34.0 | 9.0 | 3.78 | 14.53 | 1.38E-04 | G | A | 0.656 | 1.03 | 1 |
| 3 | rs571879 | APPL1 HESX1 IL17RD DNHD2 ASB14 | 57322918 | C | T | 115.0 | 64.0 | 1.80 | 14.53 | 1.38E-04 | A | G | 0.589 | 1.02 | 1 |
| 19 | rs9676750 | ONECUT3 | 1648633 | A | G | 65.5 | 28.6 | 2.29 | 14.52 | 1.39E-04 | NA | NA | NA | NA | 1 |
| 2 | rs7590305 | FABP1 THNSL2 | 88294246 | C | T | 80.3 | 136.3 | 0.59 | 14.51 | 1.39E-04 | C | T | 0.147 | 0.96 | 1 |
| 2 | rs13000828 | DPP10 | 115354760 | A | G | 75.0 | 129.4 | 0.58 | 14.50 | 1.40E-04 | T | C | 0.269 | 0.97 | 1 |
| 10 | rs822076 | SORCS1 | 108688499 | C | T | 61.1 | 111.1 | 0.55 | 14.49 | 1.41E-04 | NA | NA | NA | NA | 1 |
| 9 | rs10987191 | FAM125B PBX3 | 127996989 | A | C | 7.1 | 30.4 | 0.23 | 14.49 | 1.41E-04 | A | C | 0.871 | 0.99 | 1 |
| 25 | rs2558928 | CPXCR1 | 88365332 | G | A | 33.0 | 72.0 | 0.46 | 14.49 | 1.41E-04 | NA | NA | NA | NA | 1 |
| 25 | rs2498121 | TGIF2LX | 88871191 | A | G | 33.0 | 72.0 | 0.46 | 14.49 | 1.41E-04 | NA | NA | NA | NA | 1 |
| 25 | rs2496945 | TGIF2LX | 89064027 | G | A | 33.0 | 72.0 | 0.46 | 14.49 | 1.41E-04 | NA | NA | NA | NA | 1 |
| 25 | rs2574622 | TGIF2LX | 89381724 | A | G | 33.0 | 72.0 | 0.46 | 14.49 | 1.41E-04 | NA | NA | NA | NA | 1 |
| 25 | rs2557523 | TGIF2LX PABPC5 | 89655381 | C | T | 33.0 | 72.0 | 0.46 | 14.49 | 1.41E-04 | NA | NA | NA | NA | 1 |
| 25 | rs2435461 | PABPC5 TGIF2LX | 89934433 | G | A | 33.0 | 72.0 | 0.46 | 14.49 | 1.41E-04 | NA | NA | NA | NA | 1 |
| 25 | rs3096944 | PABPC5 PCDH11X | 90676709 | C | T | 33.0 | 72.0 | 0.46 | 14.49 | 1.41E-04 | NA | NA | NA | NA | 1 |
| 25 | rs3100571 | PCDH11X PABPC5 | 90785483 | G | A | 33.0 | 72.0 | 0.46 | 14.49 | 1.41E-04 | NA | NA | NA | NA | 1 |
| 25 | rs34502698 | PCDH11X PABPC5 | 90919442 | T | C | 33.0 | 72.0 | 0.46 | 14.49 | 1.41E-04 | NA | NA | NA | NA | 1 |
| 25 | rs2573893 | PCDH11X PABPC5 | 91541995 | T | A | 33.0 | 72.0 | 0.46 | 14.49 | 1.41E-04 | NA | NA | NA | NA | 1 |
| 25 | rs35769768 | PCDH11X NAP1L3 | 91825392 | C | T | 33.0 | 72.0 | 0.46 | 14.49 | 1.41E-04 | NA | NA | NA | NA | 1 |
| 25 | rs2751221 | NAP1L3 FAM133A | 91898712 | C | T | 33.0 | 72.0 | 0.46 | 14.49 | 1.41E-04 | NA | NA | NA | NA | 1 |
| 25 | rs35089013 | NAP1L3 FAM133A | 92110088 | C | T | 33.0 | 72.0 | 0.46 | 14.49 | 1.41E-04 | NA | NA | NA | NA | 1 |
| 3 | rs1357103 | LSAMP-AS3 LSAMP | 118321979 | C | T | 46.7 | 91.4 | 0.51 | 14.48 | 1.42E-04 | NA | NA | NA | NA | 1 |
| 6 | rs1889276 | RIMS1 OGFRL1 | 72386809 | C | T | 79.1 | 134.7 | 0.59 | 14.45 | 1.44E-04 | A | G | 0.546 | 1.02 | 1 |
| 10 | rs10786497 | HPSE2 CNNM1 | 100784349 | C | T | 101.0 | 53.8 | 1.88 | 14.45 | 1.44E-04 | NA | NA | NA | NA | 1 |
| 10 | rs1470379 | VIM | 17298805 | C | T | 61.2 | 111.0 | 0.55 | 14.44 | 1.45E-04 | A | G | 0.865 | 1.01 | 1 |
| 1 | rs958802 | KANK4 KANK4 L1TD1 INADL | 62496154 | C | T | 128.0 | 74.0 | 1.73 | 14.44 | 1.45E-04 | A | G | 0.458 | 1.02 | 1 |
| 10 | rs2421162 | IKZF5 C10orf88 PSTK FAM24B ACADSB CUZD1 | 124670919 | A | C | 130.4 | 75.9 | 1.72 | 14.43 | 1.46E-04 | NA | NA | NA | NA | 1 |
| 11 | rs10833507 | NELL1 SLC6A5 | 21307196 | A | G | 105.7 | 57.2 | 1.85 | 14.43 | 1.46E-04 | NA | NA | NA | NA | 1 |
| 10 | rs11248332 | IKZF5 C10orf88 PSTK FAM24B ACADSB CUZD1 | 124666325 | C | T | 130.4 | 75.9 | 1.72 | 14.42 | 1.46E-04 | NA | NA | NA | NA | 1 |
| 7 | rs817759 | NPTX2 TMEM130 BAIAP2L1 TRRAP BRI3 | 98001875 | C | T | 46.0 | 16.1 | 2.86 | 14.42 | 1.46E-04 | NA | NA | NA | NA | 1 |
| 11 | rs897111 | LMO1 TUB STK33 RIC3 | 8325991 | A | G | 60.9 | 110.7 | 0.55 | 14.41 | 1.47E-04 | A | G | 0.739 | 1.01 | 1 |
| 10 | rs7915165 | IKZF5 C10orf88 PSTK FAM24B ACADSB CUZD1 | 124650448 | A | G | 130.4 | 75.9 | 1.72 | 14.41 | 1.47E-04 | NA | NA | NA | NA | 1 |
| 25 | rs2556916 | PCDH11X PABPC5 | 91763012 | A | C | 29.0 | 66.0 | 0.44 | 14.41 | 1.47E-04 | NA | NA | NA | NA | 1 |
| 9 | rs10992463 | IARS BICD2 ECM2 ASPN CENPP OGN IPPK NOL8 OMD | 94581596 | C | T | 50.6 | 19.0 | 2.67 | 14.39 | 1.48E-04 | A | G | 0.507 | 0.97 | 1 |
| 10 | rs2435587 | PRKCQ | 6671873 | A | G | 80.9 | 39.3 | 2.06 | 14.38 | 1.49E-04 | G | A | 0.897 | 1.01 | 1 |
| 12 | rs7487519 | LIN7A | 79839379 | C | T | 110.3 | 60.7 | 1.82 | 14.38 | 1.49E-04 | NA | NA | NA | NA | 1 |
| 10 | rs243013 | VIM | 17325070 | A | C | 14.2 | 42.8 | 0.33 | 14.35 | 1.52E-04 | T | G | 0.386 | 0.95 | 1 |
| 1 | rs1024229 | MIER1 WDR78 SLC35D1 | 67285491 | G | T | 59.2 | 108.1 | 0.55 | 14.33 | 1.53E-04 | NA | NA | NA | NA | 1 |
| 1 | rs1024230 | MIER1 WDR78 SLC35D1 | 67285508 | A | C | 59.2 | 108.1 | 0.55 | 14.33 | 1.53E-04 | NA | NA | NA | NA | 1 |
| 1 | rs10493817 | GTF2B PKN2 CCBL2 | 88899475 | G | T | 68.5 | 120.6 | 0.57 | 14.33 | 1.54E-04 | G | T | 0.592 | 0.99 | 1 |
| 10 | rs3249 | VIM | 17319523 | C | T | 54.6 | 101.9 | 0.54 | 14.31 | 1.55E-04 | NA | NA | NA | NA | 1 |
| 4 | rs4693959 | HERC6 PIGY HERC5 | 89664929 | G | T | 41.2 | 13.3 | 3.10 | 14.29 | 1.57E-04 | NA | NA | NA | NA | 1 |
| 18 | rs10164078 | SETBP1 | 40409771 | A | G | 28.7 | 65.4 | 0.44 | 14.26 | 1.59E-04 | A | G | 0.250 | 0.96 | 1 |
| 5 | rs1805971 | SEMA5A | 9257142 | C | T | 66.5 | 29.5 | 2.25 | 14.25 | 1.60E-04 | A | G | 0.813 | 1.01 | 1 |
| 6 | rs9351812 | KHDRBS2 | 63232869 | C | T | 80.0 | 135.4 | 0.59 | 14.24 | 1.61E-04 | NA | NA | NA | NA | 1 |
| 3 | rs5012667 | PDZRN3 | 74041159 | C | T | 83.0 | 41.0 | 2.02 | 14.23 | 1.62E-04 | C | T | 0.564 | 0.98 | 1 |
| 5 | rs4374758 | BASP1 MYO10 | 17071543 | C | T | 68.9 | 31.2 | 2.21 | 14.22 | 1.63E-04 | NA | NA | NA | NA | 1 |
| 18 | rs583991 | CCDC102B DOK6 | 65034352 | C | T | 82.1 | 40.3 | 2.03 | 14.22 | 1.63E-04 | NA | NA | NA | NA | 1 |
| 25 | rs397334 | TGIF2LX | 89477022 | A | G | 34.0 | 73.0 | 0.47 | 14.21 | 1.63E-04 | NA | NA | NA | NA | 1 |
| 25 | rs2563565 | PCDH11X PABPC5 | 90938698 | T | C | 34.0 | 73.0 | 0.47 | 14.21 | 1.63E-04 | NA | NA | NA | NA | 1 |
| 25 | rs34081252 | PCDH11X PABPC5 | 91446708 | T | C | 34.0 | 73.0 | 0.47 | 14.21 | 1.63E-04 | NA | NA | NA | NA | 1 |
| 25 | rs2574029 | PCDH11X | 91463758 | T | C | 34.0 | 73.0 | 0.47 | 14.21 | 1.63E-04 | NA | NA | NA | NA | 1 |
| 25 | rs2750770 | PCDH11X PABPC5 | 91616010 | A | G | 34.0 | 73.0 | 0.47 | 14.21 | 1.63E-04 | NA | NA | NA | NA | 1 |
| 1 | rs12023478 | VN1R5 ZNF496 LOC441931 | 245517188 | C | T | 84.4 | 42.0 | 2.01 | 14.21 | 1.63E-04 | A | G | 0.248 | 0.97 | 1 |
| 3 | rs1873388 | CHST13 UROC1 | 127711643 | A | G | 66.0 | 117.0 | 0.56 | 14.21 | 1.63E-04 | A | G | 0.321 | 1.03 | 1 |
| 6 | rs9460965 | DCDC2 NRSN1 | 24186237 | C | T | 66.0 | 117.0 | 0.56 | 14.21 | 1.63E-04 | NA | NA | NA | NA | 1 |
| 4 | rs1350036 | MAML3 | 141051231 | C | T | 65.2 | 115.9 | 0.56 | 14.18 | 1.66E-04 | T | C | 0.204 | 0.97 | 1 |
| 13 | rs8000505 | SLITRK6 SLITRK1 | 84508545 | C | T | 75.7 | 36.0 | 2.11 | 14.16 | 1.68E-04 | NA | NA | NA | NA | 1 |
| 19 | rs12985786 | CACNA1A | 13328036 | A | G | 13.3 | 41.1 | 0.32 | 14.16 | 1.68E-04 | T | C | 0.814 | 1.02 | 1 |
| 25 | rs36009319 | TGIF2LX CPXCR1 | 88475961 | A | G | 32.0 | 70.0 | 0.46 | 14.16 | 1.68E-04 | NA | NA | NA | NA | 1 |
| 25 | rs36054522 | PCDH11X PABPC5 | 90919574 | T | C | 32.0 | 70.0 | 0.46 | 14.16 | 1.68E-04 | NA | NA | NA | NA | 1 |
| 25 | rs2524466 | PCDH11X PABPC5 | 90986953 | T | G | 32.0 | 70.0 | 0.46 | 14.16 | 1.68E-04 | NA | NA | NA | NA | 1 |
| 25 | rs35735988 | PCDH11X PABPC5 | 90993316 | A | G | 32.0 | 70.0 | 0.46 | 14.16 | 1.68E-04 | NA | NA | NA | NA | 1 |
| 25 | rs2563397 | PCDH11X PABPC5 | 91022440 | T | C | 32.0 | 70.0 | 0.46 | 14.16 | 1.68E-04 | NA | NA | NA | NA | 1 |
| 25 | rs34904303 | PCDH11X PABPC5 | 91470102 | A | G | 32.0 | 70.0 | 0.46 | 14.16 | 1.68E-04 | NA | NA | NA | NA | 1 |
| 25 | rs2522697 | PCDH11X PABPC5 | 91486463 | T | G | 32.0 | 70.0 | 0.46 | 14.16 | 1.68E-04 | NA | NA | NA | NA | 1 |
| 25 | rs35361563 | PCDH11X PABPC5 | 91571549 | A | G | 32.0 | 70.0 | 0.46 | 14.16 | 1.68E-04 | NA | NA | NA | NA | 1 |
| 9 | rs11564091 | PPP2R4 FAM73B DOLPP1 SH3GLB2 NUP188 CRAT C9orf106 | 131058327 | C | T | 57.5 | 23.6 | 2.44 | 14.15 | 1.69E-04 | T | C | 0.589 | 0.98 | 1 |
| 6 | rs6458351 | VEGFA MRPS18A POLH XPO5 C6orf206 MAD2L1BP GTPBP2 | 43899058 | C | T | 55.0 | 22.0 | 2.50 | 14.14 | 1.69E-04 | T | C | 0.462 | 1.03 | 1 |
| 22 | rs9608216 | GSTT2 GSTT1 DDT | 22587779 | C | T | 41.5 | 13.6 | 3.06 | 14.14 | 1.70E-04 | T | C | 0.922 | 1.01 | 1 |
| 2 | rs1526284 | C2orf25 LYPD6 | 150216605 | G | T | 9.6 | 34.7 | 0.28 | 14.13 | 1.71E-04 | NA | NA | NA | NA | 1 |
| 4 | rs727488 | CD38 BST1 TAPT1 FGFBP2 PROM1 CC2D2A FGFBP1 FBXL5 | 15505439 | C | T | 58.7 | 24.5 | 2.40 | 14.12 | 1.71E-04 | NA | NA | NA | NA | 1 |
| 11 | rs7950594 | NAV2 HTATIP2 DBX1 PRMT3 | 20243150 | A | G | 126.0 | 73.0 | 1.73 | 14.12 | 1.72E-04 | C | T | 0.553 | 1.02 | 1 |
| 1 | rs430600 | GTF2B PKN2 CCBL2 | 88998564 | C | T | 119.6 | 68.1 | 1.76 | 14.11 | 1.72E-04 | NA | NA | NA | NA | 1 |
| 15 | rs8033142 | WHDC1L1 PDCD6IP HERC2P2 | 20691555 | C | T | 15.2 | 44.2 | 0.34 | 14.11 | 1.73E-04 | NA | NA | NA | NA | 1 |
| 7 | rs7795713 | CARD11 GNA12 AMZ1 SDK1 TTYH3 | 2973919 | C | T | 21.4 | 2.9 | 7.36 | 14.07 | 1.76E-04 | NA | NA | NA | NA | 1 |
| 6 | rs2345981 | KHDRBS2 | 63267900 | A | G | 135.0 | 80.0 | 1.69 | 14.07 | 1.76E-04 | T | C | 0.524 | 0.98 | 1 |
| 3 | rs9865818 | LPP | 189555207 | A | G | 113.5 | 63.6 | 1.78 | 14.06 | 1.77E-04 | *G* | *A* | *0.000* | 0.81 | 1 |
| 1 | rs1415551 | KIFAP3 SELL SELE C1orf156 C1orf112 SCYL3 | 168268836 | C | T | 47.0 | 17.0 | 2.76 | 14.06 | 1.77E-04 | G | A | 0.815 | 1.01 | 1 |
| 1 | rs96501 | NTNG1 | 107820370 | C | T | 38.2 | 78.7 | 0.49 | 14.04 | 1.79E-04 | G | A | 0.194 | 0.96 | 1 |
| 5 | rs7722385 | MYO10 | 16714282 | C | T | 91.0 | 47.0 | 1.94 | 14.03 | 1.80E-04 | A | G | 0.572 | 1.02 | 1 |
| 11 | rs1478747 | LOC283177 B3GAT1 | 134274636 | A | G | 52.1 | 98.0 | 0.53 | 14.02 | 1.81E-04 | NA | NA | NA | NA | 1 |
| 1 | rs10798004 | C1orf25 C1orf26 IVNS1ABP RNF2 | 183531772 | A | G | 77.0 | 131.0 | 0.59 | 14.02 | 1.81E-04 | T | C | 0.420 | 1.02 | 1 |
| 16 | rs16953659 | IRX3 | 53144933 | A | G | 19.3 | 2.0 | 9.59 | 14.01 | 1.82E-04 | NA | NA | NA | NA | 1 |
| 10 | rs1914533 | CPXM2 | 125671264 | G | T | 54.4 | 101.1 | 0.54 | 13.99 | 1.84E-04 | NA | NA | NA | NA | 1 |
| 17 | rs8064924 | DOC2B LOC100506388 RPH3AL C17orf97 FAM101B | 18901 | A | G | 62.8 | 27.3 | 2.30 | 13.98 | 1.84E-04 | NA | NA | NA | NA | 1 |
| 18 | rs7245021 | L3MBTL4 | 6082906 | C | T | 89.6 | 46.1 | 1.95 | 13.98 | 1.85E-04 | A | G | 0.429 | 1.03 | 1 |
| 22 | rs5747405 | MICAL3 | 16775877 | C | T | 63.8 | 113.6 | 0.56 | 13.95 | 1.87E-04 | NA | NA | NA | NA | 1 |
| 22 | rs9626737 | PPARA | 44949006 | A | G | 20.1 | 2.4 | 8.36 | 13.95 | 1.88E-04 | NA | NA | NA | NA | 1 |
| 6 | rs4716071 | ATXN1 | 16541202 | A | G | 70.3 | 122.1 | 0.58 | 13.93 | 1.90E-04 | NA | NA | NA | NA | 1 |
| 1 | rs786911 | GTF2B PKN2 CCBL2 | 89037836 | C | T | 119.0 | 68.0 | 1.75 | 13.91 | 1.92E-04 | T | C | 0.634 | 0.99 | 1 |
| 2 | rs4972810 | DLX1 DLX2 PDK1 MAP1D ITGA6 | 172926135 | A | G | 56.7 | 23.4 | 2.43 | 13.90 | 1.93E-04 | A | G | 0.648 | 1.02 | 1 |
| 10 | rs7914368 | KIAA0157 METTL10 FAM53B ZRANB1 CTBP2 | 126559983 | C | T | 40.0 | 81.0 | 0.49 | 13.89 | 1.94E-04 | A | G | 0.777 | 0.99 | 1 |
| 4 | rs1373649 | BMPR1B | 96111734 | A | G | 65.0 | 115.0 | 0.57 | 13.89 | 1.94E-04 | G | A | 0.159 | 1.04 | 1 |
| 25 | rs2759061 | TGIF2LX | 88795349 | T | G | 33.0 | 71.0 | 0.46 | 13.88 | 1.94E-04 | NA | NA | NA | NA | 1 |
| 25 | rs2574663 | TGIF2LX | 89391373 | G | A | 33.0 | 71.0 | 0.46 | 13.88 | 1.94E-04 | NA | NA | NA | NA | 1 |
| 25 | rs3126493 | PCDH11X PABPC5 | 90797166 | T | C | 33.0 | 71.0 | 0.46 | 13.88 | 1.94E-04 | NA | NA | NA | NA | 1 |
| 25 | rs34188138 | PCDH11X PABPC5 | 91008768 | G | T | 33.0 | 71.0 | 0.46 | 13.88 | 1.94E-04 | NA | NA | NA | NA | 1 |
| 25 | rs2578811 | PCDH11X PABPC5 | 91575114 | A | C | 33.0 | 71.0 | 0.46 | 13.88 | 1.94E-04 | NA | NA | NA | NA | 1 |
| 25 | rs35166421 | PCDH11X PABPC5 | 91744721 | C | T | 33.0 | 71.0 | 0.46 | 13.88 | 1.94E-04 | NA | NA | NA | NA | 1 |
| 25 | rs2262813 | NAP1L3 FAM133A | 91959978 | A | G | 33.0 | 71.0 | 0.46 | 13.88 | 1.94E-04 | NA | NA | NA | NA | 1 |
| 25 | rs35766825 | NAP1L3 FAM133A | 92006619 | G | A | 33.0 | 71.0 | 0.46 | 13.88 | 1.94E-04 | NA | NA | NA | NA | 1 |
| 3 | rs9876767 | CBLB | 106932483 | G | T | 105.1 | 57.6 | 1.82 | 13.87 | 1.95E-04 | NA | NA | NA | NA | 1 |
| 4 | rs9990951 | HPGD | 175699205 | A | C | 110.1 | 61.3 | 1.80 | 13.87 | 1.96E-04 | A | C | 0.312 | 0.97 | 1 |
| 1 | rs4657741 | TIPRL IQWD1 BRP44 GPR161 ADCY10 | 166415766 | A | G | 123.6 | 71.6 | 1.73 | 13.86 | 1.97E-04 | NA | NA | NA | NA | 1 |
| 10 | rs11250483 | ADARB2 | 1406962 | C | T | 56.5 | 23.2 | 2.43 | 13.84 | 1.99E-04 | T | C | 0.565 | 0.98 | 1 |
| 7 | rs1072107 | ZNF804B | 89033451 | A | G | 21.5 | 53.8 | 0.40 | 13.84 | 1.99E-04 | NA | NA | NA | NA | 1 |
| 3 | rs9283662 | HRASLS ATP13A5 C3orf59 | 194240885 | C | T | 23.9 | 57.5 | 0.42 | 13.84 | 1.99E-04 | NA | NA | NA | NA | 1 |
| 1 | rs10749738 | FOXD3 | 63565743 | A | G | 128.0 | 75.0 | 1.71 | 13.84 | 1.99E-04 | T | C | 0.266 | 1.03 | 1 |
| 5 | rs1073933 | COX7C | 86307374 | C | T | 75.0 | 128.0 | 0.59 | 13.84 | 1.99E-04 | A | G | 0.108 | 0.96 | 1 |
| 1 | rs12029576 | TGFB2 | 216626098 | A | C | 55.4 | 102.1 | 0.54 | 13.84 | 1.99E-04 | *G* | *T* | *0.015* | 1.08 | 1 |
| 25 | rs2574511 | TGIF2LX | 89321500 | A | C | 31.0 | 68.0 | 0.46 | 13.83 | 2.00E-04 | NA | NA | NA | NA | 1 |
| 20 | rs297676 | PRNP PRND ADRA1D SMOX PRNT | 4391634 | A | G | 52.9 | 98.7 | 0.54 | 13.82 | 2.01E-04 | NA | NA | NA | NA | 1 |
| 4 | rs11733737 | MAML3 | 141043992 | C | T | 64.2 | 113.8 | 0.56 | 13.82 | 2.01E-04 | T | C | 0.157 | 0.96 | 1 |
| 15 | rs17703807 | C15orf41 | 34553348 | C | T | 61.1 | 109.6 | 0.56 | 13.80 | 2.03E-04 | C | T | 0.733 | 1.01 | 1 |
| 14 | rs1278878 | ARHGAP5-AS1 ARHGAP5 NUBPL AKAP6 GPR33 | 31454377 | A | G | 46.8 | 90.2 | 0.52 | 13.80 | 2.03E-04 | NA | NA | NA | NA | 1 |
| 15 | rs3883013 | ADAMTSL3 FLJ40113 | 82889661 | C | T | 69.4 | 120.6 | 0.58 | 13.80 | 2.04E-04 | NA | NA | NA | NA | 1 |
| 8 | rs4734058 | ODF1 UBR5 KLF10 | 103593352 | C | T | 38.0 | 78.0 | 0.49 | 13.79 | 2.04E-04 | A | G | 0.922 | 1.00 | 1 |
| 1 | rs13376289 | DHRS3 | 12583766 | G | T | 61.5 | 26.6 | 2.31 | 13.79 | 2.04E-04 | NA | NA | NA | NA | 1 |
| 13 | rs2810135 | DACH1 | 71395777 | A | G | 57.6 | 105.0 | 0.55 | 13.79 | 2.05E-04 | NA | NA | NA | NA | 1 |
| 1 | rs12746568 | FMO9P FAM78B | 164623408 | C | T | 43.8 | 86.1 | 0.51 | 13.78 | 2.06E-04 | C | T | 0.061 | 1.07 | 1 |
| 7 | rs11760288 | LMBR1 NOM1 MNX1 | 156394679 | A | G | 51.0 | 96.0 | 0.53 | 13.78 | 2.06E-04 | *T* | *C* | *0.039* | 0.93 | 1 |
| 1 | rs6672549 | NTNG1 | 107676482 | A | G | 34.0 | 9.6 | 3.56 | 13.76 | 2.07E-04 | G | A | 0.903 | 1.01 | 1 |
| 18 | rs17106 | PIEZO2 | 11064577 | C | T | 78.0 | 38.0 | 2.05 | 13.76 | 2.08E-04 | NA | NA | NA | NA | 1 |
| 3 | rs9837804 | FAM43A C3orf21 TMEM44 LSG1 | 196020410 | C | T | 60.3 | 25.9 | 2.33 | 13.76 | 2.08E-04 | A | G | 0.833 | 0.99 | 1 |
| 14 | rs1950538 | FSCB C14orf28 | 43600472 | C | T | 40.8 | 81.9 | 0.50 | 13.75 | 2.09E-04 | C | T | 0.148 | 0.95 | 1 |
| 15 | rs7167893 | OR4N4 OR4M2 | 19929330 | A | G | 4.9 | 25.3 | 0.19 | 13.75 | 2.09E-04 | NA | NA | NA | NA | 1 |
| 3 | rs777488 | CHST13 UROC1 | 127713078 | C | T | 67.7 | 118.2 | 0.57 | 13.75 | 2.09E-04 | NA | NA | NA | NA | 1 |
| 12 | rs624304 | EFCAB4B PARP11 | 3941061 | C | T | 30.3 | 66.8 | 0.45 | 13.75 | 2.09E-04 | NA | NA | NA | NA | 1 |
| 16 | rs935933 | PKD1L2 | 79766735 | C | T | 44.6 | 15.8 | 2.82 | 13.74 | 2.10E-04 | NA | NA | NA | NA | 1 |
| 19 | rs7255066 | PVR | 49837943 | C | T | 100.0 | 54.0 | 1.85 | 13.74 | 2.10E-04 | C | T | 0.197 | 1.04 | 1 |
| 6 | rs9467027 | DCDC2 NRSN1 | 24167386 | C | T | 66.0 | 116.0 | 0.57 | 13.74 | 2.10E-04 | NA | NA | NA | NA | 1 |
| 1 | rs7537000 | VAV3 | 108177825 | A | G | 62.3 | 111.1 | 0.56 | 13.73 | 2.11E-04 | C | T | 0.415 | 0.98 | 1 |
| 22 | rs8190315 | BID BCL2L13 SLC25A18 ATP6V1E1 | 16606764 | C | T | 0.0 | 13.8 | 0.00 | 13.72 | 2.12E-04 | NA | NA | NA | NA | 1 |
| 1 | rs12023326 | VN1R5 ZNF496 LOC441931 | 245537744 | C | T | 46.1 | 89.2 | 0.52 | 13.71 | 2.13E-04 | G | A | 0.056 | 0.94 | 1 |
| 1 | rs6426239 | VN1R5 ZNF496 LOC441931 | 245553359 | C | T | 89.2 | 46.1 | 1.93 | 13.71 | 2.13E-04 | A | G | 0.056 | 0.94 | 1 |
| 1 | rs6690864 | VN1R5 ZNF496 LOC441931 | 245557330 | A | G | 46.0 | 89.0 | 0.52 | 13.70 | 2.15E-04 | A | G | 0.072 | 0.94 | 1 |
| 1 | rs10218647 | FCER1A OR10J3 OR10J5 OR10J1 DARC | 157586427 | A | G | 69.7 | 120.8 | 0.58 | 13.70 | 2.15E-04 | NA | NA | NA | NA | 1 |
| 1 | rs11811613 | DUSP10 | 220122026 | A | G | 49.4 | 93.6 | 0.53 | 13.69 | 2.16E-04 | G | A | 0.315 | 0.97 | 1 |
| 3 | rs4283545 | APPL1 HESX1 IL17RD ASB14 | 57175798 | A | C | 138.0 | 83.0 | 1.66 | 13.69 | 2.16E-04 | C | A | 0.678 | 0.99 | 1 |
| 2 | rs4972809 | DLX1 DLX2 PDK1 MAP1D ITGA6 | 172925337 | A | G | 56.9 | 23.7 | 2.40 | 13.69 | 2.16E-04 | NA | NA | NA | NA | 1 |
| 10 | rs4752123 | EMX2OS RAB11FIP2 EMX2 | 119603623 | C | T | 83.3 | 41.9 | 1.99 | 13.68 | 2.17E-04 | NA | NA | NA | NA | 1 |
| 3 | rs7639059 | MITF | 70244281 | A | C | 125.2 | 73.1 | 1.71 | 13.67 | 2.18E-04 | C | A | 0.844 | 1.00 | 1 |
| 1 | rs6697017 | EBNA1BP2 | 43524288 | C | T | 47.6 | 17.7 | 2.69 | 13.67 | 2.18E-04 | NA | NA | NA | NA | 1 |
| 6 | rs9294965 | THBS2 | 169301431 | A | G | 14.2 | 41.9 | 0.34 | 13.66 | 2.20E-04 | NA | NA | NA | NA | 1 |
| 10 | rs7093944 | PITRM1 PFKP | 3220052 | A | C | 37.4 | 76.9 | 0.49 | 13.65 | 2.21E-04 | NA | NA | NA | NA | 1 |
| 14 | rs12147287 | MIR656 MIR410 MIR369 MIR412 MIR409 | 100624592 | A | G | 85.1 | 43.2 | 1.97 | 13.64 | 2.21E-04 | NA | NA | NA | NA | 1 |
| 6 | rs4710911 | ID4 MBOAT1 | 19657847 | A | C | 93.0 | 49.0 | 1.90 | 13.63 | 2.22E-04 | T | G | 0.506 | 1.02 | 1 |
| 1 | rs10157571 | MIR205HG MIR205 | 207159096 | C | T | 119.0 | 68.5 | 1.74 | 13.62 | 2.24E-04 | A | G | 0.454 | 0.98 | 1 |
| 20 | rs17803622 | SALL4 ATP9A ZFP64 | 50081298 | C | T | 33.4 | 9.3 | 3.58 | 13.59 | 2.28E-04 | NA | NA | NA | NA | 1 |
| 10 | rs10786998 | SORCS1 | 108700117 | A | C | 130.0 | 77.0 | 1.69 | 13.57 | 2.30E-04 | A | C | 0.205 | 0.96 | 1 |
| 10 | rs4752324 | RGS10 | 121280561 | C | T | 44.0 | 86.0 | 0.51 | 13.57 | 2.30E-04 | T | C | 0.928 | 1.00 | 1 |
| 3 | rs1867189 | CBLB | 106930436 | A | G | 106.6 | 59.2 | 1.80 | 13.57 | 2.30E-04 | T | C | 0.242 | 0.97 | 1 |
| 20 | rs13037722 | TGM3 | 2263305 | C | T | 23.4 | 56.3 | 0.42 | 13.56 | 2.31E-04 | C | T | 0.881 | 0.99 | 1 |
| 12 | rs11059101 | FLJ37505 LOC440117 | 126440203 | C | T | 38.2 | 77.9 | 0.49 | 13.56 | 2.31E-04 | NA | NA | NA | NA | 1 |
| 6 | rs13195509 | HISTONE-cluster ZNF322A BTN1A1 BTN3A3 BTN2A2 SCGN SLC17A2 HMGN4 TRIM38 BTN2A1 HFE SLC17A1 SLC17A3 SLC17A4 BTN2A3 PRSS16 BTN3A1,A2 | 26571639 | A | G | 79.0 | 39.0 | 2.03 | 13.56 | 2.31E-04 | NA | NA | NA | NA | 1 |
| 25 | rs2525146 | PCDH11X PABPC5 | 91051186 | A | G | 32.0 | 69.0 | 0.46 | 13.55 | 2.32E-04 | NA | NA | NA | NA | 1 |
| 2 | rs7587759 | SATB2 FLJ32063 | 199365265 | C | T | 78.0 | 38.3 | 2.04 | 13.55 | 2.32E-04 | *A* | *G* | *0.021* | 1.09 | 1 |
| 1 | rs10482751 | TGFB2 | 216622920 | C | T | 61.0 | 109.0 | 0.56 | 13.55 | 2.32E-04 | T | C | 0.059 | 1.06 | 1 |
| 15 | rs9806182 | DYX1C1-CCPG1 PYGO1 | 53610894 | A | G | 109.0 | 61.0 | 1.79 | 13.55 | 2.32E-04 | C | T | 0.565 | 0.98 | 1 |
| 11 | rs12360673 | GRIK4 | 120049447 | A | G | 61.5 | 26.9 | 2.29 | 13.54 | 2.33E-04 | G | A | 0.619 | 0.98 | 1 |
| 6 | rs6938557 | RIMS1 OGFRL1 | 72413501 | A | G | 134.0 | 80.1 | 1.67 | 13.52 | 2.35E-04 | A | G | 0.678 | 1.01 | 1 |
| 12 | rs11836636 | LOC552889 KCNC2 | 73349377 | A | G | 86.0 | 44.1 | 1.95 | 13.52 | 2.36E-04 | G | A | 0.450 | 1.03 | 1 |
| 11 | rs10501156 | PRR5L | 36427391 | A | C | 12.0 | 38.0 | 0.32 | 13.52 | 2.36E-04 | T | G | 0.462 | 0.97 | 1 |
| 10 | rs11013804 | KIAA1217 | 24270246 | C | T | 48.5 | 92.0 | 0.53 | 13.51 | 2.38E-04 | NA | NA | NA | NA | 1 |
| 1 | rs10801687 | GTF2B PKN2 CCBL2 | 89079849 | A | G | 68.7 | 119.1 | 0.58 | 13.50 | 2.38E-04 | T | C | 0.685 | 0.99 | 1 |
| 10 | rs10829685 | EBF3 GLRX3 | 131746641 | G | T | 66.0 | 30.0 | 2.20 | 13.50 | 2.39E-04 | T | G | 0.742 | 1.01 | 1 |
| 1 | rs10919271 | KIFAP3 SELL SELE C1orf156 C1orf112 SCYL3 | 168171677 | A | G | 73.7 | 35.3 | 2.09 | 13.49 | 2.39E-04 | NA | NA | NA | NA | 1 |
| 5 | rs1866543 | ZNF608 GRAMD3 | 124518715 | A | G | 76.1 | 37.0 | 2.06 | 13.49 | 2.39E-04 | NA | NA | NA | NA | 1 |
| 10 | rs10509825 | SORCS1 | 108675202 | C | T | 81.1 | 135.0 | 0.60 | 13.49 | 2.40E-04 | A | G | 0.883 | 1.00 | 1 |
| 2 | rs7608732 | LOC100506274 LOC386597 | 7500762 | A | C | 23.0 | 55.6 | 0.41 | 13.49 | 2.40E-04 | A | C | 0.840 | 0.99 | 1 |
| 6 | rs744143 | E2F3 | 20526697 | A | G | 16.3 | 45.0 | 0.36 | 13.45 | 2.45E-04 | NA | NA | NA | NA | 1 |
| 13 | rs9599966 | RP11-11C5.2 DIS3 C13orf34 PIBF1 | 71987893 | A | G | 74.0 | 35.6 | 2.08 | 13.45 | 2.45E-04 | NA | NA | NA | NA | 1 |
| 11 | rs10836470 | OR51E2 | 4664147 | C | T | 42.0 | 83.0 | 0.51 | 13.45 | 2.45E-04 | A | G | 0.617 | 0.98 | 1 |
| 8 | rs1467073 | DENND3 SLC45A4 PTK2 LOC731779 GPR20 | 142202147 | A | G | 14.6 | 42.3 | 0.35 | 13.45 | 2.45E-04 | NA | NA | NA | NA | 1 |
| 8 | rs12680914 | CHD7 | 61990230 | G | T | 60.2 | 26.2 | 2.30 | 13.44 | 2.46E-04 | NA | NA | NA | NA | 1 |
| 4 | rs1373651 | BMPR1B | 96087548 | A | G | 68.0 | 118.0 | 0.58 | 13.44 | 2.46E-04 | G | A | 0.202 | 1.03 | 1 |
| 2 | rs1900710 | BCL2L11 ACOXL | 111929715 | C | T | 10.1 | 34.5 | 0.29 | 13.41 | 2.50E-04 | G | A | 0.813 | 1.01 | 1 |
| 2 | rs9798096 | ADI1 RNASEH1 RPS7 COLEC11 | 3488001 | C | T | 76.3 | 37.3 | 2.05 | 13.41 | 2.50E-04 | NA | NA | NA | NA | 1 |
| 4 | rs17005153 | RASGEF1B | 82651548 | A | G | 114.1 | 65.1 | 1.75 | 13.41 | 2.51E-04 | NA | NA | NA | NA | 1 |
| 14 | rs4457900 | MNAT1 TMEM30B PRKCH SLC38A6 TRMT5 | 60759645 | G | T | 106.0 | 59.0 | 1.80 | 13.40 | 2.52E-04 | *T* | *G* | *0.013* | 0.93 | 1 |
| 3 | rs12491867 | SOX2OT | 182931252 | A | G | 23.1 | 55.6 | 0.42 | 13.40 | 2.52E-04 | A | G | 0.076 | 0.91 | 1 |
| 1 | rs10927905 | PDPN | 13789584 | C | T | 49.7 | 19.3 | 2.57 | 13.39 | 2.53E-04 | C | T | 0.574 | 0.97 | 1 |
| 13 | rs9542781 | DACH1 | 71406895 | A | G | 59.5 | 106.6 | 0.56 | 13.38 | 2.54E-04 | NA | NA | NA | NA | 1 |
| 7 | rs2192268 | PMS2L2 | 76282584 | G | T | 24.5 | 57.6 | 0.42 | 13.38 | 2.54E-04 | NA | NA | NA | NA | 1 |
| 3 | rs2305037 | CBLB | 106905534 | C | T | 104.1 | 57.6 | 1.81 | 13.38 | 2.54E-04 | NA | NA | NA | NA | 1 |
| 17 | rs1107704 | CD300C CD300LB | 70020020 | A | G | 67.0 | 30.8 | 2.17 | 13.38 | 2.54E-04 | G | A | 0.835 | 0.99 | 1 |
| 4 | rs624909 | CXXC4 | 105785890 | G | T | 82.0 | 136.0 | 0.60 | 13.38 | 2.55E-04 | *T* | *G* | *0.008* | 0.93 | 1 |
| 22 | rs17304019 | PDGFB RPL3 SYNGR1 MAP3K7IP1 SNORD43 | 37991543 | A | G | 10.3 | 34.9 | 0.30 | 13.37 | 2.55E-04 | *A* | *G* | *0.025* | 0.86 | 1 |
| 6 | rs7759896 | GUSBL2 KHDRBS2 PRIM2 | 57699799 | C | T | 118.0 | 68.1 | 1.73 | 13.37 | 2.55E-04 | NA | NA | NA | NA | 1 |
| 10 | rs911607 | VIM | 17342897 | A | G | 102.1 | 56.1 | 1.82 | 13.37 | 2.56E-04 | NA | NA | NA | NA | 1 |
| 22 | rs5992834 | BID | 16641496 | A | G | 6.8 | 28.5 | 0.24 | 13.37 | 2.56E-04 | NA | NA | NA | NA | 1 |
| 3 | rs1479146 | STAC | 36336003 | A | G | 67.9 | 117.7 | 0.58 | 13.35 | 2.58E-04 | NA | NA | NA | NA | 1 |
| 10 | rs4420167 | KIAA1217 PRINS | 24273620 | A | G | 61.3 | 108.9 | 0.56 | 13.34 | 2.60E-04 | NA | NA | NA | NA | 1 |
| 1 | rs715643 | MRPL20 B3GALT6 UBE2J2 CALML6 CCNL2 SDF4 TNFRSF18 GLTPD1 SLC35E2 C1orf159 ATAD3B CENTB5 ATAD3C TAS1R3 ATAD3A MXRA8 SCNN1D SSU72 TMEM52 RP11-345P4.4 MIB2 PUSL1 CPSF3L TTLL10 NADK MMP23B MMP23A FAM132A DVL1 AURKAIP1 CDC2L1 VWA1 GNB1 TNFRSF4 | 1162770 | C | T | 43.9 | 15.7 | 2.79 | 13.33 | 2.61E-04 | A | G | 0.751 | 1.02 | 1 |
| 15 | rs11635863 | LOC400456 LOC145820 | 93410125 | A | G | 25.0 | 5.0 | 5.00 | 13.33 | 2.61E-04 | G | A | 0.498 | 1.05 | 1 |
| 2 | rs7604827 | SPAG16 | 215050135 | C | T | 26.1 | 60.0 | 0.44 | 13.32 | 2.62E-04 | G | A | 0.639 | 1.02 | 1 |
| 19 | rs11671562 | ZNF507 | 37671687 | C | T | 98.9 | 53.8 | 1.84 | 13.31 | 2.64E-04 | NA | NA | NA | NA | 1 |
| 2 | rs17010928 | GALNT14 | 31268558 | A | G | 9.2 | 32.9 | 0.28 | 13.31 | 2.65E-04 | A | G | 0.873 | 0.99 | 1 |
| 25 | rs2766234 | CPXCR1 TGIF2LX | 88405398 | T | G | 33.0 | 70.0 | 0.47 | 13.29 | 2.67E-04 | NA | NA | NA | NA | 1 |
| 25 | rs2534116 | TGIF2LX | 88571714 | T | C | 33.0 | 70.0 | 0.47 | 13.29 | 2.67E-04 | NA | NA | NA | NA | 1 |
| 25 | rs35948456 | TGIF2LX CPXCR1 | 89070679 | A | G | 33.0 | 70.0 | 0.47 | 13.29 | 2.67E-04 | NA | NA | NA | NA | 1 |
| 1 | rs10489177 | KIFAP3 SELL SELE C1orf156 C1orf112 SCYL3 | 168029431 | G | T | 70.0 | 33.0 | 2.12 | 13.29 | 2.67E-04 | G | T | 0.875 | 1.01 | 1 |
| 18 | rs4467169 | SETBP1 | 40406465 | A | G | 33.0 | 70.0 | 0.47 | 13.29 | 2.67E-04 | G | A | 0.885 | 1.01 | 1 |
| 14 | rs1676235 | ESRRB ANGEL1 VASH1 | 76088319 | A | G | 53.3 | 98.2 | 0.54 | 13.28 | 2.68E-04 | T | C | 0.148 | 0.96 | 1 |
| 6 | rs9379089 | SSR1 CAGE1 | 7275168 | C | T | 7.1 | 29.1 | 0.25 | 13.27 | 2.69E-04 | NA | NA | NA | NA | 1 |
| 3 | rs9880861 | CBLB | 106932676 | C | T | 104.6 | 58.2 | 1.80 | 13.26 | 2.71E-04 | C | T | 0.358 | 0.97 | 1 |
| 10 | rs7079264 | SORCS1 | 108669882 | A | G | 76.0 | 128.0 | 0.59 | 13.25 | 2.72E-04 | G | A | 0.589 | 0.98 | 1 |
| 13 | rs7318477 | ABHD13 LIG4 MYO16 TNFSF13B | 107835962 | C | T | 99.0 | 54.0 | 1.83 | 13.24 | 2.75E-04 | A | G | 0.450 | 1.03 | 1 |
| 10 | rs7072699 | ADARB2 | 1682796 | C | T | 103.0 | 57.0 | 1.81 | 13.23 | 2.76E-04 | T | C | 0.779 | 0.99 | 1 |
| 1 | rs3856145 | LBR ENAH DNAH14 SRP9 EPHX1 | 223594806 | A | C | 67.0 | 31.0 | 2.16 | 13.22 | 2.76E-04 | NA | NA | NA | NA | 1 |
| 12 | rs11180157 | LOC552889 KCNC2 | 73363611 | C | T | 44.1 | 85.4 | 0.52 | 13.22 | 2.77E-04 | C | T | 0.496 | 1.03 | 1 |
| 8 | rs11166827 | FAM135B COL22A1 | 139573452 | C | T | 58.4 | 104.9 | 0.56 | 13.22 | 2.77E-04 | G | A | 0.415 | 0.98 | 1 |
| 9 | rs12553751 | PRUNE2 | 78703074 | A | G | 13.5 | 40.1 | 0.34 | 13.22 | 2.77E-04 | T | C | 0.417 | 1.06 | 1 |
| 18 | rs4426448 | DOK6 | 65516648 | A | G | 81.2 | 134.6 | 0.60 | 13.19 | 2.81E-04 | NA | NA | NA | NA | 1 |
| 4 | rs13122067 | GALNT17 | 173140885 | C | T | 35.2 | 10.6 | 3.31 | 13.19 | 2.81E-04 | A | G | 0.199 | 1.07 | 1 |
| 23 | rs11092298 | TAF7L DRP2 TIMM8A BTK RPL36A-HNRNPH2GLA | 100412624 | C | T | 64.0 | 29.0 | 2.21 | 13.17 | 2.84E-04 | C | T | 0.138 | 1.04 | 1 |
| 23 | rs3788765 | TAF7L DRP2 TIMM8A BTK RPL36A-HNRNPH2 | 100419580 | G | A | 64.0 | 29.0 | 2.21 | 13.17 | 2.84E-04 | C | T | 0.151 | 1.04 | 1 |
| 8 | rs2956060 | LY96 JPH1 GDAP1 TMEM70 TCEB1 | 75272339 | A | G | 97.6 | 53.1 | 1.84 | 13.17 | 2.84E-04 | NA | NA | NA | NA | 1 |
| 5 | rs11953285 | ADRA1B | 159324389 | A | C | 66.4 | 30.6 | 2.17 | 13.17 | 2.84E-04 | C | A | 0.607 | 1.02 | 1 |
| 6 | rs10484439 | HISTONE-cluster ZNF322A BTN1A1 BTN3A3 BTN2A2 SCGN SLC17A2 HMGN4 TRIM38 BTN2A1 HFE SLC17A1 SLC17A3 SLC17A4 BTN2A3 PRSS16 BTN3A1,A2 | 26417887 | A | G | 72.8 | 35.1 | 2.07 | 13.17 | 2.85E-04 | NA | NA | NA | NA | 1 |
| 22 | rs2187956 | SRRD SRRD HPS4 TFIP11 ASPHD2 | 25224985 | C | T | 55.9 | 23.6 | 2.37 | 13.16 | 2.87E-04 | NA | NA | NA | NA | 1 |
| 22 | rs1008530 | SRRD SRRD HPS4 TFIP11 ASPHD2 | 25225337 | A | G | 23.6 | 55.9 | 0.42 | 13.16 | 2.87E-04 | NA | NA | NA | NA | 1 |
| 1 | rs786921 | GTF2B PKN2 CCBL2 | 89059261 | A | G | 133.1 | 80.1 | 1.66 | 13.15 | 2.87E-04 | NA | NA | NA | NA | 1 |
| 15 | rs10518687 | MYO5A | 50474725 | A | G | 108.8 | 61.5 | 1.77 | 13.14 | 2.89E-04 | *C* | *T* | *0.006* | 1.08 | 1 |
| 3 | rs2268844 | DGKG | 187403297 | G | T | 51.5 | 20.7 | 2.49 | 13.13 | 2.90E-04 | T | G | 0.858 | 0.99 | 1 |
| 15 | rs8024188 | ATP10A | 23887556 | A | G | 79.9 | 40.2 | 1.99 | 13.13 | 2.91E-04 | NA | NA | NA | NA | 1 |
| 12 | rs1366041 | NUAK1 | 105008642 | C | T | 20.4 | 51.1 | 0.40 | 13.13 | 2.91E-04 | G | A | 0.798 | 0.99 | 1 |
| 6 | rs7763790 | TTRAP KIAA0319 | 24723042 | A | G | 74.0 | 36.0 | 2.06 | 13.13 | 2.91E-04 | NA | NA | NA | NA | 1 |
| 6 | rs6923139 | HISTONE-cluster ZNF322A BTN1A1 BTN3A3 BTN2A2 SCGN SLC17A2 HMGN4 TRIM38 BTN2A1 HFE SLC17A1 SLC17A3 SLC17A4 BTN2A3 PRSS16 BTN3A1,A2 | 26421327 | C | T | 36.0 | 74.0 | 0.49 | 13.13 | 2.91E-04 | NA | NA | NA | NA | 1 |
| 11 | rs1520895 | NAV2 HTATIP2 DBX1 PRMT3 | 20232522 | A | G | 129.0 | 77.0 | 1.68 | 13.13 | 2.91E-04 | A | G | 0.706 | 0.99 | 1 |
| 15 | rs2242058 | MYO5A | 50459076 | C | T | 61.5 | 108.8 | 0.57 | 13.13 | 2.91E-04 | *C* | *T* | *0.009* | 1.07 | 1 |
| 22 | rs4820682 | SRRD HPS4 | 25214039 | A | G | 25.0 | 58.0 | 0.43 | 13.12 | 2.92E-04 | *G* | *A* | *0.043* | 0.92 | 1 |
| 4 | rs7661496 | KIAA1712 FBXO8 | 175334212 | A | G | 16.4 | 44.8 | 0.37 | 13.11 | 2.93E-04 | NA | NA | NA | NA | 1 |
| 14 | rs2370876 | NRXN3 | 78415889 | A | G | 31.3 | 8.5 | 3.70 | 13.11 | 2.94E-04 | G | A | 0.747 | 1.02 | 1 |
| 9 | rs7871815 | LRRC19 C9orf82 IFT74 PLAA | 26684184 | A | G | 92.1 | 49.1 | 1.88 | 13.10 | 2.95E-04 | NA | NA | NA | NA | 1 |
| 23 | rs5991739 | MAGED1 GSPT2 CENPVL1 LOC441495 SNORA11E | 51627709 | A | G | 34.0 | 10.0 | 3.40 | 13.09 | 2.97E-04 | NA | NA | NA | NA | 1 |
| 19 | rs846866 | PVR CEACAM19 | 49825950 | A | C | 69.0 | 32.6 | 2.12 | 13.09 | 2.97E-04 | NA | NA | NA | NA | 1 |
| 20 | rs6065961 | CDH22 SLC35C2 ZNF334 ELMO2 SLC13A3 NCOA5 CD40 | 44401662 | A | C | 25.0 | 57.9 | 0.43 | 13.09 | 2.98E-04 | T | G | 0.400 | 0.97 | 1 |
| 1 | rs4657739 | TIPRL IQWD1 BRP44 GPR161 ADCY10 | 166409113 | C | T | 52.0 | 96.0 | 0.54 | 13.08 | 2.98E-04 | A | G | 0.363 | 1.03 | 1 |
| 13 | rs1563871 | DACH1 | 71401505 | C | T | 102.1 | 56.5 | 1.81 | 13.08 | 2.99E-04 | T | C | 0.710 | 0.99 | 1 |
| 1 | rs2208577 | MIER1 WDR78 SLC35D1 | 67286162 | A | C | 62.0 | 109.4 | 0.57 | 13.07 | 3.00E-04 | *A* | *C* | *0.011* | 0.93 | 1 |
| 11 | rs1145212 | APOA5 ZNF259 BUD13 | 116062075 | A | G | 61.0 | 108.0 | 0.56 | 13.07 | 3.00E-04 | A | G | 0.869 | 1.00 | 1 |
| 8 | rs12334475 | TSNARE1 | 143143454 | C | T | 14.5 | 0.6 | 25.88 | 12.90 | 3.29E-04 | NA | NA | NA | NA | 2 |
| 4 | rs6553634 | GALNT17 | 173654879 | A | G | 37.0 | 75.0 | 0.49 | 12.89 | 3.30E-04 | *C* | *T* | *0.042* | 1.08 | 3 |
| 23 | rs5918890 | ZC4H2 MTMR8 | 64004113 | A | G | 18.0 | 2.0 | 9.00 | 12.80 | 3.47E-04 | T | C | 0.690 | 0.98 | 2 |
| 23 | rs7891628 | IL13RA2 RBMXL3 MIR448 LRCH2 LUZP4 | 114169292 | C | A | 2.0 | 18.0 | 0.11 | 12.80 | 3.47E-04 | C | A | 0.180 | 0.95 | 2 |
| 1 | rs4655662 | MIER1 WDR78 SLC35D1 | 67189020 | C | T | 123.1 | 73.1 | 1.68 | 12.75 | 3.57E-04 | *A* | *G* | *0.001* | 0.91 | 3 |
| 1 | rs2755242 | MIER1 WDR78 SLC35D1 | 67252186 | C | T | 123.1 | 73.1 | 1.68 | 12.74 | 3.58E-04 | *A* | *G* | *0.001* | 0.91 | 3 |
| 1 | rs2065002 | MIER1 WDR78 SLC35D1 | 67259707 | A | C | 123.1 | 73.1 | 1.68 | 12.74 | 3.58E-04 | *C* | *A* | *0.001* | 0.91 | 3 |
| 13 | rs9301029 | DAOA | 104896251 | C | T | 103.2 | 58.1 | 1.77 | 12.57 | 3.92E-04 | *T* | *C* | *0.042* | 0.94 | 3 |
| 23 | rs5988087 | MIR1298 MIR1264 MIR1912 MIR764 MIR1911 | 113841112 | A | G | 3.0 | 20.0 | 0.15 | 12.57 | 3.93E-04 | A | G | 0.982 | 1.00 | 2 |
| 23 | rs4332303 | MIR448 MIR1911 MIR1298 MIR1264 MIR1912 | 113954123 | A | G | 3.0 | 20.0 | 0.15 | 12.57 | 3.93E-04 | T | C | 0.823 | 0.99 | 2 |
| 5 | rs17207814 | SIL1 | 138316429 | A | G | 1.0 | 15.2 | 0.07 | 12.43 | 4.21E-04 | NA | NA | NA | NA | 2 |
| 1 | rs1930278 | TTLL7 PRKACB | 83258666 | A | G | 4.3 | 22.5 | 0.19 | 12.32 | 4.47E-04 | NA | NA | NA | NA | 2 |
| 3 | rs13096142 | CCR5 CCR3 LTF CCR2 CCR1 | 46256748 | C | T | 63.0 | 109.0 | 0.58 | 12.30 | 4.52E-04 | *T* | *C* | *0.000* | 1.16 | 3 |
| 12 | rs17005224 | SYT1 PAWR | 78030993 | A | G | 1.0 | 15.0 | 0.07 | 12.25 | 4.65E-04 | NA | NA | NA | NA | 2 |
| 12 | rs2730666 | TBC1D15 TPH2 TRHDE | 70856013 | G | T | 22.2 | 4.2 | 5.24 | 12.20 | 4.79E-04 | A | C | 0.162 | 0.91 | 2 |
| 14 | rs1841722 | C14orf64 LOC100129345 | 97392233 | G | T | 44.8 | 84.5 | 0.53 | 12.17 | 4.85E-04 | *C* | *A* | *0.049* | 0.94 | 3 |
| 1 | rs11585690 | AFARP1 LRIG2 SLC16A1 | 113257891 | A | C | 21.8 | 4.0 | 5.38 | 12.17 | 4.87E-04 | NA | NA | NA | NA | 2 |
| 2 | rs231757 | ICOS CTLA4 | 204461732 | G | T | 99.9 | 56.5 | 1.77 | 12.09 | 5.07E-04 | *C* | *A* | *0.002* | 1.12 | 3 |
| 21 | rs990628 | LINC00478,C21orf34 | 16754742 | A | G | 14.6 | 0.9 | 16.19 | 12.08 | 5.10E-04 | NA | NA | NA | NA | 2 |
| 11 | rs12793517 | LINC00167 PRDM10 APLP2 NFRKB ST14 | 129370802 | A | G | 0.0 | 12.0 | 0.00 | 12.00 | 5.32E-04 | NA | NA | NA | NA | 2 |
| 3 | rs6766451 | LRRC33 | 197859537 | G | T | 44.0 | 83.0 | 0.53 | 11.98 | 5.39E-04 | *G* | *T* | *0.014* | 0.93 | 3 |
| 2 | rs801298 | SLC39A10 DNAH7 | 195289944 | C | T | 21.5 | 4.0 | 5.34 | 11.94 | 5.49E-04 | T | C | 0.238 | 0.90 | 2 |
| 1 | rs11579106 | FAM36A HNRNPU | 243074738 | C | T | 19.2 | 3.0 | 6.51 | 11.93 | 5.52E-04 | NA | NA | NA | NA | 2 |
| 8 | rs12544542 | TRPS1 | 116885616 | A | G | 18.0 | 2.4 | 7.47 | 11.90 | 5.60E-04 | A | G | 0.771 | 0.97 | 2 |
| 2 | rs11890284 | ICOS CTLA4 | 204485649 | C | T | 59.1 | 103.0 | 0.57 | 11.89 | 5.66E-04 | *T* | *C* | *0.000* | 1.13 | 3 |
| 19 | rs10403336 | PRAM1 ZNF414 MYO1F | 8472880 | C | T | 17.3 | 2.1 | 8.18 | 11.84 | 5.79E-04 | NA | NA | NA | NA | 2 |
| 1 | rs11120695 | USH2A | 214146753 | G | T | 87.2 | 138.8 | 0.63 | 11.80 | 5.91E-04 | *T* | *G* | *0.034* | 1.06 | 3 |
| 12 | rs1803343 | LUM DCN | 90063915 | C | T | 2.2 | 17.3 | 0.13 | 11.74 | 6.11E-04 | NA | NA | NA | NA | 2 |
| 3 | rs11711054 | CCR5 CCR3 LTF CCR2 CCR1 | 46320615 | A | G | 59.2 | 102.6 | 0.58 | 11.65 | 6.41E-04 | *G* | *A* | *0.000* | 1.16 | 3 |
| 23 | rs11167436 | MIR1298 MIR1911 MIR1264 MIR1912 MIR764 | 113850316 | A | C | 3.0 | 19.0 | 0.16 | 11.64 | 6.47E-04 | T | G | 0.951 | 1.00 | 2 |
| 16 | rs8045954 | FTO IRX3 | 52999984 | A | C | 14.2 | 1.0 | 14.96 | 11.61 | 6.55E-04 | NA | NA | NA | NA | 2 |
| 9 | rs4842007 | PAEP | 137614606 | C | T | 72.0 | 119.0 | 0.61 | 11.57 | 6.72E-04 | *A* | *G* | *0.036* | 1.07 | 3 |
| 17 | rs7225029 | RNF213 ENDOV LOC100294362 MIR4730 SGSH | 75912374 | A | G | 4.0 | 21.0 | 0.19 | 11.56 | 6.74E-04 | NA | NA | NA | NA | 2 |
| 20 | rs6076132 | CST2 | 23749747 | A | C | 4.0 | 21.0 | 0.19 | 11.56 | 6.74E-04 | G | T | 0.853 | 1.02 | 2 |
| 23 | rs543229 | HTR2C SNORA35 MIR764 MIR1912 MIR1264 | 113727242 | C | T | 4.0 | 21.0 | 0.19 | 11.56 | 6.74E-04 | C | T | 0.516 | 0.98 | 2 |
| 6 | rs9397928 | ARID1B MIR4466 | 156714935 | C | T | 56.0 | 98.0 | 0.57 | 11.45 | 7.13E-04 | *G* | *A* | *0.039* | 0.94 | 3 |
| 5 | rs682828 | OR2Y1 | 180147080 | C | T | 74.7 | 38.9 | 1.92 | 11.26 | 7.94E-04 | *T* | *C* | *0.002* | 0.90 | 3 |
| 3 | rs9851967 | LPP | 189570322 | C | T | 117.0 | 71.0 | 1.65 | 11.26 | 7.94E-04 | *T* | *C* | *0.000* | 0.77 | 3 |
| 14 | rs987629 | CTSG C14orf124 GZMB GZMH KIAA0323 CMA1 | 24075425 | C | T | 118.2 | 72.1 | 1.64 | 11.13 | 8.48E-04 | *G* | *A* | *0.046* | 1.05 | 3 |
| 23 | rs5983336 | FAM133A NAP1L3 | 93209681 | C | A | 8.0 | 28.0 | 0.29 | 11.11 | 8.58E-04 | *G* | *T* | *0.048* | 0.94 | 3 |
| 3 | rs6441961 | CCR5 CCR3 LTF CCR2 CCR1 | 46327388 | C | T | 59.0 | 101.0 | 0.58 | 11.03 | 8.99E-04 | *T* | *C* | *0.000* | 1.17 | 3 |
| 23 | rs6643623 | ATP2B3 BGN FAM58A HAUS7 TREX2 | 152462936 | G | A | 0.0 | 11.0 | 0.00 | 11.00 | 9.11E-04 | G | A | 0.538 | 1.03 | 2 |
| 2 | rs10498225 | ARMC9 B3GNT7 | 231899229 | A | C | 1.6 | 15.1 | 0.10 | 10.96 | 9.29E-04 | NA | NA | NA | NA | 2 |
| 6 | rs12209247 | SERINC1 HSF2 PKIB | 123023249 | C | T | 47.7 | 20.4 | 2.34 | 10.92 | 9.49E-04 | *G* | *A* | *0.038* | 1.11 | 3 |
| 11 | rs2900740 | WT1 | 32392105 | A | G | 1.8 | 15.5 | 0.11 | 10.90 | 9.60E-04 | NA | NA | NA | NA | 2 |
| 12 | rs4964933 | MMP17 | 130905910 | C | T | 16.0 | 2.0 | 8.00 | 10.89 | 9.67E-04 | T | C | 0.151 | 0.90 | 2 |
| 4 | rs6830776 | LOC340017 GRIA2 | 158722200 | C | T | 16.6 | 2.3 | 7.36 | 10.89 | 9.68E-04 | NA | NA | NA | NA | 2 |
| 14 | rs6573374 | TMEM30B PRKCH | 60762708 | C | T | 67.0 | 111.0 | 0.60 | 10.88 | 9.74E-04 | *C* | *T* | *0.017* | 0.94 | 3 |
| 8 | rs4385459 | LY96 JPH1 GDAP1 TMEM70 TCEB1 | 75259545 | A | G | 82.0 | 130.0 | 0.63 | 10.87 | 9.78E-04 | *A* | *G* | *0.017* | 0.94 | 3 |
| 6 | rs551923 | EPM2A UTRN | 145585216 | A | G | 124.0 | 77.3 | 1.60 | 10.83 | 9.97E-04 | *A* | *G* | *0.047* | 1.06 | 3 |
| 9 | rs7046385 | SMC2 | 105644054 | C | T | 71.0 | 116.0 | 0.61 | 10.83 | 9.99E-04 | *C* | *T* | *0.025* | 0.94 | 3 |
| 12 | rs10778771 | LIN7A | 79842145 | A | C | 45.0 | 82.0 | 0.55 | 10.78 | 1.03E-03 | *C* | *A* | *0.026* | 1.08 | 3 |
| 5 | rs1353885 | TRPC7 | 135672914 | C | T | 15.9 | 2.0 | 7.89 | 10.77 | 1.03E-03 | T | C | 0.940 | 0.99 | 2 |
| 23 | rs5946005 | MIR448 MIR1911 MIR1298 IL13RA2 MIR1264 | 113988791 | G | A | 3.0 | 18.0 | 0.17 | 10.71 | 1.06E-03 | C | T | 0.644 | 1.02 | 2 |
| 23 | rs1335617 | IL13RA2 MIR448 MIR1911 MIR1298 RBMXL3 | 114084871 | G | A | 3.0 | 18.0 | 0.17 | 10.71 | 1.06E-03 | G | A | 0.651 | 1.02 | 2 |
| 23 | rs556677 | HTR2C SNORA35 MIR764 MIR1912 MIR1264 | 113729158 | T | C | 4.0 | 20.0 | 0.20 | 10.67 | 1.09E-03 | A | G | 0.985 | 1.00 | 2 |
| 6 | rs9296685 | TRAM2 | 52587141 | C | T | 62.0 | 104.0 | 0.60 | 10.63 | 1.11E-03 | *G* | *A* | *0.017* | 0.94 | 3 |
| 14 | rs11621184 | TMEM30B PRKCH | 60880705 | A | C | 46.0 | 83.0 | 0.55 | 10.61 | 1.12E-03 | *T* | *G* | *0.002* | 0.90 | 3 |
| 14 | rs1033910 | TMEM30B PRKCH | 60886593 | C | T | 83.0 | 46.0 | 1.80 | 10.61 | 1.12E-03 | *T* | *C* | *0.015* | 0.93 | 3 |
| 14 | rs4899272 | ACTN1 | 68462017 | G | T | 81.0 | 128.0 | 0.63 | 10.57 | 1.15E-03 | *A* | *C* | *0.003* | 1.08 | 3 |
| 7 | rs7755 | CD36 | 80144207 | A | G | 143.1 | 93.1 | 1.54 | 10.56 | 1.15E-03 | *T* | *C* | *0.022* | 1.07 | 3 |
| 12 | rs10848848 | TSPAN9 TEAD4 PRMT8 | 3300629 | C | T | 19.3 | 3.7 | 5.16 | 10.53 | 1.18E-03 | NA | NA | NA | NA | 2 |
| 22 | rs16987822 | VPREB1 PRAME GGTL4 ZNF280A ZNF280B | 21059244 | C | T | 2.3 | 16.3 | 0.14 | 10.53 | 1.18E-03 | C | T | 0.403 | 1.07 | 2 |
| 2 | rs13414323 | MYT1L | 2204217 | C | T | 13.6 | 1.2 | 11.73 | 10.49 | 1.20E-03 | T | C | 0.070 | 0.85 | 2 |
| 8 | rs2922362 | KHDRBS3 LOC286094 | 137039657 | A | G | 3.1 | 18.0 | 0.17 | 10.43 | 1.24E-03 | NA | NA | NA | NA | 2 |
| 12 | rs12314068 | SYT1 | 77729001 | C | T | 14.4 | 1.5 | 9.45 | 10.40 | 1.26E-03 | NA | NA | NA | NA | 2 |
| 10 | rs11256402 | SFTA1P LOC254312 | 10035873 | C | T | 3.3 | 18.3 | 0.18 | 10.40 | 1.26E-03 | G | A | 0.717 | 0.98 | 2 |
| 1 | rs6678986 | KMO FH | 239751454 | A | G | 13.2 | 1.0 | 12.89 | 10.39 | 1.26E-03 | NA | NA | NA | NA | 2 |
| 4 | rs11569033 | ELOVL6 EGF | 111125252 | A | G | 15.7 | 2.1 | 7.46 | 10.38 | 1.28E-03 | G | A | 0.940 | 0.99 | 2 |
| 1 | rs10926508 | KMO FH | 239761572 | A | G | 13.1 | 1.0 | 12.86 | 10.37 | 1.28E-03 | NA | NA | NA | NA | 2 |
| 11 | rs728748 | FJX1 LDLRAD3 TRIM44 | 35851245 | C | T | 3.0 | 17.7 | 0.17 | 10.37 | 1.28E-03 | NA | NA | NA | NA | 2 |
| 1 | rs6429278 | KMO FH | 239764442 | C | T | 1.0 | 13.1 | 0.08 | 10.36 | 1.28E-03 | NA | NA | NA | NA | 2 |
| 23 | rs1999925 | FAM133A NAP1L3 | 93215057 | G | A | 8.0 | 27.0 | 0.30 | 10.31 | 1.32E-03 | *G* | *A* | *0.048* | 0.94 | 3 |
| 1 | rs10159204 | SEC16B | 176182121 | A | G | 13.0 | 1.0 | 13.00 | 10.29 | 1.34E-03 | T | C | 0.195 | 1.13 | 2 |
| 2 | rs1430635 | CTNNA2 | 79540430 | C | T | 1.0 | 13.0 | 0.08 | 10.29 | 1.34E-03 | T | C | 0.779 | 1.03 | 2 |
| 3 | rs7645672 | SNAR-I GMNC OSTN CCDC50 UTS2D | 192218812 | G | T | 1.0 | 13.0 | 0.08 | 10.29 | 1.34E-03 | NA | NA | NA | NA | 2 |
| 16 | rs882820 | SRL TFAP4 | 4196006 | A | G | 81.0 | 45.0 | 1.80 | 10.29 | 1.34E-03 | *A* | *G* | *0.022* | 1.08 | 3 |
| 21 | rs12482513 | TIAM1 SOD1 KRTAP19-8 SCAF4 KRTAP11-1 | 31657678 | A | G | 13.0 | 1.0 | 13.00 | 10.29 | 1.34E-03 | *T* | *C* | *0.023* | 1.13 | 2 |
| 13 | rs566445 | TBC1D4 | 74769454 | C | T | 16.1 | 2.3 | 6.91 | 10.28 | 1.35E-03 | NA | NA | NA | NA | 2 |
| 5 | rs329298 | SAR1B CATSPER3 PCBD2 CAMLG TXNDC15 DDX46 SEC24A PHF15 | 133948902 | C | T | 56.7 | 27.3 | 2.08 | 10.28 | 1.35E-03 | *A* | *G* | *0.033* | 0.90 | 3 |
| 12 | rs17046362 | SYT1 PAWR | 78007172 | A | G | 1.0 | 13.0 | 0.08 | 10.27 | 1.35E-03 | NA | NA | NA | NA | 2 |
| 1 | rs3806368 | RGS5 | 161380502 | A | G | 18.0 | 43.0 | 0.42 | 10.25 | 1.37E-03 | *A* | *G* | *0.011* | 0.90 | 3 |
| 1 | rs3806365 | RGS5 | 161382055 | A | G | 43.0 | 18.0 | 2.39 | 10.25 | 1.37E-03 | *C* | *T* | *0.010* | 0.89 | 3 |
| 19 | rs7254060 | PEX11G ARHGEF18 INSR | 7234414 | A | G | 43.0 | 18.0 | 2.39 | 10.25 | 1.37E-03 | *T* | *C* | *0.010* | 1.14 | 3 |
| 2 | rs10196649 | BCL2L11 ACOXL | 111772761 | A | G | 2.5 | 16.3 | 0.15 | 10.22 | 1.39E-03 | NA | NA | NA | NA | 2 |
| 7 | rs10485846 | CNTNAP2 | 147385340 | A | G | 21.0 | 47.5 | 0.44 | 10.20 | 1.41E-03 | *T* | *C* | *0.022* | 0.90 | 3 |
| 3 | rs1317243 | CD200 | 113535936 | C | T | 132.0 | 85.0 | 1.55 | 10.18 | 1.42E-03 | *C* | *T* | *0.022* | 1.06 | 3 |
| 22 | rs5750432 | PPM1F VPREB1 TOP3B MAPK1 | 20732940 | C | T | 19.9 | 45.8 | 0.44 | 10.15 | 1.45E-03 | *C* | *T* | *0.049* | 0.92 | 3 |
| 16 | rs868511 | SRL TFAP4 | 4196464 | C | T | 84.2 | 47.7 | 1.77 | 10.11 | 1.47E-03 | *C* | *T* | *0.034* | 1.08 | 3 |
| 6 | rs4896789 | LOC100507557 EPM2A | 145616838 | C | T | 78.0 | 123.0 | 0.63 | 10.07 | 1.50E-03 | *T* | *C* | *0.026* | 1.07 | 3 |
| 12 | rs10861406 | OCC-1 | 104278417 | A | G | 82.0 | 128.0 | 0.64 | 10.06 | 1.52E-03 | *A* | *G* | *0.002* | 0.92 | 3 |
| 16 | rs12920415 | RNF166 MVD CYBA C16orf84 SNAI3 | 87289133 | A | G | 1.4 | 13.7 | 0.10 | 10.02 | 1.55E-03 | NA | NA | NA | NA | 2 |
| 22 | rs138936 | TSPO BIK TTLL12 MCAT | 41897880 | A | G | 3.6 | 18.4 | 0.19 | 10.02 | 1.55E-03 | NA | NA | NA | NA | 2 |
| 14 | rs4902672 | ACTN1 | 68462634 | C | T | 88.0 | 135.3 | 0.65 | 10.01 | 1.56E-03 | *G* | *A* | *0.002* | 0.92 | 3 |
| 23 | rs16998238 | DMD | 31833554 | C | A | 0.0 | 10.0 | 0.00 | 10.00 | 1.57E-03 | NA | NA | NA | NA | 2 |
| 23 | rs17318620 | TMEM47 FAM47A | 34362025 | C | T | 0.0 | 10.0 | 0.00 | 10.00 | 1.57E-03 | NA | NA | NA | NA | 2 |
| 2 | rs6749818 | POMC DNMT3A EFR3B RBJ | 25074026 | A | G | 25.1 | 52.9 | 0.47 | 9.95 | 1.61E-03 | *A* | *G* | *0.012* | 0.89 | 3 |
| 9 | rs7851726 | C9orf3 | 96543806 | C | T | 106.0 | 65.0 | 1.63 | 9.83 | 1.72E-03 | *A* | *G* | *0.048* | 0.95 | 3 |
| 1 | rs12736336 | FOXD3 | 63419788 | A | G | 27.1 | 55.3 | 0.49 | 9.70 | 1.85E-03 | *T* | *C* | *0.015* | 0.90 | 3 |
| 1 | rs12132140 | FCRL4 FCRL1 FCRL3 FCRL2 CD5L | 156033986 | C | T | 33.9 | 12.7 | 2.67 | 9.64 | 1.91E-03 | *A* | *G* | *0.000* | 0.79 | 3 |
| 22 | rs9622658 | SH3BP1 GGA1 | 36333570 | C | T | 128.0 | 83.0 | 1.54 | 9.60 | 1.95E-03 | *C* | *T* | *0.046* | 1.05 | 3 |
| 1 | rs4656538 | POU2F1 | 165526379 | A | G | 75.0 | 118.0 | 0.64 | 9.58 | 1.97E-03 | *T* | *C* | *0.001* | 0.92 | 3 |
| 2 | rs12621294 | LRRTM4 | 77284130 | A | G | 9.6 | 28.7 | 0.33 | 9.52 | 2.03E-03 | *A* | *G* | *0.045* | 0.85 | 3 |
| 3 | rs2103022 | LPP | 189553658 | A | G | 104.0 | 64.0 | 1.63 | 9.52 | 2.03E-03 | *T* | *C* | *0.000* | 1.13 | 3 |
| 6 | rs2749047 | TRAM2 | 52591374 | G | T | 44.9 | 79.2 | 0.57 | 9.51 | 2.04E-03 | *C* | *A* | *0.040* | 0.92 | 3 |
| 5 | rs11241496 | DMXL1 TNFAIP8 | 118602461 | A | G | 98.0 | 59.4 | 1.65 | 9.49 | 2.07E-03 | *G* | *A* | *0.004* | 0.92 | 3 |
| 1 | rs1021621 | POU2F1 | 165465160 | A | G | 115.0 | 73.0 | 1.58 | 9.38 | 2.19E-03 | *C* | *T* | *0.000* | 0.91 | 3 |
| 2 | rs17010833 | XPO1 KIAA1841 AHSA2 USP34 PUS10 PEX13 | 61656813 | A | G | 15.0 | 37.0 | 0.41 | 9.31 | 2.28E-03 | *C* | *T* | *0.018* | 1.15 | 3 |
| 8 | rs16917026 | PCMTD1 PXDNL ST18 | 53060033 | A | G | 15.0 | 37.0 | 0.41 | 9.31 | 2.28E-03 | *T* | *C* | *0.009* | 0.87 | 3 |
| 9 | rs1616208 | C9orf50 PTGES PRRX2 C9orf32 ASB6 C9orf106 | 131338554 | A | G | 78.0 | 121.0 | 0.64 | 9.29 | 2.30E-03 | *T* | *C* | *0.017* | 0.93 | 3 |
| 3 | rs4677056 | EIF4E3 GPR27 PROK2 FOXP1 | 72012211 | C | T | 112.9 | 71.6 | 1.58 | 9.22 | 2.39E-03 | *T* | *C* | *0.020* | 0.93 | 3 |
| 16 | rs4786475 | SRL TFAP4 | 4199779 | A | C | 85.8 | 50.5 | 1.70 | 9.17 | 2.46E-03 | *T* | *G* | *0.028* | 1.08 | 3 |
| 9 | rs12553834 | PAX5 | 36912489 | C | T | 123.0 | 80.0 | 1.54 | 9.11 | 2.54E-03 | *T* | *C* | *0.018* | 0.93 | 3 |
| 3 | rs12106673 | CD200 | 113544421 | A | G | 129.3 | 85.2 | 1.52 | 9.09 | 2.58E-03 | *T* | *C* | *0.015* | 1.07 | 3 |
| 4 | rs7678889 | RG9MTD2 C4orf17 MTTP | 100805038 | A | G | 56.4 | 93.3 | 0.60 | 9.07 | 2.59E-03 | *A* | *G* | *0.032* | 0.94 | 3 |
| 2 | rs4675377 | ICOS CTLA4 | 204522921 | C | T | 107.0 | 67.2 | 1.59 | 9.06 | 2.62E-03 | *C* | *T* | *0.000* | 1.16 | 3 |
| 2 | rs7592348 | MYO1B | 192046963 | A | C | 28.9 | 10.2 | 2.84 | 9.00 | 2.70E-03 | *C* | *A* | *0.041* | 0.82 | 3 |
| 16 | rs12927725 | ERCC4 MKL2 | 13860267 | C | T | 16.0 | 38.0 | 0.42 | 8.96 | 2.76E-03 | *C* | *T* | *0.012* | 0.87 | 3 |
| 2 | rs6414164 | ICOS CTLA4 | 204486845 | C | T | 47.2 | 81.0 | 0.58 | 8.94 | 2.79E-03 | *A* | *G* | *0.000* | 1.18 | 3 |
| 3 | rs355788 | DHX36 GPR149 SGEF | 155483306 | C | T | 70.3 | 39.1 | 1.80 | 8.92 | 2.81E-03 | *T* | *C* | *0.024* | 0.92 | 3 |
| 3 | rs355769 | DHX36 GPR149 SGEF | 155525244 | C | T | 70.3 | 39.1 | 1.80 | 8.92 | 2.81E-03 | *A* | *G* | *0.023* | 0.92 | 3 |
| 1 | rs870875 | CD247 | 165666358 | A | C | 74.0 | 115.0 | 0.64 | 8.89 | 2.86E-03 | *G* | *T* | *0.015* | 1.07 | 3 |
| 11 | rs1431572 | KIRREL3-AS3 KIRREL3 | 126978400 | C | T | 115.0 | 74.0 | 1.55 | 8.89 | 2.86E-03 | *G* | *A* | *0.034* | 1.06 | 3 |
| 7 | rs10954289 | CD36 | 80164219 | A | G | 91.2 | 136.1 | 0.67 | 8.88 | 2.89E-03 | *C* | *T* | *0.021* | 1.06 | 3 |
| 9 | rs356131 | FANCC C9orf3 | 96607148 | A | G | 69.0 | 108.5 | 0.64 | 8.83 | 2.96E-03 | *T* | *C* | *0.033* | 0.94 | 3 |
| 6 | rs10498982 | EPHA7 TSG1 | 93405563 | A | G | 79.0 | 121.0 | 0.65 | 8.82 | 2.98E-03 | *T* | *C* | *0.035* | 0.95 | 3 |
|  |  |  |  |  |  |  |  |  |  |  |  |  |  |  |  |

T and U – the number of heterozygous parents who transmit the alleles A1 and A2, respectively, T and U are the expected transmission counts. T/U – transmission odds based on the expected transmission counts.

**Inclusion criteria Number of SNPs**

**1)** Our GWAS p<0.0003 477

**2)** Our GWAS 5<OR<0.2 53

**3)** Combined p<10-5 (Our GWAS p<0.003 with Dubois et al. GWAS p<0.05) 73
